# The predicted interactome of the human mitochondrial proteome

**DOI:** 10.1038/s41467-026-77112-z

**Published:** 2026-08-25

**Authors:** Abhinav B. Swaminathan, Mohammad Zulkifli, Rachel M. Guerra, Sofia M. Calabrese, Dimitris T. Kalafatis, David J. Pagliarini, Vishal M. Gohil

**Affiliations:** 1 Department of Biochemistry and Biophysics, Texas A&M University, College Station, TX USA; 2 Department of Cell Biology and Physiology, Washington University School of Medicine, St. Louis, MO USA; 3 Department of Biochemistry and Molecular Biophysics, Washington University School of Medicine, St. Louis, MO USA; 4 Department of Genetics, Washington University School of Medicine, St. Louis, MO USA; 5 Howard Hughes Medical Institute, Washington University School of Medicine, St. Louis, MO USA

**Keywords:** Mitochondrial proteins, High-throughput screening

## Abstract

Despite the fundamental importance of mitochondria in cellular metabolism, the molecular function(s) of many mitochondrial proteins remain unknown. Since protein function can be inferred from their interacting partners, we repurpose the protein structure prediction algorithm AlphaFold Multimer (AFM) as a classification model to predict protein-protein interactions of the entire human mitochondrial proteome. By screening 630,003 protein pairs, we create a compendium of 2,895 previously known and newly observed interactions, which include the interacting partner(s) of 85 uncharacterized mitochondrial proteins, thereby linking them to a known biochemical pathway. Extending the AFM-based analysis to 11 diverse eukaryotes identifies evolutionarily conserved interactions among human hits, including regulators of core bioenergetic pathways. Our experiments, guided by these predictions, nominate protein interactions that form the coenzyme Q metabolon and define the mitochondrial copper delivery pathway to cytochrome *c* oxidase. Our compendium represents a powerful resource for the systematic, structure-based functionalization of the human mitochondrial proteome.

## Abstract (web summary)

The molecular function of many mitochondrial proteins remains unknown. Here, the authors present MitoMatch, an AlphaFold Multimer-based compendium of mitochondrial protein interactions that links orphan proteins to known pathways, defines the CoQ metabolon, and reveals the role of COA4 in copper delivery to cytochrome *c* oxidase.

## Introduction

Mitochondria house core metabolic pathways for cellular energy generation and biosynthesis of cofactors essential for cellular and organismal health^1^. Pathogenic mutations have been identified in almost 30% of the ~1100 mitochondrial proteins that result in common human diseases, as well as rare inborn errors of metabolism^2–4^. Despite their critical roles in cellular functions and human disease, approximately 10% of the human mitochondrial proteome remains orphan without an associated pathway annotation or biochemical function^5^. Consequently, regulators and components of some of the core mitochondrial metabolic pathways remain to be discovered.

The availability of a well-defined inventory of mitochondrial proteins called MitoCarta^5,6^ has spurred many targeted and high-throughput loss-of-function studies to determine the function of these poorly characterized proteins^7–15^. Complementary approaches, such as yeast two-hybrid and co-immunoprecipitation(co-IP)-based protein-protein interaction studies, and more recently, complexome profiling, have been used to elucidate the function of uncharacterized proteins through the guilt-by-association principle^16–18^. However, these approaches are laborious, time-consuming, and often contaminated with false positives^19,20^, which limits their application in annotating protein functions in a high-throughput manner. Thus, there is a need for orthogonal approaches to systematically determine the function of uncharacterized mitochondrial proteins.

AlphaFold-Multimer (AFM) is a neural network model trained to predict the structures of multimeric protein complexes of known stoichiometry and composition^21^. Recent studies applying AFM to small datasets have uncovered its potential to identify interacting partners of a query protein^22–24^. However, the utility of AFM for a large-scale, ab initio identification of protein–protein interactions remains to be systematically evaluated. In this study, we demonstrate that AFM can be repurposed as a discovery tool that can predict binary protein-protein interactions in the mitochondrial proteome with 85% precision. Our dataset, which we have named MitoMatch (mitomatch.web.app), consists of 2895 interactions and includes at least one interacting partner for 85% of orphan mitochondrial proteins. We demonstrated the utility of this resource through experimental validation of our predictions by nominating direct protein-protein interactions that form the coenzyme Q metabolon, and by placing poorly characterized human mitochondrial protein, COA4, at a specific step in the mitochondrial copper delivery pathway. MitoMatch is a compendium of organelle-wide, direct protein-protein interactions to link mitochondrial proteins to known pathways and help assign their role(s) in mitochondrial functions.

## Results

### MitoMatch: human mitochondrial protein interactome

**Fig. 1:**
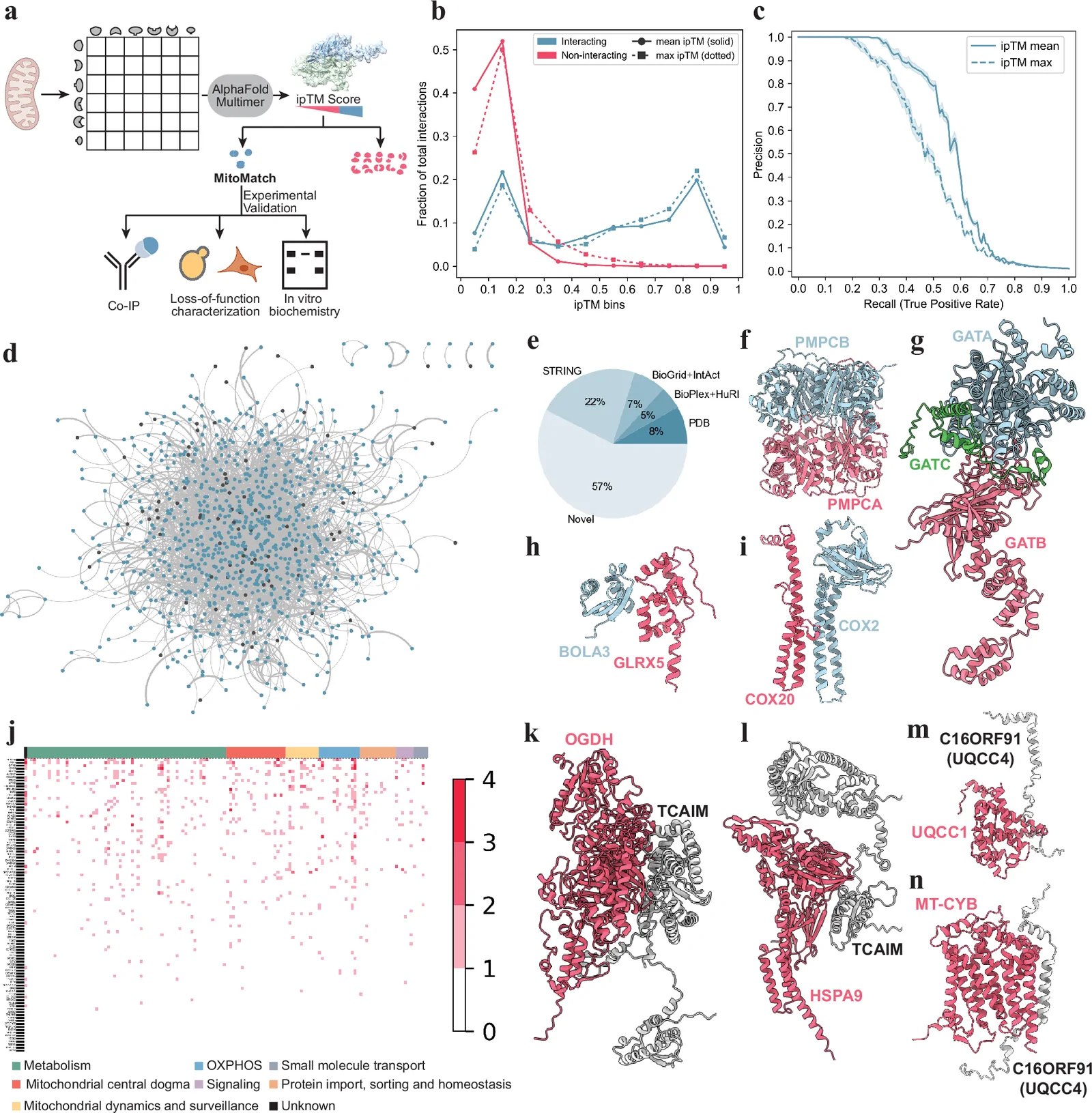
Using AlphaFold-Multimer as a classification model to predict mitochondrial protein-protein interactions. **a** A schematic showing the application of AlphaFold-Multimer as a classifier to predict protein-protein interactions based on its output: the interface predicted template modeling (ipTM) score and follow-up experimental validation. Parts of this image were created in BioRender. Swaminathan, A. (2026) https://BioRender.com/lkvpwsc and Swaminathan, A. (2026) https://BioRender.com/l0d23t7. **b** A plot of the number of protein-protein interactions that fall into different ipTM bins for the recent PDB dataset. Solid line represents the mean ipTM score, and dotted line represents the best ipTM score from five different predicted models of interactions. The interacting and non-interacting groups have sample sizes of 1338 and 15,005 protein pairs, respectively. **c** Precision-recall curve of **b** at a 1:100 ratio of true:false pairs. The shaded part of the curve indicates a confidence interval of 95%. **d** Interaction network of predicted mitochondrial protein-protein interactions. Proteins are represented as circles, and the interactions are represented as gray lines. The thickness of lines is proportional to the mean ipTM scores. Blue dots represent proteins with a MitoCarta pathway annotation, and black dots represent uncharacterized proteins. **e** A pie chart of the predicted mitochondrial interactions mapped to known databases. Predicted structures of the **f** mitochondrial processing peptidase complex (PMPCB-PMPCA), **g** glutamyl-t-RNA(Gln) amidotransferase complex (GATA-GATB-GATC), **h** interaction between Fe-S cluster biogenesis factors (BOLA3-GLRX5), and **i** cytochrome *c* oxidase assembly intermediate (COX2-COX20). **j** A heat map depicting the predicted interactions for uncharacterized mitochondrial proteins across different MitoCarta Pathways. Predicted structures of **k** TCAIM-OGDH, **l** TCAIM-HSPA9, **m** C16ORF91-UQCC1, and **n** C16ORF91-MT-CYB interactions. Source data are provided as a Source Data file. Co-IP co-immunoprecipitation.

We first asked if AFM^21^ can be repurposed as a classification model that predicts whether two proteins can interact based on the interface predicted template modeling (ipTM) score (Fig. 1a). The ipTM score is a confidence metric that was originally designed to assess the accuracy of predicted multimeric structures. We asked if we could repurpose this metric as a classifier cutoff score to distinguish interacting from non-interacting protein pairs. In this case, a score of 0 would indicate no interaction, whereas a score of 1 would indicate a high-confidence interaction. To accomplish this, we compiled a non-redundant list of 1338 binary interacting pairs and 15,005 non-interacting pairs from recently deposited PDB structures that were not used for training AFM (Supplementary Notes). We ran all five multimer models of AFM with one random seed, producing five predictions in total for each interaction. We found that the interacting protein pairs have a bimodal distribution with one prominent peak at ipTM >0.8 and another peak at an ipTM <0.2, whereas the non-interacting protein pairs have a single peak at ipTM <0.2 (Fig. 1b). The choice of using either the maximum ipTM score or the average of all five ipTM scores did not markedly affect the distribution of the interacting pairs; however, the right-sided shoulder of the non-interacting peak was more restricted when using the mean ipTM score as compared to using just the maximum score (Fig. 1b). This suggests that for a high-throughput application of AFM, the mean ipTM score will be more informative in eliminating false positives than simply considering the best model with the maximum score. This is also reflected in the precision-recall curve, where using the mean ipTM as a classifier metric yielded ~40% of all true positives at 90% precision whereas the best ipTM score yielded only 30% of the true hits at the same precision (Fig. 1c). We analyzed the key parameters that could influence ipTM scores and found that the primary determinant of AFM success is the multiple sequence alignment (MSA) depth, where interacting pairs with more than 25 diverse sequences perform well, with median ipTM scores of more than 0.6, which steadily increases with increasing MSA depth (Supplementary Fig. 1a, b and Supplementary Notes). Our analysis further revealed that a total number of interface residues of greater than 20 and lower global stoichiometry, defined as the total number of subunits in the protein complex, also increased the ipTM score (Supplementary Fig. 1c–f and Supplementary Notes).

Given that AFM predicts protein-protein interactions with higher precision when the above-mentioned parameters are met, we asked if it can be applied to the human mitochondrial proteome. To this end, we first determined the MSA depth of human mitochondrial protein pairs and found that more than 97% of protein pairs have an MSA depth of at least 25 sequences (Supplementary Fig. 2a). Next, we set out to determine the ipTM cut-off score to separate true interactors from non-interacting protein pairs. To this end, we curated a small test dataset of mitochondrial protein-protein interactions that have been experimentally validated (Supplementary Table 1) and compared their mean and max ipTM score distribution in the background of non-interacting pairs of mitochondrial proteins. Consistent with our previous observation (Fig. 1b), we find that using the mean ipTM score is better at separating interacting from non-interacting pairs (Supplementary Fig. 2b). The first false positive appears around a mean ipTM score of about 0.5 (Supplementary Fig. 2b, bottom panel), therefore, we decided to use 0.5 as a cutoff to separate interacting from non-interacting pairs, which gives 67% recall at a false discovery rate of about 15% (Supplementary Fig. 2b, c).

MitoCarta3.0, the most well-curated inventory of the human mitochondrial proteome, lists 1136 mitochondrial proteins^5^. Of these, 101 proteins are completely uncharacterized without any pathway annotation. Even in cases where proteins have a pathway annotation, there are many instances where their molecular function(s) or their precise position in the pathway remain obscure. To functionalize the mitochondrial proteome, we applied AFM to predict the interactome of the human mitochondrial proteins. We first parsed the MitoCarta3.0 dataset by excluding the proteins that have non-canonical amino acids and proteins with more than 2000 amino acids to overcome AFM and computational limitations, respectively, and generated a curated list of 1123 proteins (~99% of mitochondrial proteins) for further analysis (Supplementary Methods). We applied AFM to predict interacting partners for each of these 1123 proteins by pairing each protein with every other protein from this list to generate 630,003 protein pairs (see Zenodo deposition 21232148). AFM predicted most protein pairs to be non-interacting, with 541,758 protein pairs (86%) having an ipTM score of less than 0.2 (Supplementary Fig. 2d). Based on our cutoff of 0.5 mean ipTM score, we predict 2895 interactions (0.46%) involving 1004 proteins at an apparent FDR of 15% (Fig. 1d and Supplementary Data 1). The median number of interacting partners for these 1004 proteins is four (Supplementary Fig. 2e). These 2895 interactions constitute MitoMatch - a compendium of predicted protein-protein interactions across the entire human mitochondrial proteome.

Next, we asked if our compendium recapitulates known interactions from existing protein-protein interaction databases. Mapping our dataset against STRING, BioGRID, IntAct, BioPlex, HuRI, and PDB recovered 43% of interactions predicted by MitoMatch; the remaining 57% were not present in these databases and thus represent previously unreported interactions (Fig. 1e and Supplementary Data 2). We also completely recapitulated 56% of individual mitochondrial complexes reported in the recently released ‘complex portal’ dataset^25^, with an average coverage of 77% proteins per complex (Supplementary Fig. 3). This includes large mitochondrial protein complexes, including the mitochondrial ribosomes and oxidative phosphorylation (OxPhos) complexes for which experimentally resolved structures exist (Supplementary Fig. 3). Importantly, MitoMatch provides structures for interactions for which none exists. For example, MitoMatch predicts the structures of the mitochondrial processing peptidase complex (Fig. 1f and Supplementary Fig. 4a), glutamyl-t-RNA(Gln) amidotransferase complex (Fig. 1g and Supplementary Fig. 4b, c), interaction between Fe-S cluster biogenesis factors BOLA3 and GLRX5 (Fig. 1h and Supplementary Fig. 4d), and the interaction between mitochondrial OxPhos protein COX2 with its assembly factor COX20 (Fig. 1i and Supplementary Fig. 4e). In addition to recovering these known protein-protein interactions, we identified at least one interacting partner for 85 uncharacterized proteins that were distributed across multiple mitochondrial pathways (Fig. 1j). Among these, we recapitulated interactions reported in two recent studies that linked the uncharacterized protein TCAIM to OGDH regulation^26^ (Fig. 1k, l and Supplementary Fig. 4f, g) and C16ORF91 to MT-CYB assembly^27^ (Fig. 1m, n and Supplementary Fig. 4h, i), cross validating these new discoveries.

### Evolutionarily conserved interactions

**Fig. 2:**
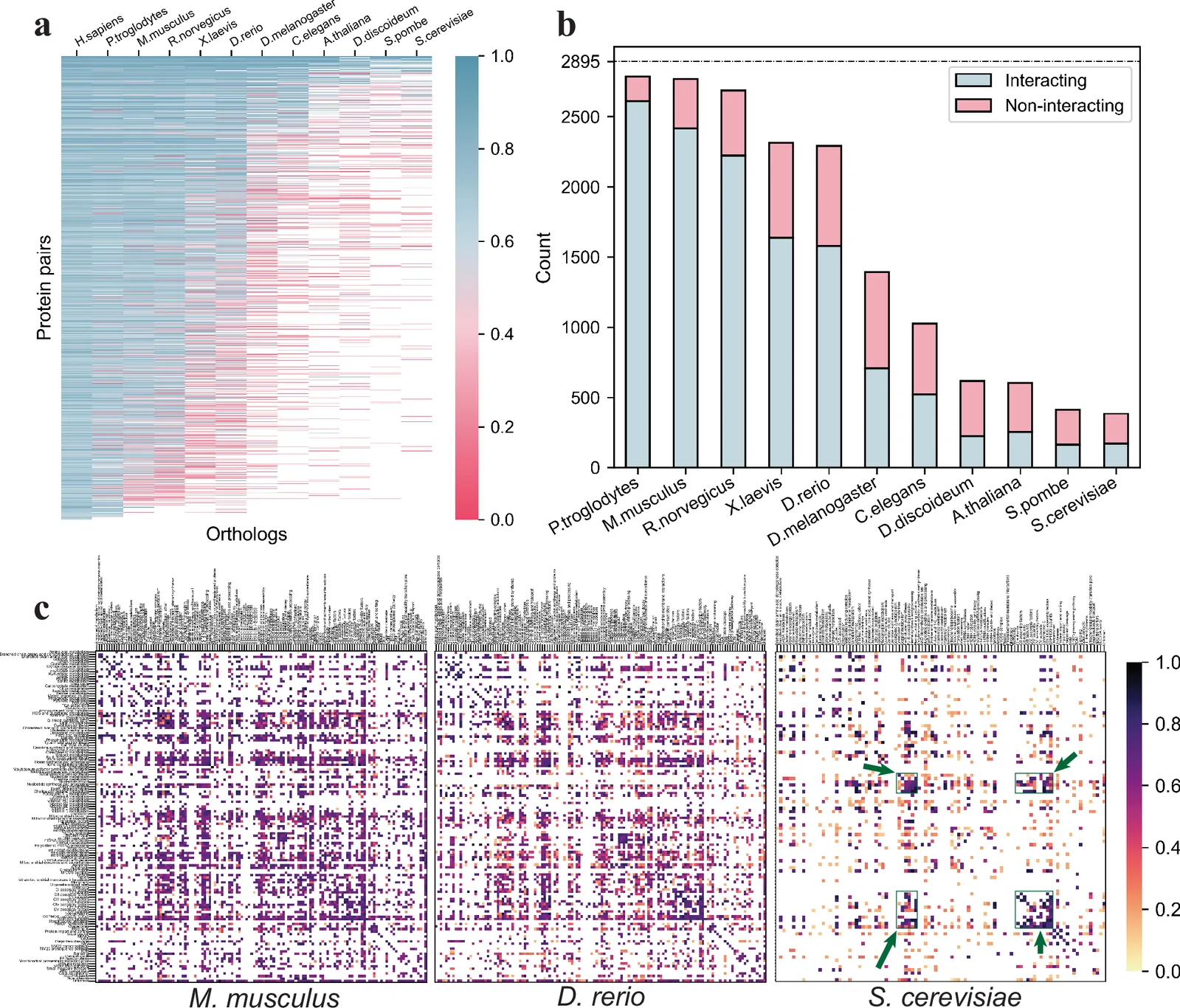
Evolutionarily conserved interactions across different mitochondrial pathways. **a** A heatmap of ipTM scores of 2895 interactions between human mitochondrial protein pairs mapped to 11 indicated organisms. The color bar represents the ipTM scores and the lack of color indicates the absence of a homologous protein pair in that species. **b** A bar chart depicting the number of homologous pairs predicted to be interacting or not in the indicated species. **c** Heatmap depicting the median ipTM scores of interactions between proteins of each mitochondrial pathways in the indicated organisms. The boxes highlighted by green arrows in the ‘Yeast’ heatmap show clusters of interactions between pathways that are highly conserved from humans to yeast as explained in the text. Source data are provided as a Source Data file.

To investigate what fraction of the predicted human mitochondrial protein interactions are conserved we mapped our 2895 high-confidence interactions to 11 other evolutionarily distant eukaryotic organisms—Chimpanzee (*P. troglodytes*), Mouse (*M. musculus*), Rat (*R. norvegicus*), Frog (*X. laevis*), Fish (*D. rerio*), Fly (*D. melanogaster*), Worm (*C. elegans*), Slime Mold (*D. discoidium*), Plant (*A. thaliana*), Fission yeast (*S. pombe*), and Baker’s yeast (*S. cerevisiae*, henceforth called yeast) (Supplementary Table 2). To do this, we first identified homologs of each of 1123 human mitochondrial proteins in these organisms using the reciprocal best-hit search (Supplementary Fig. 5a and Supplementary Methods). We then mapped each protein in 2895 human protein pairs to the proteome of these 11 organisms resulting in 2787, 2768, and 383 protein pairs in chimpanzee, mouse, and yeast, respectively (Supplementary Fig. 5b). We find a strong correlation between ipTM scores of the human hits and higher eukaryotes, and that decreases with more distant homologs (Fig. 2a, Supplementary Fig. 6 and Supplementary Data 3). Specifically, AFM predicts about 93%, 87%, and 44% of the homologous hits to be interacting in chimpanzee, mouse, and yeast, respectively (Fig. 2b). In addition, we also devised two metrics to prioritize interactions for downstream experimental follow-up. First, we provide a species score, which is calculated as the sum of ipTM scores across all species containing homologs of both proteins in the query pair. Second, we provide a conservation score which represents the fraction of homologous pairs identified as hits in the screen (ipTM ≥0.5). Higher scores in both these metrics indicate greater confidence in the predicted interaction across species (Supplementary Data 2).

Next, to determine if these evolutionarily conserved protein-protein interactions were enriched in certain mitochondrial processes and pathways, we analyzed all 149 pathways listed in MitoCarta3.0^5^. A distinct high ipTM score pattern emerges at the diagonal, which indicates that interactions between proteins in the same pathway are more conserved than others (Fig. 2c and Supplementary Fig. 7). We also observed heatmap signatures where interactions of proteins belonging to two different pathways were conserved across eukaryotes, which is more apparent in the yeast heatmap (Fig. 2c). These were predominantly interactions between proteins belonging to OxPhos subunits and their corresponding assembly factors, and with proteins involved in the biogenesis and trafficking of metal cofactors such as copper, Fe-S clusters, and heme (depicted as green arrows in Fig. 2c).

### Using MitoMatch to define the molecular interactions of the coenzyme Q complex

**Fig. 3:**
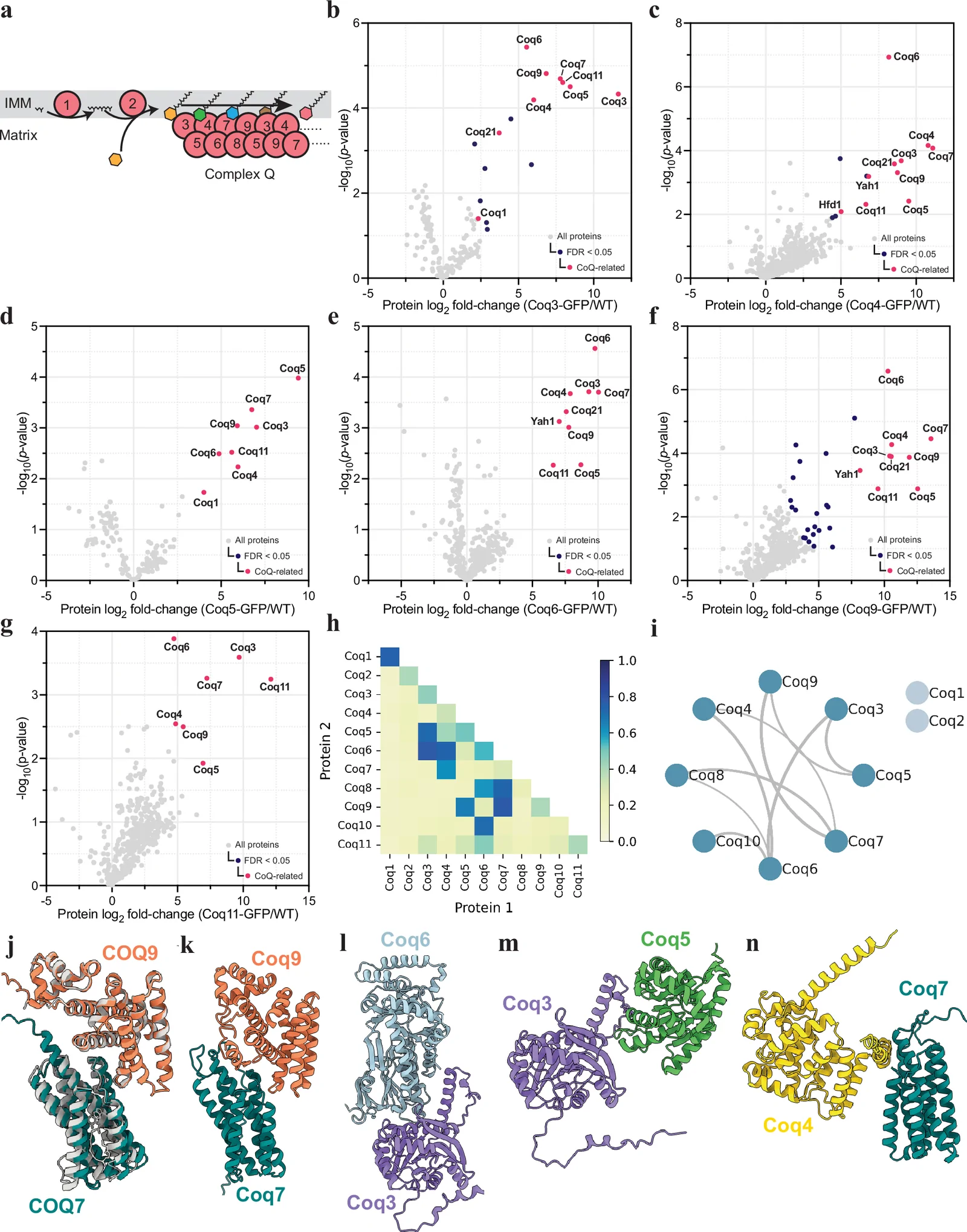
Predicting binary interactions of proteins in mitochondrial complex Q. **a** A schematic representation of mitochondrial coenzyme Q biosynthetic pathway involving Coq1–Coq9 proteins. **b**–**g** Volcano plot obtained from immunoprecipitation of the indicated GFP-tagged Coq protein followed by mass spectrometry analysis (*n* = 3 biological replicates). The data were normalized to immunoprecipitation performed in WT mitochondria. Statistical analysis was performed using a two-sided Student’s *t* test. **h** Heatmap depicting the mean ipTM scores of pairwise interactions of yeast proteins involved in CoQ biosynthesis. **i** An interaction network of yeast CoQ proteins based on the ipTM scores from (**h**). **j** Superposition of experimentally resolved human COQ7-COQ9 interaction (white) with the predicted COQ7-COQ9 structure (green-orange). **k**–**n** Predicted structures of the binary interactions of the indicated yeast Coq proteins. Source data are provided as a Source Data file. ipTM interface predicted template modeling score.

To demonstrate the utility of MitoMatch, we focused on the biosynthetic pathway of coenzyme Q (CoQ, ubiquinone), a quinone-isoprenoid mobile electron carrier that transfers electrons from OxPhos complexes I/II to III. The biosynthesis of CoQ requires the concerted action of multiple evolutionarily conserved Coq proteins^28^. PDSS1/2 (named Coq1 in *S. cerevisiae*) synthesizes the polyprenyl pyrophosphate tail, which gets attached to the CoQ head-group precursor 4-hydroxybenzoic acid by Coq2 (Fig. 3a). Coq3-7 modify this precursor head-group via a series of reactions further aided by Coq8, 9, and 11, to produce the mature CoQ molecule^28^ (Fig. 3a). These head-group modifying proteins are thought to form a dynamic metabolon at the matrix-facing side of the IMM, called complex Q (also called the CoQ-synthome)^28^.

To identify all the components of the CoQ metabolon, we performed co-IP of endogenously tagged Coq1-6, Coq8-9, and Coq11 proteins from chemically cross-linked yeast mitochondria (Fig. 3b–g and Supplementary Fig. 8). Coq1 and Coq2 did not co-immunoprecipitate with any other Coq proteins, consistent with the idea that only proteins related to head-group modification form the metabolon (Supplementary Fig. 8a, b). Coq3-6, Coq9, and Coq11 consistently co-immunoprecipitated Coq3-7, Coq9, and Coq11, suggesting that these proteins form a core complex amongst themselves (Fig. 3b–g). We observed additional known auxiliary factors involved in CoQ biosynthesis, such as Yah1 and Coq21, in some of our co-IP experiments (Fig. 3c, e, f). Coq8, an ATPase that supports CoQ biosynthesis, only captured Coq3 and itself (Supplementary Fig. 8c). This is also consistent with prior studies demonstrating that Coq8 activity is required for metabolon formation and activity^29^, but likely only exhibits transient interactions with metabolon proteins.

Although crosslinking mass spectrometry helped identify proteins forming a complex Q metabolon, this approach cannot define direct binary interactions. To delineate direct interactors from indirect association, we applied AFM to predict binary interactions amongst known Coq pathway proteins in yeast (Fig. 3h, i). Since our experimental conditions above may preclude select interactions, we included the full set of Coq1-11 in our AFM-based analyses to identify binary interactors (Fig. 3h). This identified nine high-confidence interactions among Coq proteins consistent with our co-IP data. Importantly, many of these interactions were also identified in their human counterparts from our MitoMatch compendium (Supplementary Fig. 9a, b). To date, only one structure of a complex Q interaction has been solved—the complex between COQ7 and its auxiliary factor COQ9^30^. Encouragingly, our analyses accurately predict the individual human structures of these proteins and their experimentally defined binding interface (Fig. 3j and Supplementary Fig. 10a) and reveal a highly similar complex for the yeast proteins (Fig. 3k and Supplementary Fig. 10b). Our second top prediction was between Coq3 and Coq6 (Fig. 3h, l and Supplementary Fig. 10c), which was also reported in a recently available preprint^31^. Given that Coq3 and Coq6 function sequentially in the CoQ biosynthesis pathway, this interaction is biochemically meaningful^28^. Beyond these, we reveal strong predictions for binary interactions between head group-modifying enzymes Coq3-Coq5 and Coq4-Coq7 (Fig. 3m, n and Supplementary Fig. 10d, e), and between enzymes and auxiliary factors, including Coq6-8, Coq5-9, and Coq7-8 (Supplementary Fig. 11a–c). These predictions may help explain recent observations regarding the ability of Coq8 to augment the Coq6-mediated reaction using ancestrally reconstructed versions of the Coq proteins^32^. Finally, our work predicts a robust interaction between Coq6 and Coq10 (Supplementary Fig. 11d), the latter being a CoQ-binding protein whose current function is poorly understood, thereby motivating functional hypotheses.

While this general approach can be extended to predict larger oligomeric complexes, recent analyses suggest that the complex Q metabolon is most likely a “statistical complex” with multiple conformations rooted in a smaller number of robust binary interactions^31^. Collectively, our work here serves to define the complex Q membership and to nominate select interactions as the anchoring interactions within the metabolon.

### Using MitoMatch to define the mitochondrial copper delivery pathway

**Fig. 4:**
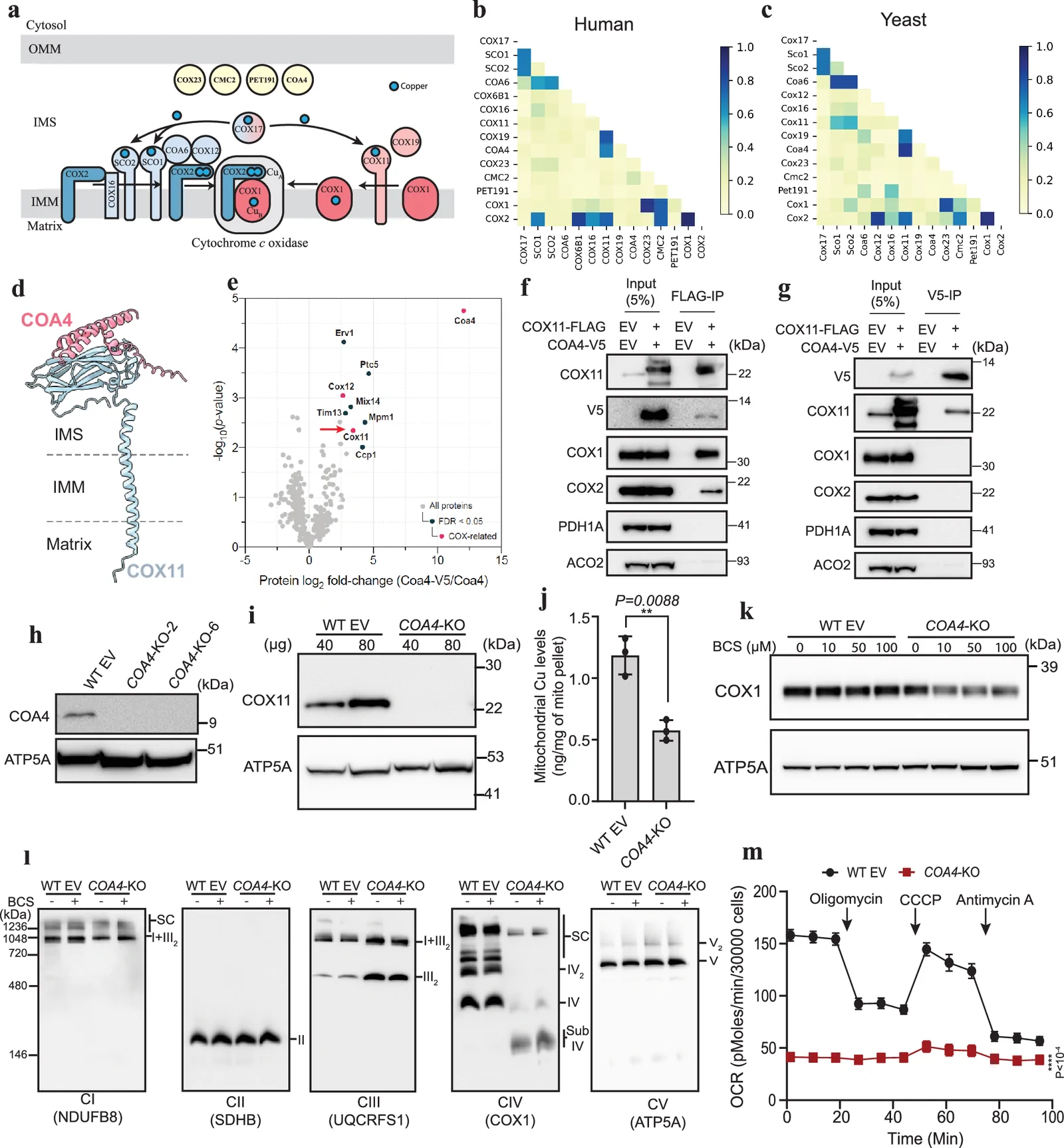
Placing COA4 in the mitochondrial copper delivery pathway to cytochrome *c* oxidase through the identification of its physical interactor. **a** A schematic representation of the mitochondrial copper delivery pathway to cytochrome *c* oxidase (CcO). Copper delivery to COX1 and COX2 subunits of CcO requires metallochaperones—COX17, SCO1, and COX11—that bind and transfer copper to their target proteins, and accessory factors such as COX16, COX19, SCO2, and COA6 that enable this transfer. Intermembrane space (IMS) localized CcO assembly factors depicted in yellow circles are hypothesized to play a role in copper delivery. **b** A heatmap of ipTM scores of the indicated human and **c** yeast protein pairs that are either a part of the mitochondrial copper delivery pathway or are implicated in the copper delivery process. The color bar represents the mean ipTM score. **d** Predicted structure of COA4-COX11 interaction. **e** A volcano plot of the relative abundance of proteins immunoprecipitated from V5-tagged Coa4 yeast mitochondria compared to untagged mitochondria versus statistical significance (*n* = 3 biological replicates). Statistical analysis was performed using a two-sided Student’s *t* test. **f** Co-immunoprecipitation of COX11-FLAG (*n* = 3) and **g** COA4-V5 from digitonin solubilized mitochondria of 293T cells transfected with COA4-V5 and COX11-FLAG followed by western blotting (*n* = 3). **h** SDS-PAGE/western blot analysis of COA4 from WT and COA4-KO MCH58 cell lines. ATP5A is used as a loading control (*n* = 2). **i** SDS-PAGE/western blot analysis of COX11 in solubilized mitochondria isolated from WT and COA4-KO-6 MCH58 cells (*n* = 3). **j** Mitochondrial Cu levels measured in WT and COA4-KO-6 mitochondria using inductively coupled plasma–mass spectrometry (*n* = 3). Data are presented as mean ± SD. Statistical analyses were performed using an unpaired two-tailed Student’s *t* test. **k** SDS-PAGE/western blot analysis of COX1 from WT and COA4-KO-6 cells treated with different concentrations of the copper chelator BCS (*n* = 3). **l** BN-PAGE/western blot analysis of WT and COA4-KO-6 mitochondria isolated from cells grown with and without BCS (*n* = 2). **m** Oxygen consumption rate measurements of WT and COA4-KO-6 MCH58 cells following treatment with ATP synthase inhibitor (Oligomycin), uncoupler (CCCP), and electron transport inhibitor (Antimycin A) (*n* = 3). Data are presented as mean ± SEM. Statistical analyses were performed using ordinary one-way ANOVA. All sample sizes in this figure represent biological replicates from independent experiments. Statistical significance levels: **p < 0.01; ****p < 0.0001. Western blots are representative of the indicated number of independent biological replicates. Source data are provided as a Source Data file. OMM outer mitochondrial membrane, IMM inner mitochondrial membrane.

Next, we applied predictions from MitoMatch to elucidate the structural basis of the mitochondrial copper delivery pathway to the OxPhos complex IV, also known as cytochrome *c* oxidase (CcO). CcO is the copper-containing enzyme of the mitochondrial electron transport chain that uses ~90% of all oxygen consumed by the cell to drive mitochondrial ATP synthesis^33^. The core subunits of CcO, COX1 and COX2 contain three copper ions that are essential for its catalytic activity and stability. The delivery and insertion of copper into CcO subunits is a complex process that requires copper-metallochaperones and accessory proteins^34^. These proteins are localized to the mitochondrial intermembrane space (IMS) and the inner mitochondrial membrane (IMM) and together constitute the mitochondrial copper delivery pathway (Fig. 4a). Despite decades of work, this pathway is not fully understood with many other poorly characterized, evolutionarily conserved CcO assembly factors such as COX23, CMC2, PET191, and COA4 implicated in copper delivery, though their precise position in the pathway remains unresolved^35–38^. To define this pathway, we used AFM to analyze the interactions between all known proteins of the human copper delivery pathway, along with these four uncharacterized proteins. AFM successfully predicted 8 of the 12 known canonical interactions in the pathway (Fig. 4b), most of which were conserved in yeast (Fig. 4c). Importantly, the predicted models provide a structural basis for many steps in the pathway that have evaded structural studies. These include key steps in copper delivery to the COX2 subunit involving metallochaperones, COX17, and SCO1, and accessory proteins SCO2, COA6, and COX16 (Supplementary Figs. 12 and 13).

Given that AFM-predicted structural models are consistent with the molecular function of experimentally characterized proteins, we wanted to extend the utility of AFM in placing uncharacterized proteins in the copper delivery pathway. We focused on IMS-localized CcO assembly factors, COX23, CMC2, PET191, and COA4, whose roles in CcO assembly are not known. AFM predicted evolutionarily conserved interactions between COX23 and COX1, CMC2 with COX2, and COA4 with COX11 (Fig. 4b, c). Since copper metallochaperone COX11 is the core member of the mitochondrial copper delivery pathway, we focused on the COA4-COX11 interaction. The predicted structure of this interaction is consistent with the localization and topology of these proteins, where IMS-localized COA4 interacts with the IMS-facing domain of COX11 (Fig. 4d and Supplementary Fig. 13f). We experimentally validated our prediction by performing co-immunoprecipitation (Co-IP) of yeast Coa4-V5 from chemically cross-linked mitochondria, followed by mass spectrometric analysis, which identified Cox11 as its interacting partner (Fig. 4e). In addition, we also find other proteins enriched in our Co-IP-MS data, including the IMS-localized Ptc5 phosphatase, which suggests a role for this protein in regulating copper delivery to CcO through reversible phosphorylation of Coa4 in yeast. To validate this interaction in humans, we also performed co-IP in mitochondria isolated from 293T cells transfected with COA4-V5 and COX11-FLAG constructs. Co-IP of COX11-FLAG using Anti-FLAG beads recovered COA4-V5 as an interacting partner, validating our prediction (Fig. 4f). We also recovered COX1 as an interacting partner of COX11 (Fig. 4f), which serves as a positive control because COX11 is a known metallochaperone of COX1. A recent study has shown that COX1 and COX2 assembly processes are highly coordinated, with COX11 also interacting with newly synthesized COX2^39^. This is also consistent with our MitoMatch dataset, which predicts an interaction of COX11 with COX2 (Fig. 4b). Importantly, we also recover COX2 as an interactor of COX11 in our co-IP (Fig. 4f), validating both our prediction and reproducing prior results from others^39^. In a reciprocal IP experiment using Anti-V5 beads, we identified COX11-FLAG as an interacting partner of COA4 (Fig. 4g), again validating MitoMatch prediction. Notably, unlike with COX11-IP, we did not recover COX1 and COX2 proteins in our COA4-V5-IP, which is in line with our MitoMatch dataset that does not predict a direct interaction between COA4 and either COX1 or COX2.

To understand the biological significance of this interaction, we next set out to determine if the human COA4 plays a role in copper delivery to CcO. To this end, we generated a CRISPR-Cas9-based knockout of COA4 in a human fibroblast cell line, which showed a complete absence of COA4 in two independent COA4-KO clones (Fig. 4h). Consistent with the evidence of direct interaction between COA4 and COX11, we find that the loss of COA4 results in a striking reduction in COX11 abundance (Fig. 4i). Loss of COA4 also results in reduced mitochondrial copper content without impacting the levels of other transition metals (Fig. 4j and Supplementary Fig. 14). Since our AFM prediction implicates COA4 in copper delivery to COX1 through its interaction with COX11, we argued that COA4-KO cells would be sensitive to copper limitation. Indeed, we find that the treatment with increasing concentrations of the copper chelator, bathocuproinedisulfonic acid (BCS), results in a more pronounced reduction in COX1 abundance in COA4-KO cells as compared to the wild-type (WT) cells (Fig. 4k). Failure of copper delivery to COX1 is known to reduce its abundance. To assess if COA4-KO cells exhibit reduced abundance of COX1-containing complexes, we performed BN-PAGE followed by western blotting and found a drastic and specific reduction in the levels of complex IV-containing supercomplexes in COA4-KO mitochondria (Fig. 4l). This decreased abundance of complex IV expectedly resulted in reduced respiration in COA4-KO cells (Fig. 4m). Taken together, our AFM prediction not only provided the structural basis of known aspects of the mitochondrial copper delivery pathway but also guided experiments that placed the uncharacterized human COA4 protein in the COX11-mediated copper delivery pathway to CcO.

## Discussion

In an era of artificial intelligence where millions of protein structures are now available through machine learning algorithms such as AlphaFold2 and ESMFold, defining their functions is now a grand challenge in biology^40,41^. Even for one of the best studied organelles, such as the mitochondria, almost 100 resident proteins remain completely uncharacterized without an associated pathway annotation or biochemical function^5^. Here, we applied the machine learning algorithm AFM to generate a high-confidence compendium of protein-protein interactions for the entire human mitochondrial proteome. This repository, called MitoMatch, consists of 2895 predicted interactions which includes at least one interacting partner  for 85% of uncharacterized mitochondrial proteins. Guided by these predictions, we defined binary interactions and membership amongst the CoQ metabolon proteins and assigned human COA4 to a specific step in copper delivery to cytochrome *c* oxidase. Thus, MitoMatch provides a resource for annotating uncharacterized mitochondrial proteins by identifying the proteins they interact with. A predicted interaction with a characterized protein serves as a starting point for hypothesis generation—the orphan protein may function as a subunit of the same pathway, a regulatory factor, a chaperone, or an assembly factor.

One of the strengths of AFM-based protein-protein interaction predictions is that they solely rely on evolutionary signals found within and between proteins to predict interaction interfaces in protein pairs through residue coevolution and are agnostic to biological or thermodynamic constraints. As a result, a limitation of this approach is that it cannot provide binding affinities of the interaction pair. Further, we note that many of our predicted interactions could happen in the cell in the context of a larger complex and may not exist as true heterodimers. Thus, our predictions indicate whether a given protein pair can interact, but not the biological conditions, stoichiometry, or the complex composition in which that interaction takes place.

Although AFM was originally developed to predict the structure of multimeric protein complexes of known stoichiometry, a few recent studies have explored the potential of AFM to identify new protein-protein interactions^22–24,42^. For example, a recent study utilized AFM to predict the interacting partners of a protein called DONSON that was previously implicated in DNA replication^24^. This in-silico screen targeted against ~70 core replication factors identified other replication initiation proteins as interacting partners of DONSON. However, extending this approach to an unbiased screening against the entire human proteome was not successful^24^. In our study, we adopt two simple approaches to enable the successful application of AFM to the entire mitochondrial proteome. First, since cognate interacting partners should be co-localized, we restricted our search grid to ~1100 mitochondrial proteins instead of the entire human proteome, achieving a ~20-fold enrichment in true interactions to begin with. Second, we increased the stringency of our cutoff by considering a consensus among all five multimer models by taking an average of the ipTM scores, thereby reducing false positives (Fig. 1c). Similarly, a recent study developed a scoring algorithm to increase the accuracy of AFM predictions on human proteins by integrating biological information about the protein pairs, such as their co-localization, co-expression, and co-dependency from the DEPMAP dataset^43^. A more recent approach utilized deeper MSAs and a finetuned version of RoseTTAFold as a classifier to predict protein-protein interactions for the human proteome, which recovered 8-22% of true positives^44^. Compared to these approaches that are currently only applicable to the human proteome, our pipeline is broadly applicable and can be extended to any organismal proteome, thereby providing a simple and generalized framework that improves the accuracy of AFM-based predictions.

Consistent with a previous study^45^, which showed that genes belonging to cofactor and energy metabolism in yeast are replaceable with their human counterparts, we find that evolutionarily conserved interactions are also highly enriched in these pathways (Fig. 2c). Building on this finding, we provide two experimental vignettes that exemplify the utility of MitoMatch. In the first example, we focused on defining the molecular interactions that underpin the complex Q metabolon. This problem is two-fold—first requiring the identification of the components of complex Q, followed by identifying direct interactions amongst these proteins. We partly address this problem through cross-linking co-immunoprecipitation mass spectrometry experiments that identified the Coq proteins that are a part of the complex Q metabolon (Fig. 3b–g). However, this experimental data, just like every other co-IP dataset, does not distinguish direct interactions from indirect associations. To address this, we leveraged AFM to predict binary interactions amongst the yeast Coq proteins, identifying nine high-confidence protein-protein interactions amongst Coq3-10 proteins (Fig. 3h, i). Many of these interactions are also conserved from yeast to humans (Supplementary Fig. 9). Importantly, AFM accurately reproduces an experimentally resolved structure of COQ7-COQ9 interaction, while also predicting a similar structure for the yeast counterpart (Fig. 3j, k). This experimental structure of COQ7-COQ9 was released after the training cutoff date for AFM^21,30^, and therefore serves as an unbiased validation, highlighting the robustness with which this approach can be applied to discover protein-protein interactions. Together, we nominate several binary interactions that could serve as anchor points for the formation of the coenzyme Q metabolon. For our second application, we focused on assigning a protein to a specific step in the mitochondrial copper delivery pathway to CcO. AFM predicted an evolutionarily conserved interaction between the mitochondrial protein COA4 and COX11, the copper metallochaperone for CcO^46^ (Fig. 4b–d). Co-IP confirmed this interaction (Fig. 4e–g) and provided a biochemical basis for a prior observation showing rescue of the respiratory growth defect of yeast *coa4Δ* by Cox11 overexpression^38^. Together, these results placed COA4 in a COX11-dependent step of copper delivery to CcO. Based on these experimental vignettes, we anticipate that MitoMatch will greatly accelerate the functionalization of the human mitochondrial proteome.

## Methods

### AlphaFold Multimer pipeline for high-throughput protein-protein interaction prediction

We used AlphaFold Multimer algorithm to generate multiple sequence alignments (MSA) of input protein sequences. Since MSA generation for a protein is independent of what protein it is paired with, we split the AlphaFold Multimer pipeline into MSA generation and prediction steps, thereby eliminating the time-consuming steps of repeated MSA generation for each prediction. In the MSA generation step, we compute alignments using the default Multimer pipeline, identify templates with a max cut-off date of 2018-04-30, generate features, and store the features as a pickle file. In the prediction step, we read these pickle files to make use of these precomputed alignment features for all input proteins, and the default prediction pipeline is followed including MSA pairing and structure prediction.

### Curating a recent PDB dataset for using AlphaFold Multimer as a classification model

Our recent PDB dataset consists of dimers, binary pairs from oligomeric complexes, and non-interacting protein pairs from the PDB. Since AlphaFold-Multimer v2.2 was trained on structures deposited before 2018-04-30, we only considered structures deposited after this date for the recent PDB dataset. We downloaded the PDB mmcif files and PDB bioassembly files on 2022-12-18, our “recent” PDB data consisted of protein structures deposited between these two dates. We only considered bioassemblies that had a global stoichiometry between 2–100 chains, discarding monomers. For bioassemblies that had more than 2 chains, we decomposed them into binary pairs by pairing each protein chain with every other protein chain in that assembly and categorizing them into interacting and non-interacting protein pairs. We consider a protein pair, say ‘A-B’, as interacting if at least one heavy (non-hydrogen) atom of protein ‘A’ is within 4 Å of another heavy atom of protein ‘B’. We enforced the following criteria for any protein pair to be included further - both proteins should be an L-polypeptide containing only 20 canonical amino acids, each should have a length of at least 50 amino acids and a total length of less than 2000 amino acids, and the pair should be heteromers and from the same species. These were then further filtered with a strategy similar to what DeepMind used to validate their Multimer model^29^. Briefly, we performed sequence alignment using MMseqs2 against sequences of the training dataset (structures deposited before 2018-04-30) and removed any protein pair, even if only one of the proteins has more than 40% sequence identity to the training dataset. For the dimer dataset, we simply filtered protein pairs whose global stoichiometry is 2 subunits. For binary protein pairs from oligomers, we only considered core interactions in the complex that are both minimalistic and essential to build the oligomeric complex (refer Supplementary Methods). For the non-interacting dataset, we selected protein pairs that are not interacting.

For each of these datasets, we clustered the proteins at 40% sequence identity using MMseqs2 and gave each cluster an identifier. We then mapped the protein pair to a cluster pair, which is simply the union of the cluster identifiers of its individual proteins. We grouped all protein pairs that belong to a cluster pair and randomly selected one representative interaction from each cluster. This resulted in 110 binary pairs from dimers and 1228 binary pairs from oligomers. For the non-interacting dataset, after mapping a protein pair to a cluster pair, we only selected those clusters where all protein pairs in that cluster were non-interacting and randomly selected a representative protein pair from each cluster pair, resulting in 15,509 pairs. Finally, we mapped these pairs to the entire PDB regardless of the release date and removed any pair whose proteins share sequence homology with another interacting pair in any structure, resulting in 15,005 non-interacting protein pairs.

### Mitochondrial test dataset

We manually curated a list of 12 interactions that were experimentally validated in the copper delivery pathway to the mitochondrial cytochrome *c* oxidase (Supplementary Table 1). Apart from COX1-COX11 interaction, all other interactions were previously validated using purified proteins. Many of these interactions are thought to be of a transient nature, and most of them do not have an experimentally solved structure. These 12 interactions constitute the interacting dataset. To generate a non-interacting dataset, we reasoned that proteins involved in copper delivery to CcO will not interact with proteins of OxPhos complex I, II, III, and V because only complex IV contains copper. Therefore, we paired 7 proteins in MitoCarta3.0 that were annotated as “Metabolism > Metals and cofactors > Copper metabolism” with 115 proteins annotated as OXPHOS complex I, II, III, and V subunits or their assembly factors, resulting in 805 non-interacting pairs.

### Calculation of total interface residues

The total interface residues of an interacting protein pair A-B are defined as the sum of interacting residues in both proteins A and B. An interacting residue of protein A is defined as any residue in protein A whose heavy atoms are within 4 Å to any heavy atom in protein B, and vice versa. We implemented a Python script using the NeighborSearch function of BioPython for this calculation (Supplementary Methods).

### Calculation of median per-residue effective (N_eff_) multiple sequence alignment (MSA) depth

The default setting of AFM involves pairing MSA of both query proteins from the same species while block-diagonalizing the rest. Since the paired MSA part is rich in coevolutionary information between two proteins, we calculated the MSA depth of the paired MSA part only. We first filtered the paired MSA from the total MSA. For each residue in the query protein, we only consider those sequences that have a non-gap residue at that position in the MSA. We then cluster these sequences at 80% sequence identity and count the number of clusters, which gives us the effective number of sequences or the effective MSA depth at that position. We do this for each residue of the query sequence, and the median of all the effective MSA depths at each position is referred to as the median per-residue N_eff_ MSA depth. Details of the calculation, along with the code, are provided in supplementary methods.

### Human mitochondrial interaction prediction

We obtained the list of 1136 human genes encoding mitochondrial proteins from the MitoCarta3.0 repository. We updated the genes whose UniProt IDs were missing or outdated in MitoCarta3.0 and removed unreviewed entries that could not be mapped to reviewed ones. We then removed proteins that were more than 2000 amino acids long and those that contained non-canonical amino acids. This resulted in 1123 proteins. Heteromeric combinations of these proteins resulted in 630,003 binary protein pairs that were used for our mitochondrial protein-protein interaction prediction.

### Identifying homologs of human mitochondrial proteins in other eukaryotic organisms

The human proteome and proteomes of the 11 other eukaryotic organisms were downloaded from UniProt. We performed a ‘reciprocal best hit’ search to identify the homologs of human mitochondrial proteins. Briefly, we performed a sequence alignment of each human mitochondrial “query” protein against the proteome of the target eukaryotic organism using Mmseqs2 and retrieved the best hit (lowest e-value). This best hit is considered the homolog of the query protein if a sequence alignment of this best hit against the human proteome retrieves the query protein as the best hit. Since we already begin with a human mitochondrial interaction dataset that is enriched in true interactions, we relaxed our criteria for identifying evolutionary hits by considering an interaction in the homologous pairs to be true if at least one predicted model has an ipTM score above our threshold of 0.5.

### Yeast culturing

YPD media was prepared with 1% yeast extract, 2% peptone, and 2% glucose, while YPGE media was prepared with 1% yeast extract, 2% peptone, 3% Glycerol, and 1% ethanol. The media pH was adjusted to 5.5. Yeast primary cultures were grown in YPD media overnight at 30 °C. Secondary cultures were inoculated at 0.1 OD_600nm_ and were grown in YPGE media at 30 °C till the mid-log phase, and cells were harvested.

### Yeast transformation

Yeast transformation was performed as described before^47^. Briefly, cells were grown in YPD overnight at 30 °C and were washed and resuspended in one-step buffer (0.2 N Lithium acetate, 40% PEG 3350, and 100 mM dithiothreitol (DTT)) along with 1 µg of plasmid DNA and 50 µg of salmon sperm DNA. The mixture was incubated at room temperature for 25 min, 42 °C for 20 min, followed by incubation on ice for 5 min. Cells were then pelleted, washed, and plated on appropriate selection plates and incubated at 30 °C for about 2–3 days to obtain colonies.

### Mammalian cell culture

The human MCH58 cells were cultured in high-glucose Gibco Dulbecco’s Modified Eagle Medium (DMEM, Thermo #11995065) media supplemented with 10% fetal bovine serum (FBS) (Sigma #F2442). The 293T cells were cultured in the same media but with an additional supplementation of 2 mM Glutamine (Thermo #25030081). Cells were cultured in 5% CO_2_ at 37 °C.

### Mammalian CRISPR-Cas9-based gene knockout

We used lentiCRISPRv2 plasmid (Addgene #52961) to construct CRISPR/Cas9-based *COA4* knockout in human MCH58 cell line. The guide RNA sequences targeting *COA4* gene are: sgCOA4_1For:CACCGACGGGTGAAGAAAGACGATG sgCOA4_1Rev:AAACCATCGTCTTTCTTCACCCGTC sgCOA4_2For:CACCGAGGCCATACCTGGACCCAAC sgCOA4_2Rev:AAACGTTGGGTCCAGGTATGGCCTC

sgCOA4_3For:CACCGCAGGTGCAGGCGTTCAAGGA

sgCOA4_3Rev: AAACTCCTTGAACGCCTGCACCTGC

LentiCRISPRv2 plasmid vector backbone was digested using BsmBI restriction enzyme followed by T4 ligation of the digested vector with the phosphorylated and annealed oligo pairs. The ligated constructs were amplified in the Stbl3 *Escherichia coli* strain and sequence verified. Lentiviral particles were prepared using standard protocols. Transduction using the lentivirus was performed in MCH58 WT cells using 8 μg/mL polybrene (EMD Millipore). After 24-h incubation, the virus-containing medium was removed, and a fresh culture medium containing puromycin/blasticidin was added and selected for 72 h. Cells were then trypsinized, diluted, and plated at a density of 1 cell per well in 96-well plates. A clonal population was established in the selection medium containing 2.5 µg/ml puromycin. Disruption of the COA4 expression was confirmed by western blotting.

### Mitochondrial isolation

Crude mitochondria from yeast cells were isolated as described previously^48^. Yeast cells were grown in 100–500 mL of YPGE media and were harvested at mid-log phase. The cell pellet (2–10 g) was incubated in DTT buffer (100 mM Tris-HCl, pH 9.4, 10 mM DTT, at 2 mL/g of cells) for 20 min at 30 °C. Cells were then pelleted, washed in zymolyase buffer (1.2 M sorbitol, 20 mM potassium phosphate, pH 7.4 at 7 mL/g of cells), and resuspended in zymolyase buffer containing 3 mg zymolyase (US Biological Life Sciences) per gram of cell pellet. The cell suspension was incubated at 30 °C for 45 min. The efficiency of digestion was checked spectrophotometrically by diluting zymolyase-treated cells in water and measuring optical density at 600 nm. Spheroplasts, thus obtained, were pelleted at 3000 × *g* for 5 min and were homogenized in homogenization buffer (0.6 M sorbitol, 10 mM Tris-HCl, pH 7.4, 1 mM EDTA, 1 mM PMSF, 0.2% (w/v) BSA (essentially fatty acid-free, Sigma-Aldrich) at 6.5 mL/g of cells) with 15 strokes using a glass Teflon homogenizer with pestle B. The homogenate was then diluted in an equal volume of homogenization buffer and centrifuged at 1500 × *g* for 5 min at 4 °C. The supernatant was centrifuged again at 4000 × *g* for 5 min at 4 °C, and the final supernatant was centrifuged at 12,000 × *g* at 4 °C for 15 min to pellet mitochondria. Mitochondria were resuspended in SEM buffer (250 mM sucrose, 1 mM EDTA, 10 mM MOPS-KOH, pH 7.2, containing 1× EDTA-free protease-inhibitor cocktail from Roche).

Crude mitochondria from mammalian cells were isolated using the Abcam mitochondrial isolation kit for cultured cells (ab110170) by following the manufacturer’s instructions. Protease inhibitor was added to all the buffers used (1× EDTA-free protease-inhibitor cocktail from Roche). Briefly, cells harvested from 2–4 15-cm plates were incubated in buffer A for 10 min and homogenized with 30 strokes. The solution was centrifuged at 1000 × *g* for 10 min and the supernatant (SN1) was collected while the pellet was resuspended in buffer B and homogenized with 30 strokes. The supernatant (SN2) was collected by pelleting the solution at 1000 × *g* for 10 min. The supernatants SN1 and SN2 were combined and pelleted at 12,000 × *g* for 15 min to obtain the crude mitochondrial pellet. The pellet was resuspended in buffer C and was flash-frozen in liquid nitrogen and stored at −80 °C.

### Co-immunoprecipitation experiments

For the yeast co-immunoprecipitation (co-IP) experiments, mitochondria isolated from yeast cells expressing GFP-tagged or V5-tagged target proteins were first cross-linked with the 0.5 mM DSSO (Thermo #A33545) crosslinker for 1 h at room temperature. The crosslinking was quenched with 100 mM Tris (pH 8.0) buffer.

For co-IP experiments in human cell line, 293T cells (ATCC CRL-3216) were transfected with COA4-V5-pcDNA3.1-Blast and COX11-FLAG-pcDNA3.1-Neo using PEIMAX (Polysciences # 24765-100). Briefly, 15 million 293T cells cultured in 15 cm plates were transfected using 15 µg of each plasmid and 90 µl of PEIMAX(1 µg/µl) for 48 h and the cells were harvested by scraping. Mitochondria (~2 mg) isolated from these cells were used for each co-IP experiment.

Mitochondria were solubilized with digitonin and incubated with magnetic beads conjugated with anti-V5 or anti-GFP or anti-FLAG antibodies. The antibody-conjugated beads were pulled down with a magnet. The beads were washed, and the proteins were analyzed using mass spectrometry or western blotting. All Co-IP experiments were performed with *n* = 3 biological replicates. Statistical analysis was performed using a two-sided Student’s *t* test.

### Crosslinking affinity enrichment mass spectrometry

Crude mitochondria were isolated from yeast (expressing GFP-tagged or V5-tagged target proteins) as described above and subjected to chemical crosslinking (1 mg mitochondria, 0.5 mM DSSO (Thermo, catalog no. A33433), 1 h, room temperature [r.t.]). Crosslinking was quenched with 100 mM Tris pH 8.0 followed by centrifugation (15,000 × *g*, 5 min, 4 °C). Mitochondria were then solubilized with 50 mM imidazole, 500 mM 6-hexaminocaproic acid, 1 mM EDTA and 1 g/g digitonin. The bait protein and crosslinked interactors were then enriched by anti-GFP or anti-V5 immunoprecipitation (IP) using magnetic GFP-Trap or V5-Trap agarose beads (Chromotek, catalog no. gtma or v5ta), respectively, washed and subjected to on-bead tryptic digest. The on-bead crosslinked proteins were denatured with 2 M urea in 200 mM Tris pH 8.0, then reduced with 5 mM DTT (30 min, 56 °C) and alkylated with 15 mM iodoacetamide (30 min, r.t., in the dark). The proteins on-bead were digested (overnight, 37 °C) with 1 μg trypsin (Promega, catalog no. V5113). The digested supernatant was acidified with 10% Trifluoroacetic acid (TFA) to a pH of 2 and desalted with 10 mg StrataX solid phase extraction columns (Phenomenex), then dried under vacuum using a SpeedVac (Thermo Scientific) and stored at −80 °C until MS analysis.

Samples were resuspended in 0.2% formic acid and subjected to LC–MS analysis. LC separation was performed using the Thermo Ultimate 3000 RSLCnano system. A 15 cm EASY-Spray PepMap RSLC C18 column (150 mm × 75 μm, 3 μm) was used at 300 nL/min flow rate with a 90 min gradient using mobile phase A consisting of 0.1% formic acid in H_2_O, and mobile phase B consisting of 0.1% formic acid in acetonitrile (ACN)/H_2_O (80/20, v/v). An EASY-Spray source was used at 35 °C. Each sample run was held at 4.0% B for 5 min and increased to 50% B over 65 min, followed by 8 min at 95% B and back to 4% B for equilibration for 10 min. An Acclaim PepMap C18 HPLC trap column (20 mm × 75 μm, 3 μm) was used for sample loading. MS detection was performed with a Thermo Exploris 240 Orbitrap mass spectrometer in positive mode. The source voltage was set to 1.8 kV, ion transfer tube temperature was set to 275 °C, RF lens was at 70%. Full MS spectra were acquired from *m*/*z* 350 to 1400 at the Orbitrap resolution of 60,000, with the normalized automatic gain control target of 300% (3 × 10^6^). Data-dependent acquisition (DDA) was performed for the top 20 precursor ions with the charge state of 2–6 and an isolated width of 2. Intensity threshold was 5 × 10^3^. Dynamic exclusion was 30 s with the exclusion of isotopes. Other settings for DDA include Orbitrap resolution of 15,000 and high-energy collision-induced dissociation energy of 30%.

Raw files were analyzed by SequestHT search engine incorporated in Proteome Discoverer v.2.5.0.400 software against yeast databases downloaded from Uniprot. Label-free quantification was enabled in the searches. The resulting data were analyzed by Perseus v.1.6.15.0 software^49^.

### Inductively coupled plasma–mass spectrometry

Metals (Cu, Fe, Zn, Mn) levels were quantified by inductively coupled plasma mass spectrometry (ICP-MS) using a NexION 300D instrument (PerkinElmer). Briefly, 60–100 mg of mitochondria were collected and washed twice with 1 mL of 300 mM mannitol containing 100 μM EDTA prepared in ultrapure, metal-free water (Trace SELECT; Sigma), followed by two additional washes with 300 mM mannitol to remove residual EDTA. After washing, pellets were weighed and digested in 40% (w/v) nitric acid (Trace SELECT; Sigma) at 90 °C for 18 h. This was followed by an additional 4 h digestion with 0.75% H₂O₂ (Sigma Supelco). The digested samples were then diluted to a final volume of 5 mL with ultrapure water prior to ICP-MS analysis.

### Oxygen consumption rate measurements

Oxygen consumption rate (OCR) measurements were carried out in intact cells using Seahorse XFe24 extracellular flux analyzer (Agilent Technologies). Briefly, cells were seeded in XF24-well cell culture microplates (Agilent Technologies) at 20,000 cells/well in 250 µl of growth media in high glucose DMEM growth media supplemented with 10% FBS and incubated at 37 °C in a 5% CO_2_ incubator for ∼20 h. Before measurements, 525 µl of the pre-warmed growth medium was added to each well, and cells were further incubated at 37 °C for 30 min in a non-CO_2_ incubator. Mix, wait, and measure timings were set to 2, 2, and 2 min, respectively. For the mitochondrial stress test, oligomycin, carbonyl cyanide 3-cholorophenylhydrazone (CCCP), and antimycin A were sequentially injected to achieve final concentrations of 0.5, 20, and 1 µM, respectively.

### SDS-PAGE, BN-PAGE, and western blotting

Protein concentrations in cellular or mitochondrial lysates were measured using Pierce BCA Protein Assay Kit (Thermo Fisher Scientific). The samples for sodium dodecyl sulfate-polyacrylamide gel electrophoresis (SDS-PAGE) were first denatured by the addition of LDS buffer and reducing agent followed by heating at 70 °C for 10 min. A total of 20 µg of the denatured protein extracts were then separated using NuPAGE 12% Bis-Tris gels (Thermo Fisher Scientific) and blotted onto a polyvinylidene difluoride (PVDF) membrane.

Blue native-polyacrylamide gel electrophoresis (BN-PAGE) samples were prepared by solubilizing crude mitochondria in 1% digitonin, followed by incubation on ice for 30 min and centrifugation at 20,000 × *g* for 30 min. The supernatant was collected, and G-250 sample additive was added to it. The solubilized mitochondrial proteins (20 µg) were separated using 3–12% Bis-Tris NativePAGE gel (Thermo Fisher Scientific) and were transferred onto a PVDF membrane for western blotting.

All PVDF membranes were blocked for 1 h in 5% (w/v) nonfat milk dissolved in Tris-buffered saline with 0.1% (w/v) Tween 20 (TBST-milk), followed by overnight incubation with a primary antibody in TBST-milk at 4 °C. Primary antibodies were used at the following dilutions. COA4, 1:500 (ab105678; Abcam); Anti-V5, 1:1000 (R96025; Invitrogen); COX11, 1:500 (11498-1-AP; ProteinTech); PDH1A, 1:1000 (ab168379; Abcam); NDUFB8, 1:1000 (ab110242; Abcam) SDHB, 1:1000 (ab14714; Abcam); UQCRFS1, 1:1000 (ab14746; Abcam); COX1, 1:1000 (ab14705; Abcam); COX2, 1:1000 (ab198286; Abcam); ATP5A, 1:20,000 (ab14748; Abcam); ACO2, 1:500 (MA535527; Thermo Fisher Scientific). After incubation with the primary antibody, the membranes were washed five times with TBST containing (0.1% TWEEN 20) buffer for 5 min each and incubated at room temperature with (1:5000) horseradish peroxidase-conjugated secondary antibody (anti-rabbit, Cat. No. 32460, Thermo Fisher Scientific or anti-mouse, Cat. No. 95017-332, VWR) prepared in 5% TBST-milk. After 60 min, the secondary antibody was discarded, and the membranes were washed five times with TBST buffer for 5 min each. Secondary antibodies (GE healthcare) were used at 1:5000 dilution in TBST-milk for 1 h at room temperature. Membranes were developed using Clarity Western ECL substrate (Bio-Rad, #1705060), or Clarity Max Western ECL substrate (Bio-Rad, #1705062). Uncropped blots can be found in the Source Data file.

### Statistical analysis

GraphPad Prism v11.0 software was used to plot graphs and bar charts. Statistical analyses were performed using unpaired two-tailed Student’s *t* test and the level of significance was indicated as stars representing *p* values in the figures. The bar height and error bars in bar graphs of experimental data represent mean and standard deviation, respectively, from 3 biological replicates. Co-variates such as cell line passage number were accounted for by using similar passages for control and knockout lines. Effect sizes are reported as the log₂ fold change (*x*-axis) in the volcano plots of immunoprecipitation–mass spectrometry experiments (Figs. 3b–g and 4e). For other quantitative comparisons, effect sizes correspond to the difference in group means, which can be derived from the mean and standard deviation values provided in the source data. Additionally, the number of biological replicates for each figure are provided in the figure legends.

### Figure generation

The data from our computational work were plotted and figures were generated using matplotlib v3.10.5 and seaborn v0.12.2 modules in Python. The protein-protein interaction network was created using Cytoscape v10.3 (https://cytoscape.org/). Protein structures were visualized using UCSF ChimeraX v1.8 (https://www.cgl.ucsf.edu/chimerax/). Graphs related to experimental data were plotted using GraphPad Prism v11.0.

### Reporting summary

Further information on research design is available in the Nature Portfolio Reporting Summary linked to this article.

## Supplementary information

Supplementary Information

Description of Additional Supplementary Files

Supplementary Data 1

Supplementary Data 2

Supplementary Data 3

Reporting Summary

Transparent Peer Review file

## Source data

Source data

## Data Availability

The computational data generated in this study have been deposited in the Zenodo database under accession code 21232148. This includes the predicted structures, confidence metrics such as pLDDT and predicted aligned error data. The structures and the confidence metrics can also be downloaded from the MitoMatch website [https://mitomatch.web.app]. The Mass Spectrometry data relevant to the Co-IP experiments have been deposited in the MassIVE database under accession code MSV000102370 [https://massive.ucsd.edu/ProteoSAFe/dataset.jsp?task=ed4f2a9140d44b61ade526149edaf3d8]. Source data for the figures in this manuscript are also provided as a Source Data file. Reagents generated in this study are available upon request. Source data are provided with this paper.

## Code availability

All original code has been provided in the Supplementary Methods or in Zenodo (https://doi.org/10.5281/zenodo.21232148).
