# Supplementary material for: The predicted interactome of the human mitochondrial proteome
Source: Nat Commun. 2026 Aug 25;17:10162. doi: 10.1038/s41467-026-77112-z (PMC13601606; doi:10.1038/s41467-026-77112-z)
Supplement: Supplementary file 1 [file 41467_2026_77112_MOESM1_ESM.pdf]

Supplementary Figures

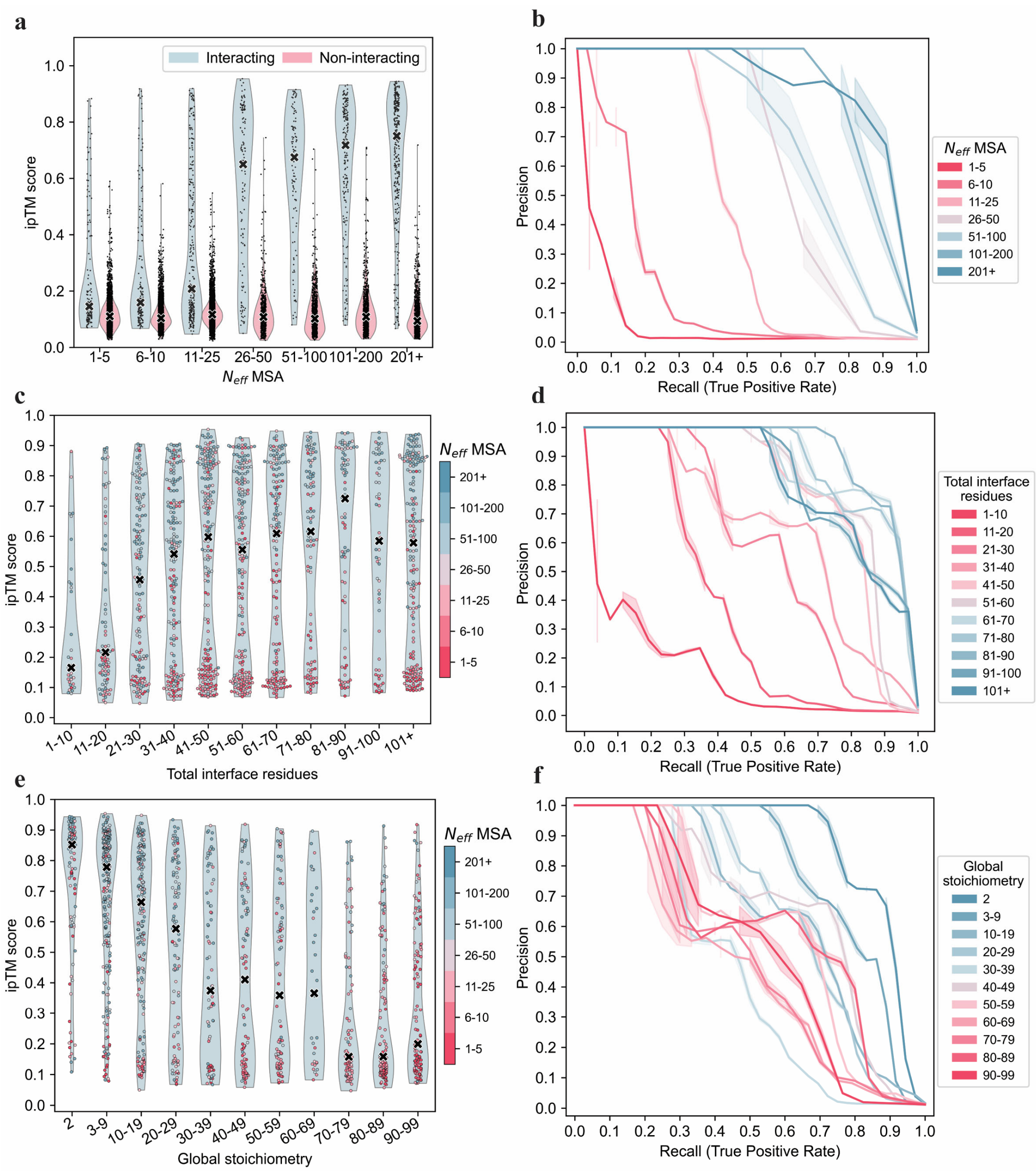

Supplementary Fig. 1: Parameters determining the success of AlphaFold Multimer.

a A plot of ipTM scores as a function of binned MSA depth. **b** A precision-recall curve as a function of MSA depth. **c** A plot of ipTM scores as a function of binned total interface residues. **d** A precision-recall curve as a function of binned total interface residues from the dataset in (**c**), after filtering for datapoints with at least 25 N_{eff} MSA depth. **e** A plot of ipTM scores as a function of binned global stoichiometry of macromolecular complexes from which binary interaction pairs are extracted. **f** The dataset in (**e**) was filtered for datapoints that have at least 25 N_{eff} MSA depth and a precision-recall curve was plotted as a function of binned global stoichiometry. All precision-recall curves were plotted at a ratio of 1:100 true:false interactions. The 'X' denotes the median of each violin. Source data are provided as a Source Data file.

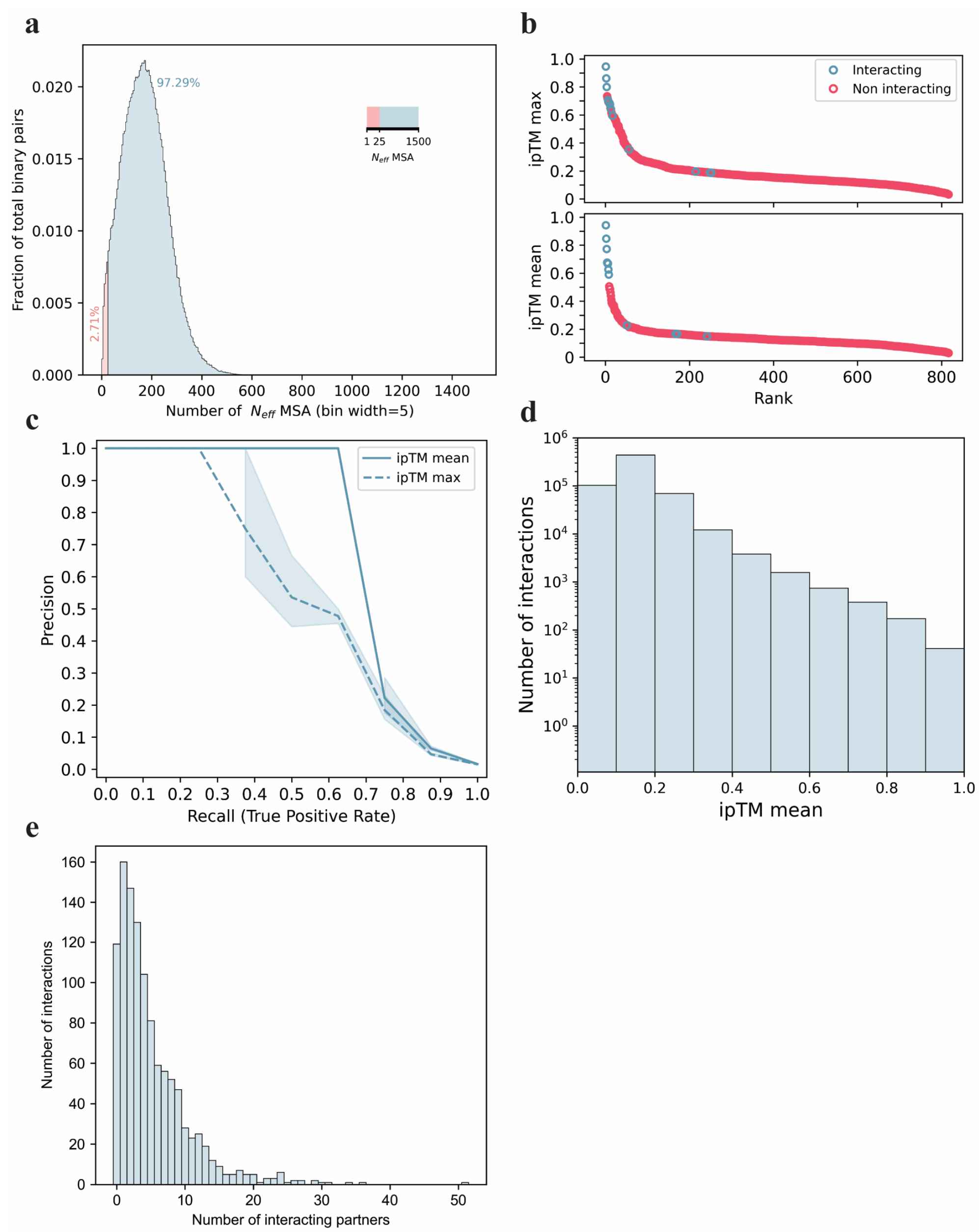

<u>Supplementary Fig. 2: Applying AlphaFold Multimer to mitochondrial proteome.</u>

a A histogram depicting the distribution of the number of effective (N_{eff}) MSA depth for all 630,003 protein pairs that were used in protein-protein interaction prediction. The histogram has a bin width of 5 N_{eff} MSAs. **b** Rank plot of experimentally validated interacting (blue) and non-interacting protein pairs (red) using maximum (top) and mean (bottom) ipTM scores for the mitochondrial copper pathway benchmark dataset. **c** Precision-recall curve of (**b**) at a 1:100 ratio of true:false pairs. **d** A histogram depicting the number of interactions for a given ipTM mean score. The histogram has a bin width of 0.1 ipTM mean score. **e** A histogram depicting the total number of interacting partners for all mitochondrial proteins. Source data are provided as a Source Data file.

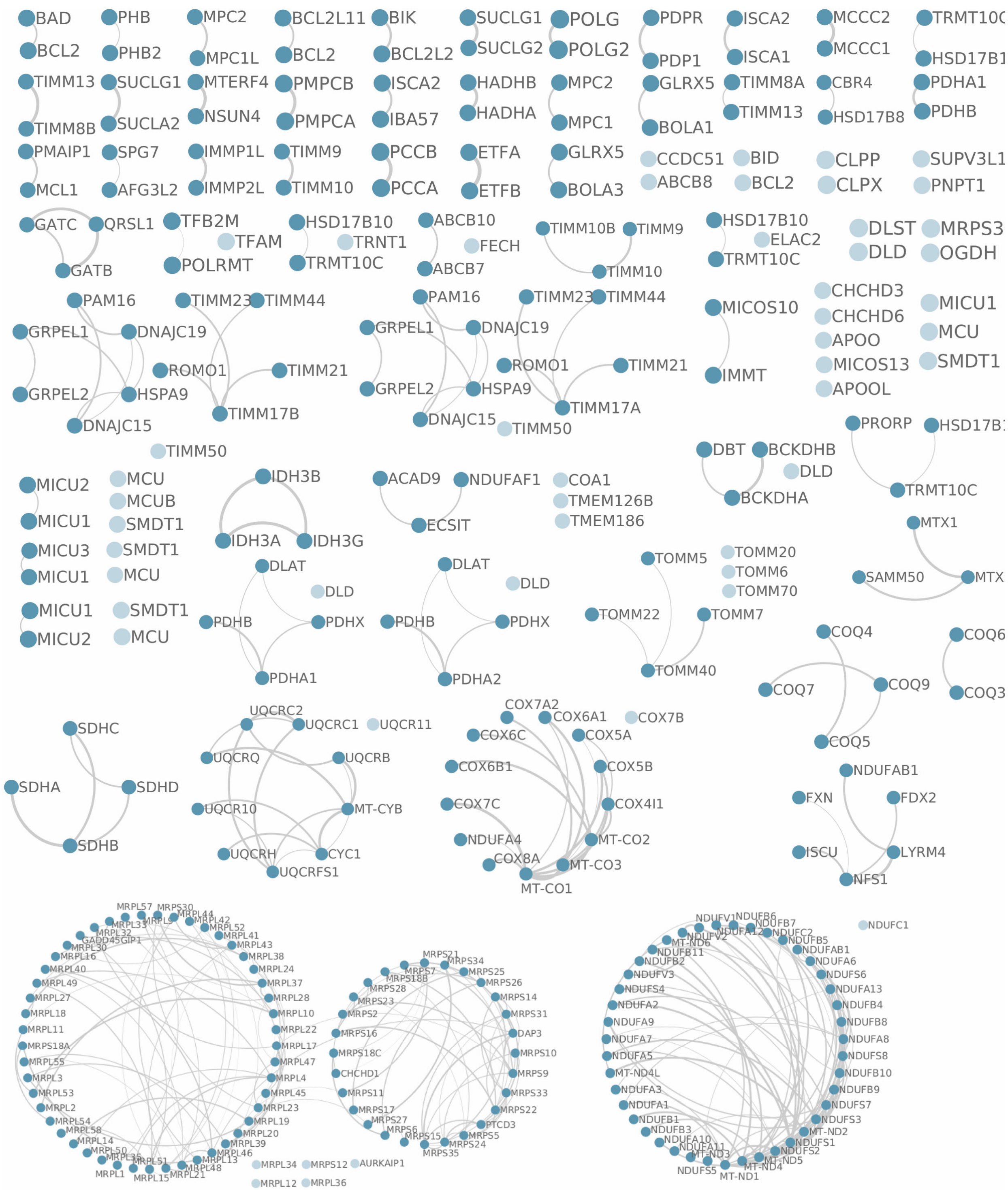

Supplementary Fig. 3: Mapping 2895 human mitochondrial hits to the complex portal database. Interaction networks for each of the 62 mitochondrial protein complexes listed in complex portal database.

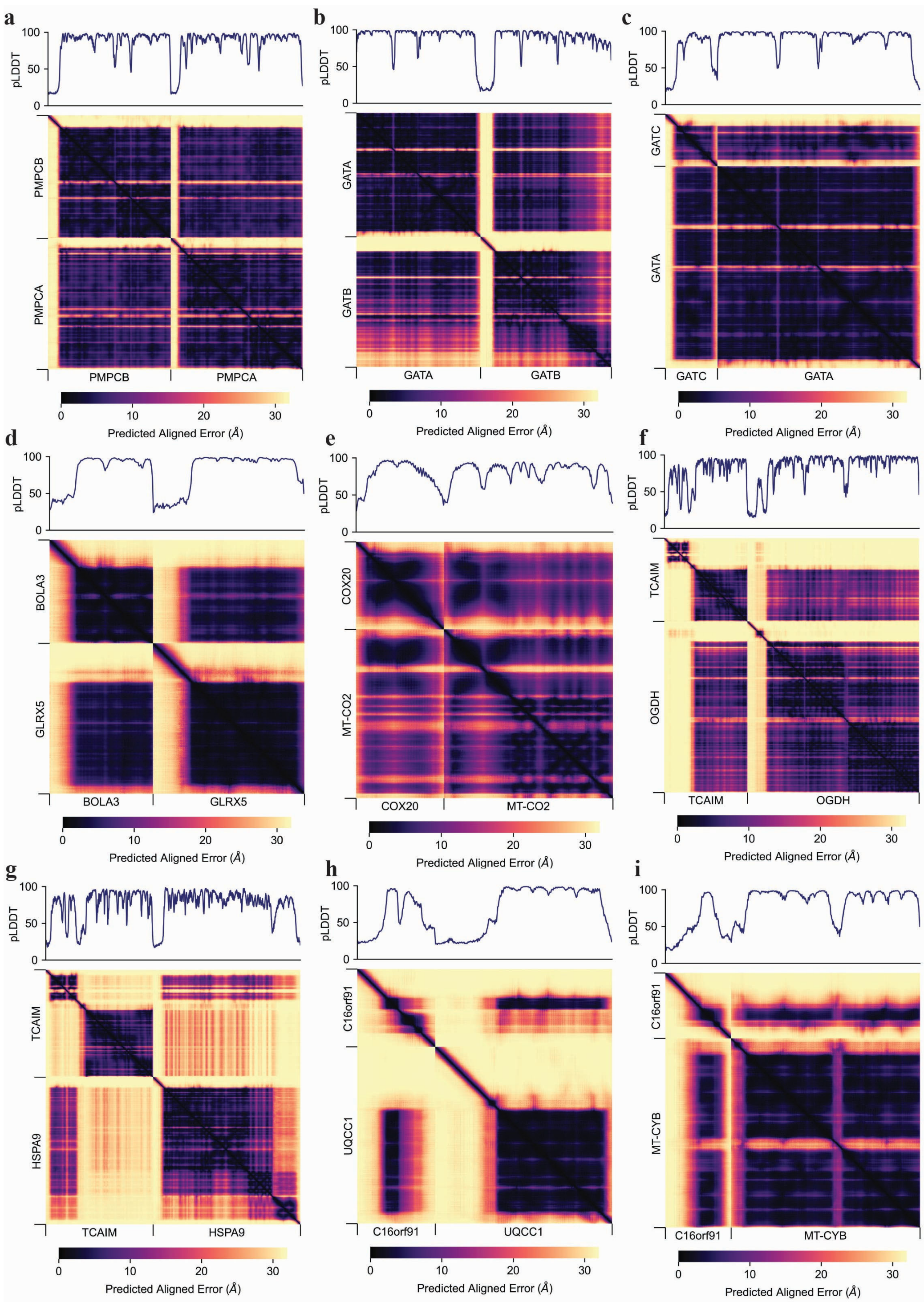

Supplementary Fig. 4: Confidence metrics of select mitochondrial interactions, related to Figure 1. Plots depicting the pLDDT metric (top) and the predicted aligned error metric (bottom) for **a** mitochondrial processing peptidase complex PMPCB-PMPCA, **b** glutamyl-t-RNA(Gln) amidotransferase GATA-GATB, and **c** GATC-GATA, **d** Fe-S cluster biogenesis factors BOLA3-GLRX5, **e** cytochrome *c* oxidase assembly intermediate COX20-COX2, **f** TCAIM-OGDH, **g** TCAIM-HSPA9, **h** C16ORF91-UQCC1, and **i** C16ORF91-MT-CYB interactions.

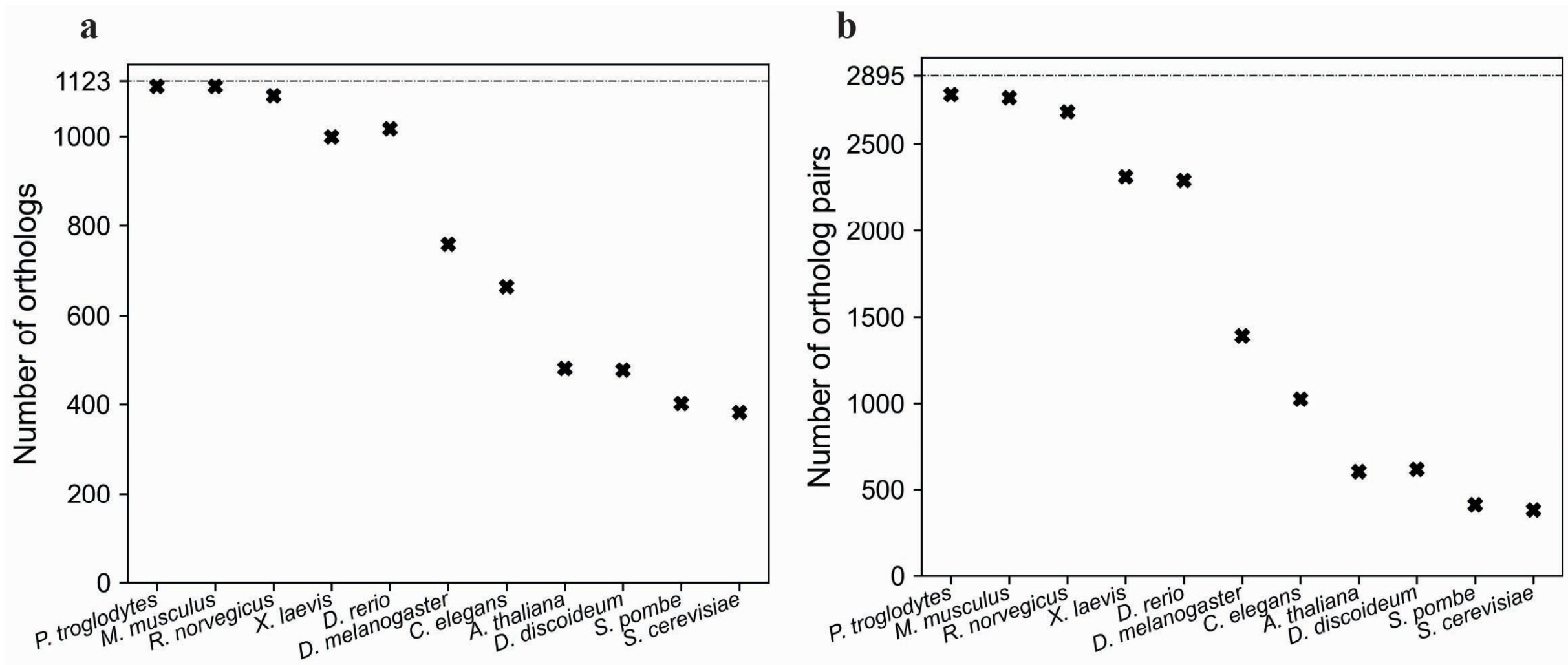

Supplementary Fig. 5: Finding homologs of human mitochondrial proteins in 11 other eukaryotes. **a** A plot depicting the number of homologs identified in the indicated species. The dotted line at the top denotes the number of human mitochondrial proteins used for this homolog identification. **b** A plot depicting the number of homologous protein pairs identified in the indicated species. The dotted line at the top denotes the number of human mitochondrial protein pairs used for this identification. Source data are provided as a Source Data file.

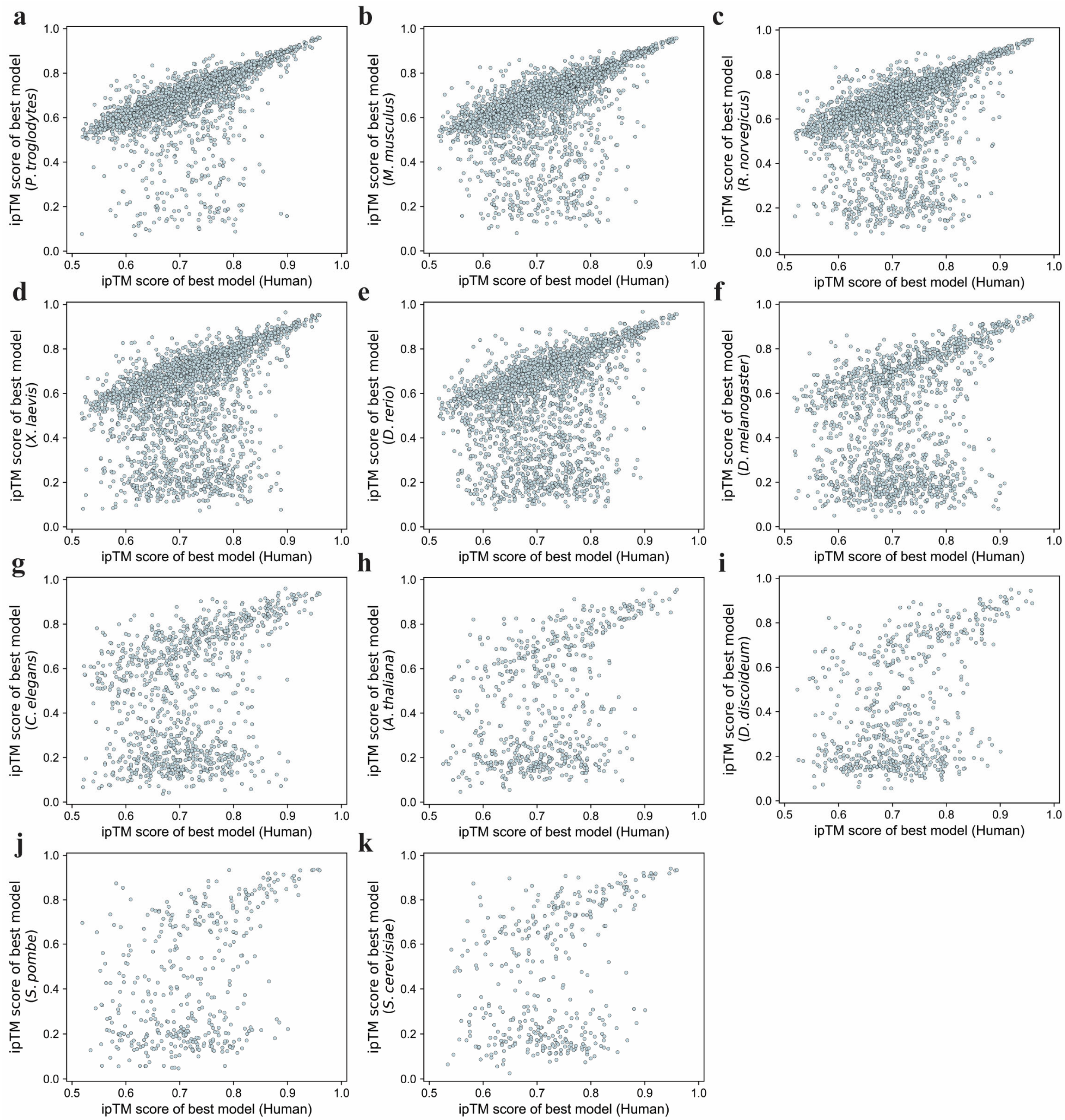

<u>Supplementary Fig. 6: Distribution of ipTM scores of the mitochondrial protein interactions predicted in 11 other eukaryotes.</u>

a-k A scatter plot depicting the ipTM score of the best model for each interaction predicted in the indicated species as a function of the best human ipTM score of the corresponding homologous pairs. Source data are provided as a Source Data file.

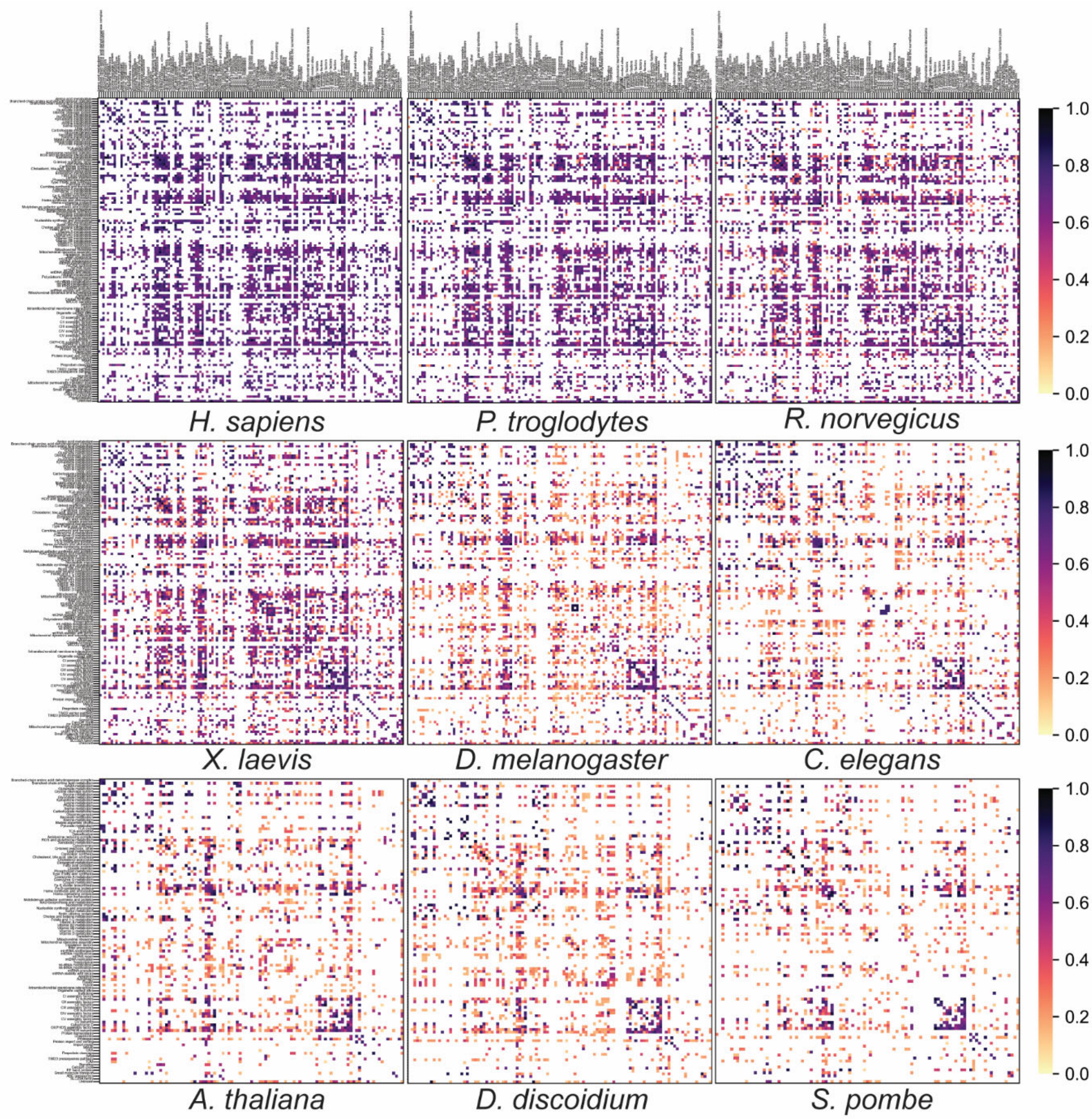

Supplementary Fig. 7: Evolutionary conservation of interactions in different mitochondrial pathways across eukaryotes.

Heatmap depicting the median ipTM scores of interactions between proteins of different mitochondrial pathways in the indicated organisms. Source data are provided as a Source Data file.

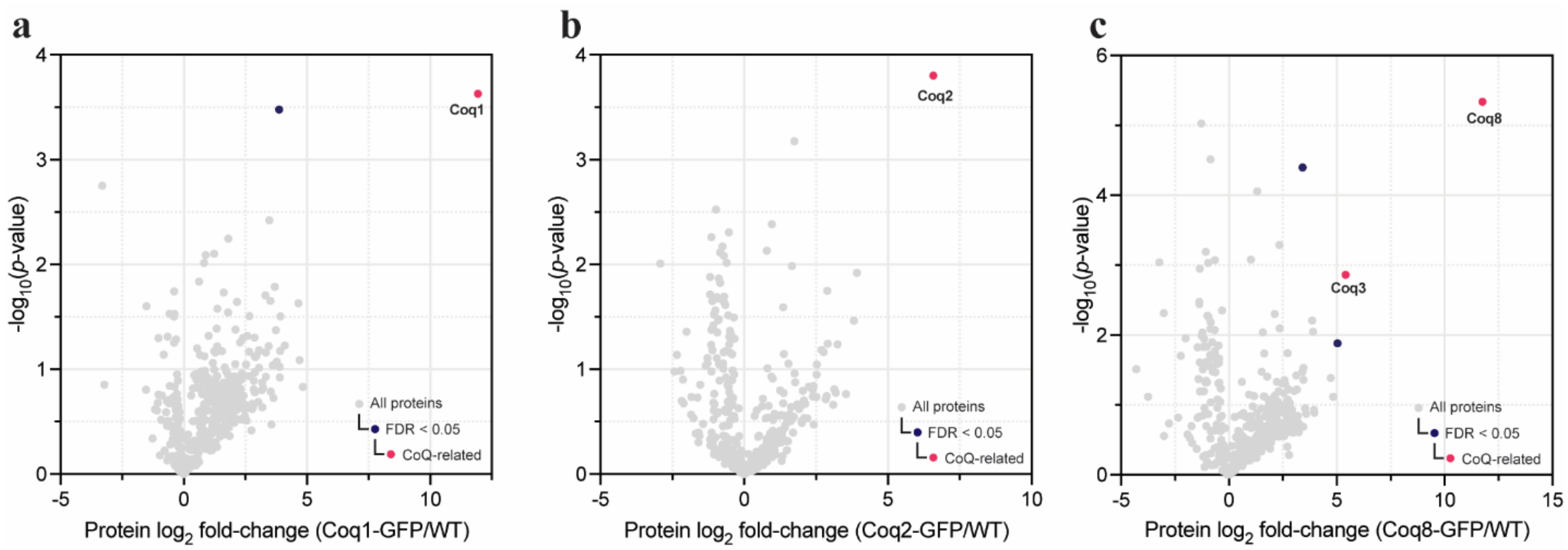

<u>Supplementary Fig. 8: Co-immunoprecipitation of yeast mitochondrial CoQ proteins.</u>

a-c Volcano plots obtained from immunoprecipitation of the indicated GFP-tagged proteins followed by mass spectrometry analysis. The data were normalized to immunoprecipitation performed in WT mitochondria. Statistical analysis was performed using two-sided Student's t-test. N=3 biological replicates.

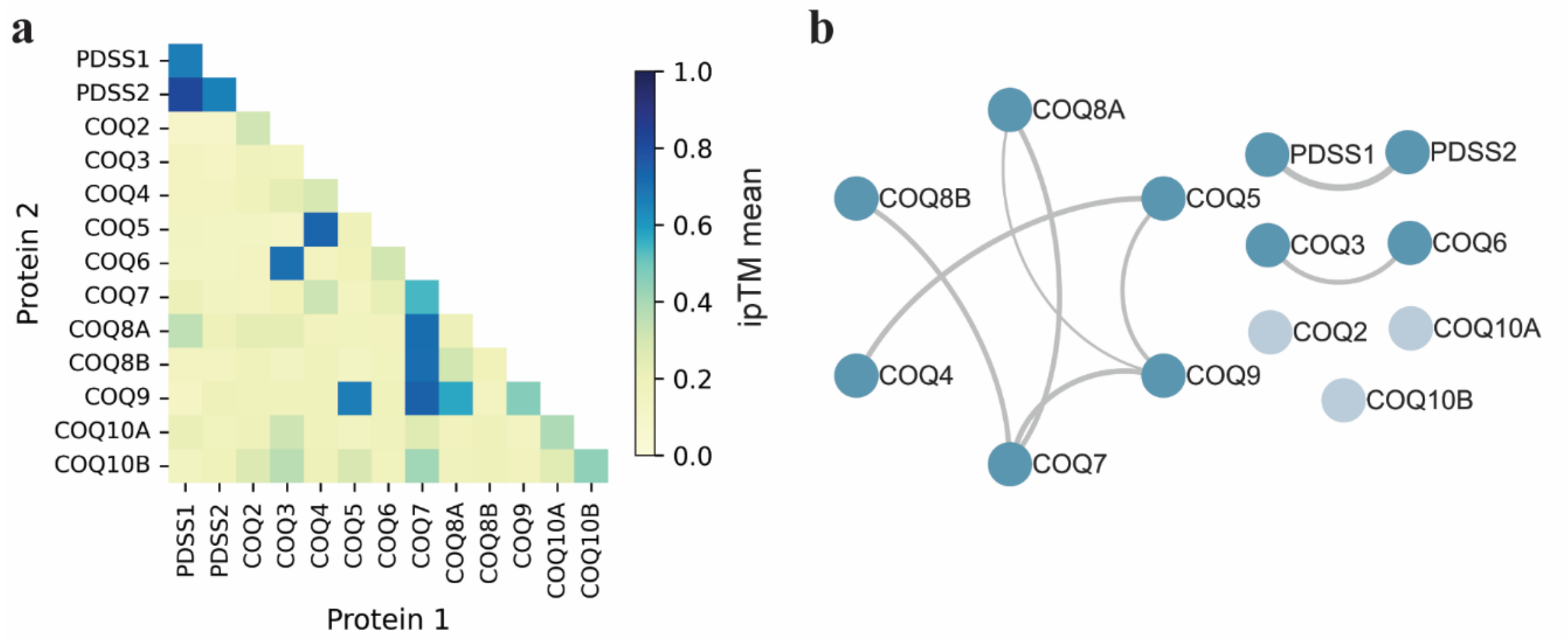

<u>Supplementary Fig. 9: Pairwise interaction predictions for human COQ proteins.</u>
a Heatmap depicting ipTM scores of pairwise interactions of human proteins involved in CoQ biosynthesis. **b** An interaction network of human COQ proteins based on the ipTM scores from panel (**a**). Source data are provided as a Source Data file.

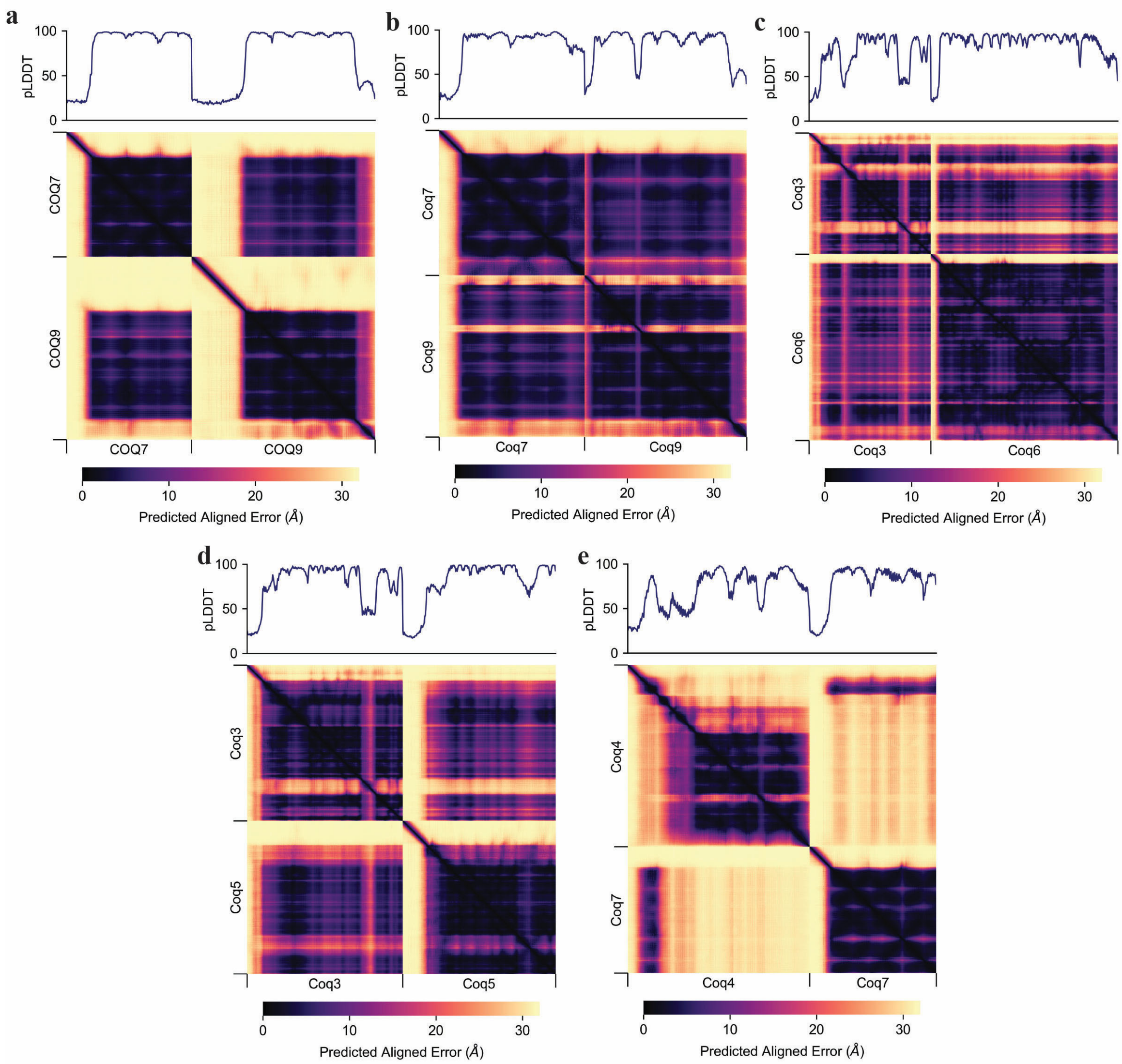

<u>Supplementary Fig. 10: Confidence metrics of select Coq protein interactions, related to Figure 3.</u>

Plots depicting the pLDDT metric (top) and the predicted aligned error metric (bottom) for **a** human COQ7-COQ9 interaction, and the yeast **b** Coq7-Coq9, **c** Coq3-Coq6, **d** Coq3-Coq5, **e** Coq4-Coq7 interactions.

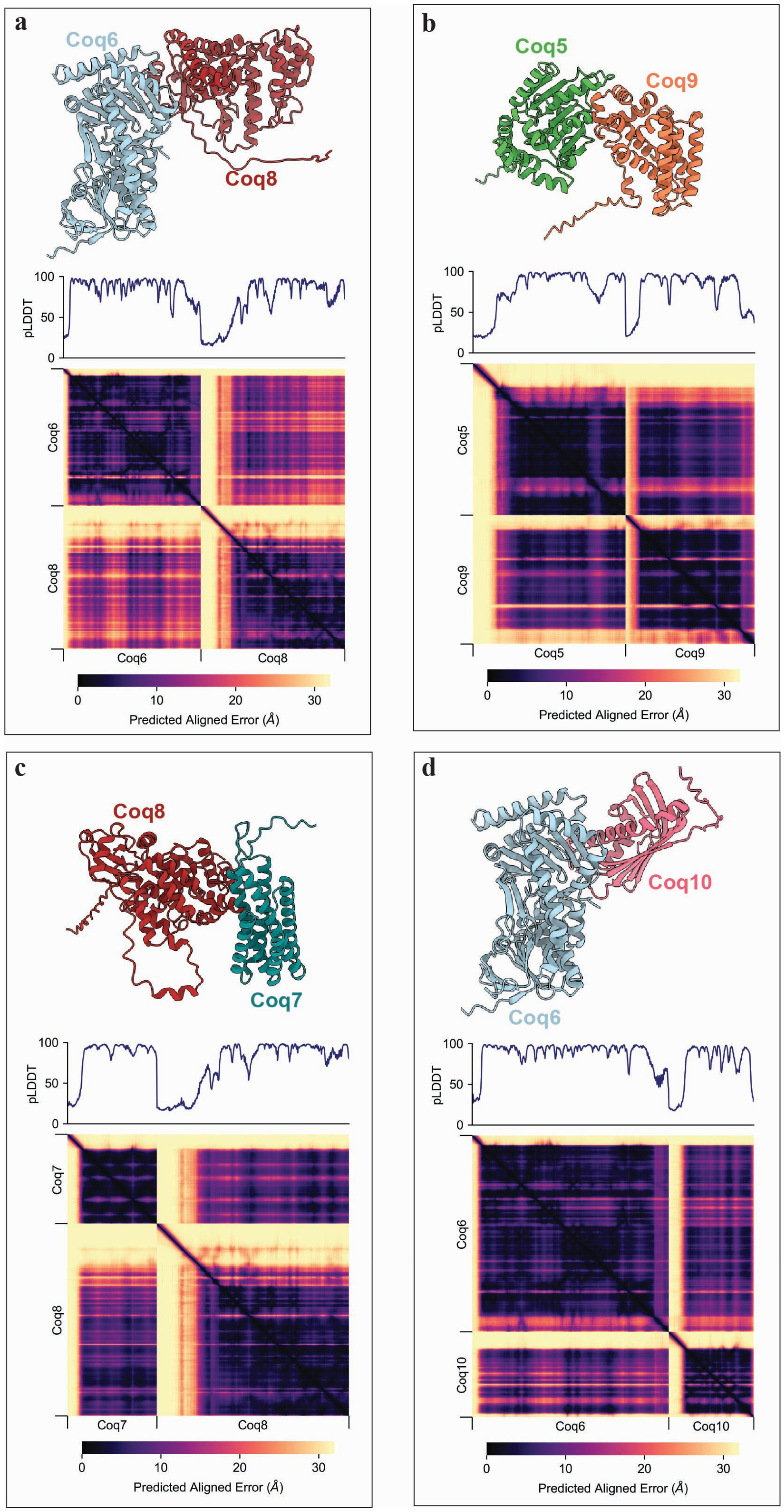

<u>Supplementary Fig. 11: Binary interactions of select yeast Coq proteins.</u>

Panels depicting the predicted structure (top), pLDDT metric (middle), and the predicted aligned error metric (bottom) for the yeast **a** Coq6-Coq8 **b** Coq5-Coq9, **c** Coq7-Coq8, **d** Coq6-Coq10 interactions.

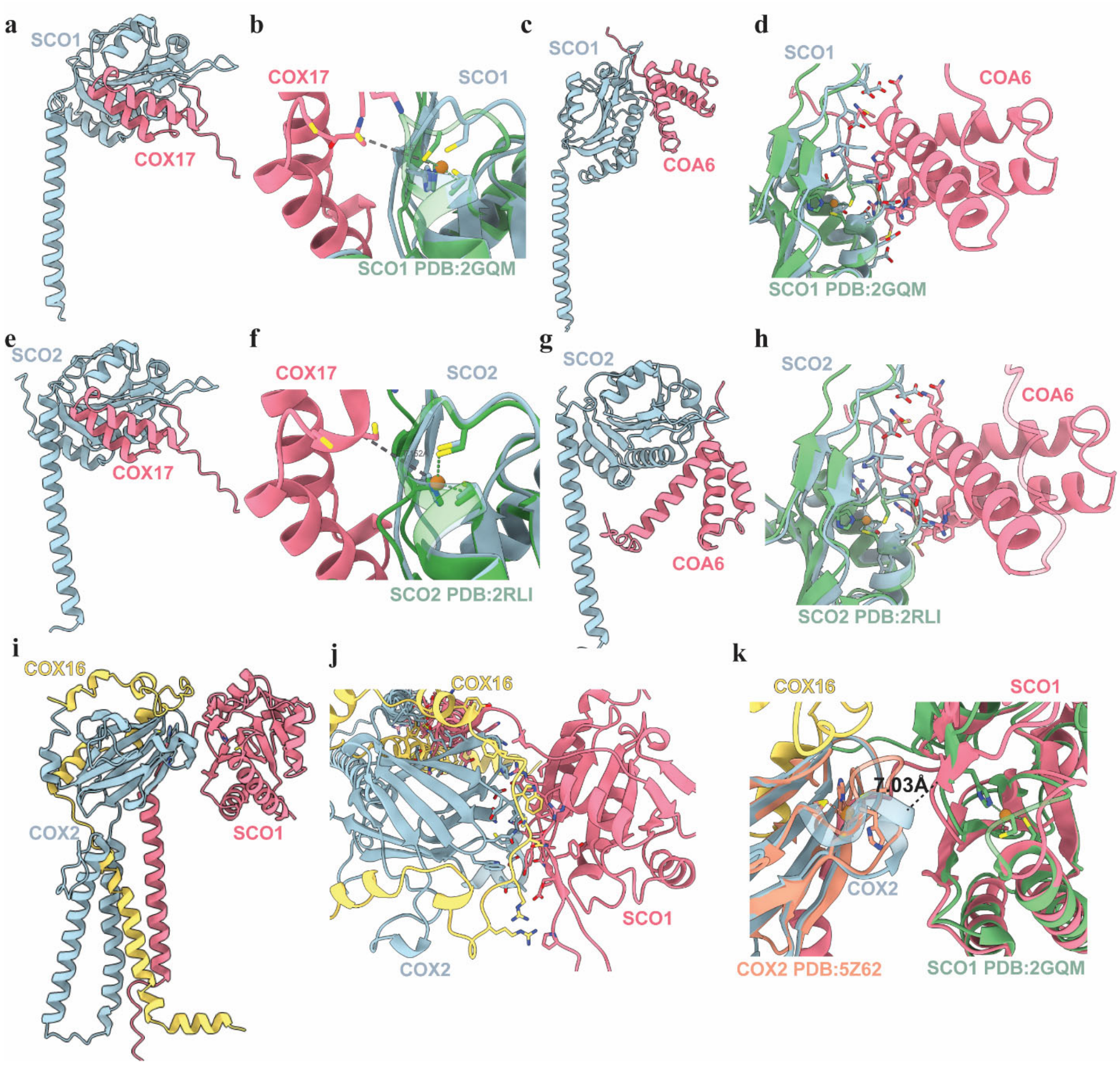

Supplementary Fig. 12: Predicted structures of protein-protein interactions in the assembly of Cu_A site of cytochrome *c* oxidase.

a Predicted structure of SCO1-COX17 complex. **b** Superposition of experimentally determined Cu-bound human SCO1 (depicted in green) on the SCO1-COX17 complex. **c** Predicted structure of SCO1-COA6 complex. **d** Superposition of experimentally determined Cu-bound human SCO1 (depicted in green) on the SCO1-COA6 complex. **e** Predicted structure of SCO2-COX17 complex. **f** Superposition of experimentally determined Cu-bound human SCO2 (depicted in green) on the SCO2-COX17 complex. **g** Predicted structure of SCO2-COA6 complex. **h** Superposition of experimentally determined Cu-bound human SCO2(depicted in green) on the SCO2-COA6 complex. **i** Predicted structure of COX16-COX2-SCO1 trimeric complex. **j** Analysis of the interface between COX16, COX2, and SCO1 reveals that a loop region of COX16 bridges the interaction between COX2 and SCO1. **k** Superposition of experimentally determined Cu-bound human COX2 and SCO1 structures on the COX16-COX2-SCO1 predicted complex. Please refer Supplementary Notes for a detailed description of this data.

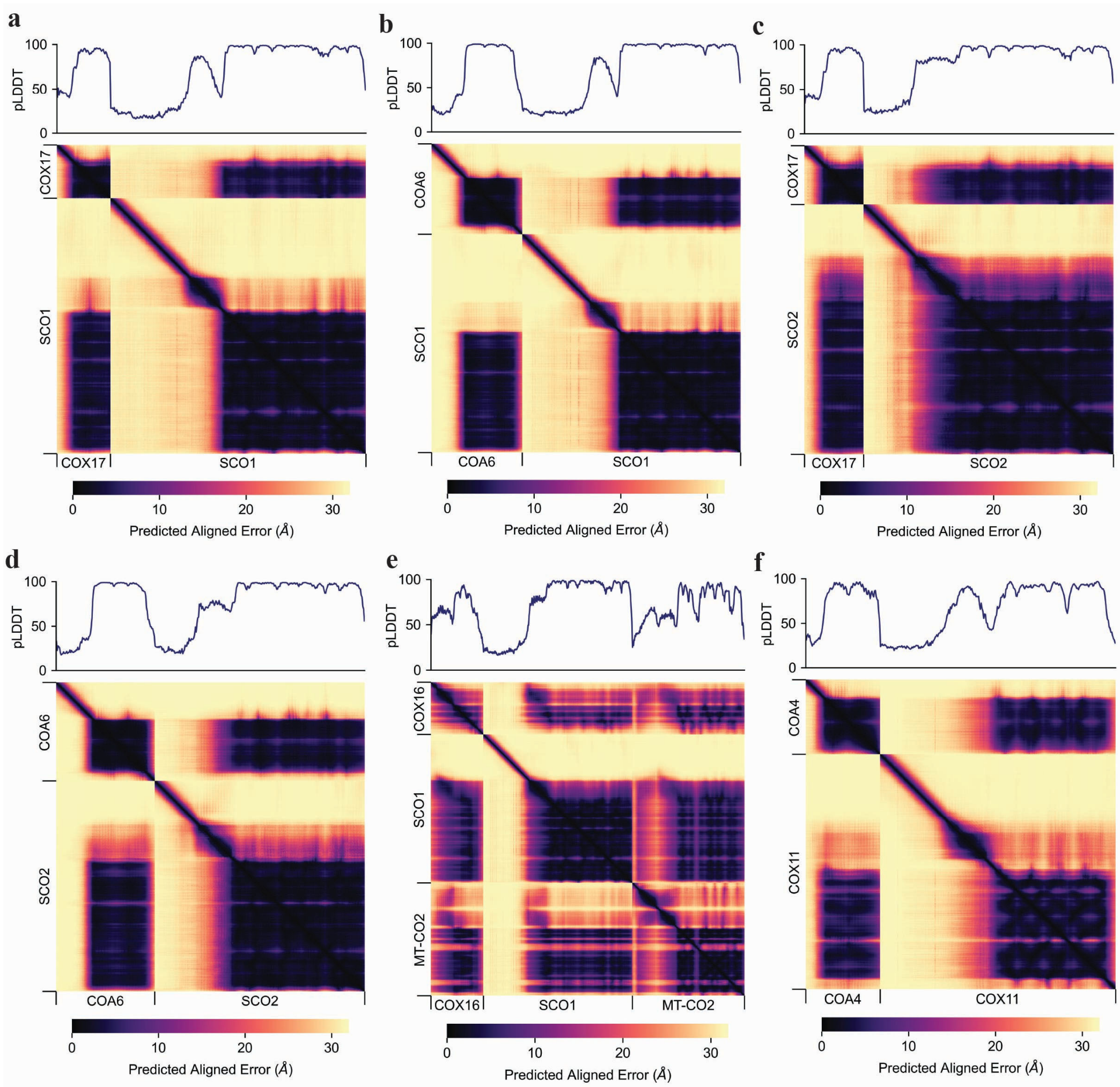

Supplementary Fig. 13: Confidence metrics of copper pathway protein interactions. Plots depicting the pLDDT metric (top) and the predicted aligned error metric (bottom) for human **a** COX17-SCO1, **b** COA6-SCO1, **c** COX17-SCO2, **d** COA6-SCO2, **e** COX16-SCO1-COX2, **f** COA4-COX11 interactions.

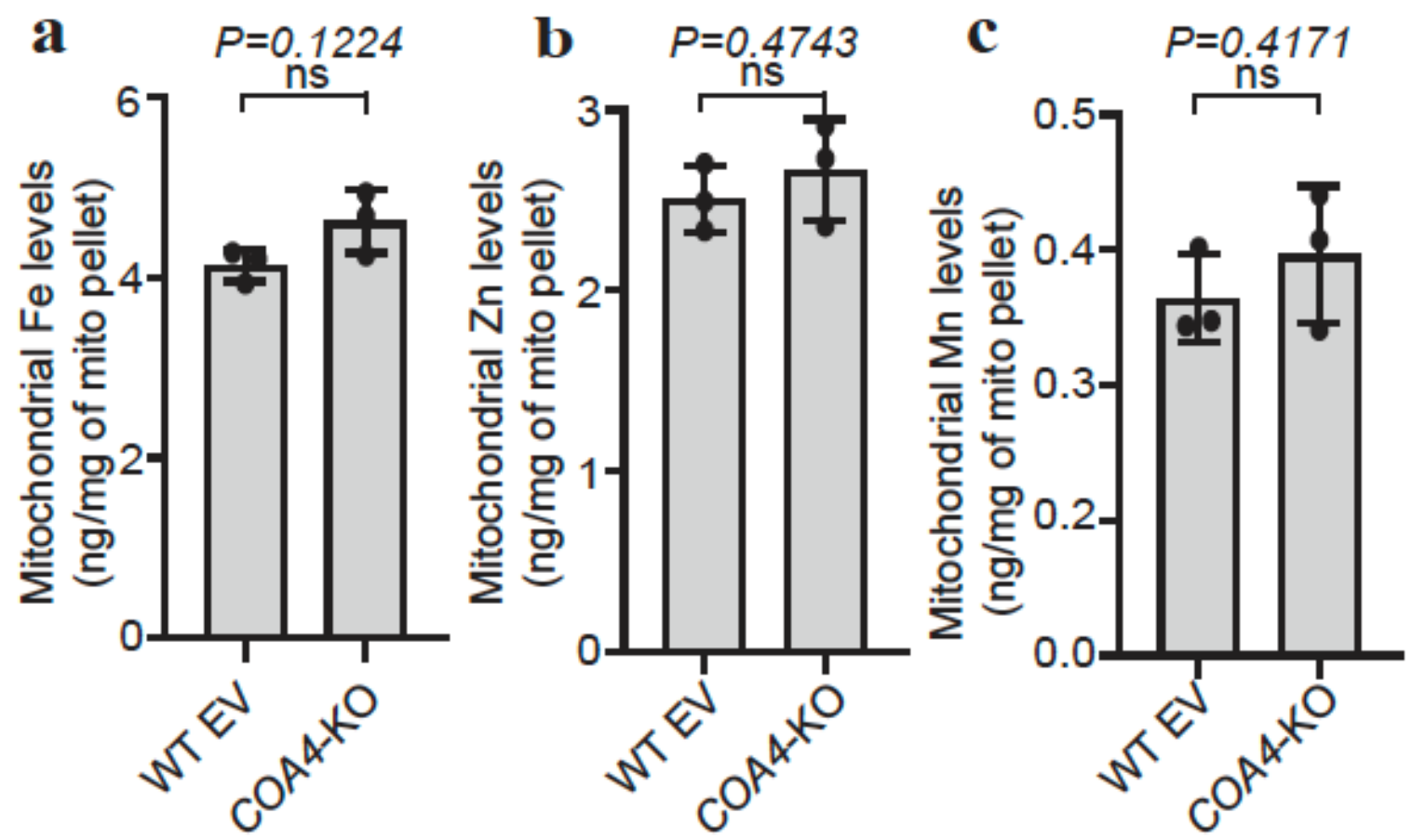

Supplementary Fig. 14: Mitochondrial metal measurements from MCH58 COA4-KO cells.

a Mitochondrial Fe **b** Zn and **c** Mn levels were measured in WT and COA4-KO mitochondria from MCH58 fibroblasts using Inductively coupled plasma - mass spectrometry. N=3 biological replicates from independent experiments. Data are presented as mean +/- SD. Statistical analyses were performed using unpaired two-tailed Student's t-test. Source data are provided as a Source Data file.

Supplementary Tables

Supplementary table 1: A list of 12 interactions that were curated for the mitochondrial test dataset. Related to Figures 1.

Protein pairs	Evidence for interaction	Reference	PDB ID for Structural evidence
COX17-COX11	Copper transfer using purified proteins	[1]	-
COX17-SCO1	Copper transfer using purified proteins	[1,2]	-
COX17-SCO2	Copper transfer using purified proteins	[2]	-
SCO1-COA6	Size exclusion chromatography of purified proteins	[3]	-
SCO2-COA6	Size exclusion chromatography of purified proteins	[3]	-
COX2-COA6	Disulfide reductase activity using purified proteins	[3]	-
COX2-COX6B	Cryo-electron microscopy	[4]	5z62
COX2-SCO1	Copper transfer using purified proteins	[5]	-
COX2-SCO2	Disulfide reductase activity using purified proteins	[5]	-
COX11-COX19	Co-elution of Cox19 upon purification of COX11 from isolated mitochondria	[6]	-
COX11-COX1	In vivo evidence for copper transfer from COX11 to COX1	[7]	-
COX1-COX2	Cryo-electron microscopy	[4]	5z62

Supplementary table 2: List of proteome identifiers used to obtain proteome sequences of the indicated organisms. Related to Figure 2.

**Proteome ID**	**Species name**
UP000005640	*H. sapiens*
UP000002277	*P. troglodytes*
UP000000589	*M. musculus*
UP000002494	*R. norvegicus*
UP000186698	*X. laevis*
UP000000437	*D. rerio*
UP000000803	*D. melanogaster*
UP000001940	*C. elegans*
UP000006548	*A. thaliana*
UP000002195	*D. discoidium*
UP000002485	*S. pombe*
UP000002311	*S. cerevisiae*

Supplementary Notes

Detemining the parameters that affect AFM success

To understand the underlying reason for the bimodal distribution of ipTM scores for the interacting pairs, we analyzed various parameters that could influence the prediction of protein-protein interactions by AFM. A key input that identifies co-evolutionary signals to help predict the interacting interface is the multiple sequence alignments (MSA), with a higher number of diverse sequences resulting in stronger signals. To determine if the MSA depth affects the ipTM scores, we first calculated the median of the number of effective MSAs at each residue position (N_{eff} MSA; Methods; Supplemental Methods) for all protein pairs of the recent PDB dataset. We find that AFM performs poorly for those interacting pairs that have less than 25 N_{eff} MSA sequences (Supplementary Fig. 1a,b). The interacting pairs with a N_{eff} MSA of more than 25 sequences perform well with median ipTM scores of more than 0.6, which steadily increases with increasing N_{eff} MSA (Supplementary Fig. 1a). On the other hand, the non-interacting protein pairs are not affected by the N_{eff} MSA since median ipTM score remains constantly low across different N_{eff} MSA bins (Supplementary Fig. 1a) suggesting that AFM as a classifier model would have a higher predictive value for evolutionarily conserved protein pairs with N_{eff} MSA depth of at least 25 diverse sequences. Indeed, the precision-recall curve plotted for different N_{eff} MSA depth shows that the AFM performs better at higher N_{eff} MSA depth (Supplementary Fig. 1b).

Next, we asked if the total number of interface residues in the experimental structure of the protein pairs affects the success rate of AFM. We find that the ipTM score increases steadily with increasing total interface residues and plateaus when the interaction has more than 40 total interface residues (Supplementary Fig. 1c). Importantly, each interface residue bin exhibits a bimodal distribution of ipTM scores, with protein pairs with low N_{eff} MSA depth exhibiting low ipTM scores as shown in red dots in Supplementary Fig. 1c, which suggests that N_{eff} MSA depth is the primary determinant of AFM success. To specifically investigate the effect of total interface residues, we plotted a precision-recall curve with only those pairs that have at least 25 N_{eff} MSA sequences (Supplementary Fig. 1d). We find that protein pairs with higher interface residues have a higher area under the curve . At 90% precision, the recall rate for protein pairs with >41 interface residues was at least 55%.

Since AFM was trained on intact protein complexes at their complete stoichiometry, we wondered if AFM could also successfully predict binary protein pairs derived from those complexes. We find that AFM works best for binary pairs derived from true heterodimers while the ipTM score drops steadily for binary pairs derived from higher-order complexes (Supplementary Fig. 1e). The median ipTM score of true dimeric interacting pairs is 0.86, pairs derived from small multimeric complexes of 3-9 subunits have a median ipTM score of 0.79, while those derived from large complexes of about 30 subunits or more drop to below 0.5 (Supplementary Fig. 1e). This is also reflected in the precision-recall curve, where heterodimers and binary pairs derived from small multimeric proteins outperform those derived from large multimeric complexes (Supplementary Fig. 1f). For instance, at 90% precision, AFM successfully predicts 75% of all heterodimers, 58% of binary pairs from oligomers of 3-9 stoichiometry, but only about 27% of binary pairs from oligomers containing more than 30 subunits (Supplementary

Fig. 1f). Taken together, these results suggests that AFM can be used as a classifier to predict protein-protein interactions.

Defining the copper delivery pathway to mitochondrial cytochrome *c* oxidase

Since the copper delivery process involves many protein-protein interactions that are mostly transient in nature, experimentally validated interactions involving the bona fide copper delivery pathway proteins have evaded structural studies. Even for a relatively better characterized copper delivery module to COX2, the structural basis of its function remains obscure. To address this, we analyzed the predicted structures of protein-protein interactions involved in COX2 assembly to determine if they are consistent with the experimental observations. Specific steps involved in copper metalation of COX2 can be broadly divided into the following steps – metalation of SCO1 by COX17, recruitment of SCO1 to COX2 by COX16 followed by transfer of copper from SCO1 to COX2[1,5,8] (Fig. 4a). AFM predicts an interaction between COX17 and the IMS-facing soluble domain of SCO1, which agrees with the localization and topology of these proteins (Supplementary Fig. 12a). The helices formed by the twin CX_9C domain of COX17 interact with the beta-hairpin of SCO1, placing the copper-coordinating loop of COX17 in close proximity to the copper-coordinating residues of SCO1 (Supplementary Fig. 12b). This proximity is consistent with the spatial arrangement required for efficient copper transfer, further supporting the validity of the predicted model. SCO1 has also been shown to interact with COA6[3,9,10], another CX_9C motif-containing protein, which facilitates copper binding by SCO1. AFM predicts an interaction between the c-terminal helix 3 of COA6 with the copper-coordinating residues of SCO1, which is consistent with our previously published nuclear magnetic resonance chemical shift perturbation data that implicated helix 3 of COA6 in its interaction with SCO1 (Supplementary Fig. 12c,d)[3]. SCO2, being the paralog of SCO1, also receives its copper from COX17 and interacts with COA6[2,3]. AFM also predicts an interaction between COX17-SCO2, and COA6-SCO2 (Fig. 4b,c), where the interface between the proteins is similar to that seen in COX17-SCO1 and COA6-SCO1 interactions, respectively (Supplementary Fig. 12e-h). Next, we asked if AFM could predict a trimer between COX2-COX16-SCO1 proteins based on a previous experimental study showing the recruitment role of COX16 for SCO1 for copper transfer to COX2[8]. AFM predicts an interaction between these proteins such that the IMS-facing region of COX16 bridges COX2 and SCO1 (Supplementary Fig. 12i,j). This interaction of COX16 with both the proteins positions the IMS facing copper-binding domains of SCO1 and COX2 in proximity to each other, providing a structural basis for how COX16 recruits SCO1 to COX2 for its metalation (Supplementary Fig. 12k). The confidence metrics including pLDDT and predicted aligned error scores for these interactions are provided in Supplementary Fig. 13a-e.

Supplementary Methods

Calculation of N_{eff} MSA depth

We implement the N_{eff} MSA depth calculation based on a previous study[11]. For a paired MSA M_{SxN} of shape SxN where S is the number of MSA sequences and N is the total number of residues of the query proteins, we first calculate an identity matrix SxS which is the sequence identity of each sequence in the MSA to every other sequence. We then apply an Iverson bracket operation at a sequence identity cutoff of 80%, which assigns a value of 1 if the sequence identity is greater than 80% else 0. In essence, the sum of each row should give us the number of sequences that have more than 80% sequence identity to the sequence in question i.e the cluster size and therefore the effective contribution or weight of that sequence in question is the inverse of the cluster size. The sum of such effective contribution or weight of each sequence is what is referred to as the effective (N_{eff}) MSA depth. We compute this N_{eff} MSA depth at each residue, by only considering sequences that does not have a gap at that residue resulting in what we refer to as the per-residue N_{eff} MSA depth. The median of this per-residue N_{eff} MSA depth is what we use for our analysis. The following is the Python implementation for this calculation:

```
#Code snippet 2
import os
import pickle as pkl
import json
import numpy as np
from datetime import datetime

def get_non_gap_length(msa):
  non_gap_length = np.count_nonzero(msa!=21, axis=1)
  return non_gap_length

def get_identity_matrix(msa):
  seqs = np.arange(0, len(msa))
  non_gap_length = get_non_gap_length(msa)

  identity_matrix = np.zeros((len(msa),len(msa)))
  for seq_i in seqs:
    non_gaps = (msa[seq_i] !=21)
    for seq_j in seqs:
      if seq_j >= seq_i:
        count = np.count_nonzero(msa[seq_i][non_gaps] == msa[seq_j][non_gaps])
        identity_matrix[seq_i, seq_j]=(count/((non_gap_length[seq_i]+non_gap_length[seq_j])/2))
        identity_matrix[seq_j, seq_i]=(count/((non_gap_length[seq_i]+non_gap_length[seq_j])/2))

  return np.asarray(identity_matrix)

def apply_iverson_bracket(identity_matrix, cutoff):
  return (identity_matrix>=cutoff).astype(int)

def calculate_weights(matrix_cutoff):
  weights = np.sum(matrix_cutoff, axis=1)
  weights = weights.flatten()
  inverse_weights = 1/weights
```

```
  return inverse_weights

def msa_depth(msa):
  ''' Counts number of non gap residues at each position '''
  num_of_non_gaps = np.count_nonzero(msa != 21, axis =0)
  msas_with_non_gaps = (msa != 21)
  return num_of_non_gaps, msas_with_non_gaps

def neff_msa_depth(msas_with_non_gaps,source_matrix_cutoff):
  msa_depth_neff = []
  length = np.arange(0,len(msa[0]))
  for res in length:
    non_zero_rows = msas_with_non_gaps[:,res]
    matrix_cutoff = source_matrix_cutoff[non_zero_rows]
    matrix_cutoff = matrix_cutoff[:, non_zero_rows]
    if matrix_cutoff.size ==0:
      msa_depth_neff.append(0)
      continue
    weights = calculate_weights(matrix_cutoff)
    neff = sum(weights)
    msa_depth_neff.append(neff)

  return msa_depth_neff

target_dir = "./neff_msa/paired_msa"

with open("protein_length_map.json", "r") as f:
  protein_length_map = json.load(f) # a dict containing the length of each protein monomers

target_files = os.listdir(target_dir)

msa_depth_map = {}
counter = 0
exceptions = []
initial_num = 0
final_num = len(target_files)

for protein in target_files:
  counter += 1
  print(f"{datetime.now()}: {counter} - starting calculations for protein {protein}")
  output_path = os.path.join(target_dir, protein, "paired_only_msa_depth.pkl")
  chain_features_path = os.path.join(target_dir,protein,f"{protein}_features.pkl")

  with open(chain_features_path, "rb") as f:
    features = pkl.load(f)
  msa = features['msa']
  protein_1_length = protein_length_map[protein.split("_")[0]]
  msa = np.asarray([row for row in msa if np.sum(row[protein_1_length:]!=21)!= 0])
  msa = np.asarray([row for row in msa if np.sum(row[0:protein_1_length]!=21)!= 0])

  identity_matrix = get_identity_matrix(msa)
  matrix_cutoff = apply_iverson_bracket(identity_matrix, 0.8)
  weights_total = calculate_weights(matrix_cutoff)
```

```
  neff_total = sum(weights_total)

  num_of_non_gaps,msas_with_non_gaps = msa_depth(msa)
  msa_depth_neff = neff_msa_depth(msas_with_non_gaps,matrix_cutoff)
  per_residue_median_neff = np.median(msa_depth_neff)
  msa_depth_map[protein] = per_residue_median_neff

  output = {'total_msas': len(msa),
       'total_msas_neff': neff_total,
       'non_gaps_per_residue':num_of_non_gaps,
       'non_gap_per_residue_neff': msa_depth_neff,
       'median_non_gap_per_residue_neff': per_residue_median_neff}

  with open(output_path, "wb") as f:
   pkl.dump(output,f,protocol=4)

 if exceptions:
  with open(f"exceptions.json", "w") as f:
   json.dump(exceptions,f,indent=4)
```

Generation of binary protein-protein interaction dataset from MitoCarta3.0

1136 human mitochondrial genes were downloaded from MitoCarta3.0 (https://www.broadinstitute.org/files/shared/metabolism/mitocarta/human.mitocarta3.0.html, last accessed 2025-01-09). Of these 1136 genes, 4 genes did not have an associated UniProt ID and so we manually mapped these 4 genes to a UniProt ID using the id-mapping tool of UniProt (https://www.uniprot.org/id-mapping, last accessed 2025-01-09). We successfully mapped the gene 154791 to UniProt ID Q96HJ9, and genes 548596 and 1159 both to UniProt ID P12532, while the gene ENSG00000230623 did not return a hit. Of these resulting 1134 proteins, we made a number of updates to the UniProt IDs:

- P30042 is an obsolete entry and was replaced with P0DPI2
- Q86UA3 was merged with Q9HB07 and so we used the latter ID
- the unreviewed entry B4DEF8 corresponding to the large mitochondrial ribosomal subunit mL45 was replaced with the reviewed ID Q9BRJ2
- the unreviewed entry B4DJ38 was removed since its closest match to a reviewed ID O75127 was already present in our list.
- removed five proteins – P07203, P36969, Q9NNW7, Q16881, Q9BVL4 – that contain a non-canonical aminoacid.
- removed five more proteins – Q13085, O00763, Q5THJ4, P49327, O75417 – that were more than 2000 amino acids in length.

This results in a total of 1123 proteins that were used to generate 630003 heteromeric binary combinations.

Identification of orthologs in other eukaryotic species

We use MMseqs2[12] to implement a reciprocal best hit search that identifies orthologs in other species. First we downloaded the fasta sequence of the target proteomes from UniProt. The

proteome identifiers of these species are listed in Supplementary table 2. We then generate an MMseqs database using the following command:

```
mmseqs createdb <path_to_fasta> <path_to_output_database>  &&
mmseqs createindex <path_to_output_database> ./tmp
```

We then perform sequence alignment of all human proteins to target species and vice versa using MMseqs2 as shown below:

```
mmseqs search <path_to_query_db> <path_to_target_db> <path_to_output_db> ./tmp --num-iterations 3 -
-db-load-mode 0 -a -s 8 -e 0.1
mmseqs convertalis <path_to_query_db> <path_to_target_db> <path_to_output_db>
<path_to_output_tab_file> --format-mode 0
```

For each human protein of interest, we obtain the best hit (lowest e-value) in the target species, and check if the best hit of this target protein (i.e., the reciprocal best hit) maps back to our protein of interest. As an illustration, the following is what we used to identify yeast orthologs of human mitocarta proteins in a single Python script.

```
 #Code snippet 3
 import json
 import os
 import numpy as np
 import subprocess

 proteome_1 = "human"
 proteome_2 = "yeast"

 query_db = f"./database/{proteome_1}_proteome_DB"
 target_db= f"./database/{proteome_2}_proteome_DB"
 output_dir = f"./ortholog_alignment/{proteome_2}_ortholog_alignment"
 if not os.path.exists(output_dir):
   os.makedirs(output_dir)
 output_db= os.path.join(output_dir, f"{proteome_1}_to_{proteome_2}_alignment")
 output_tab_file=
f"./ortholog_alignment/{proteome_2}_ortholog_alignment/{proteome_1}_to_{proteome_2}_alignment.tab"

 mmseqs_alignment_command = f"mmseqs search {query_db} {target_db} {output_db} ./tmp --num-
iterations 3 --db-load-mode 0 -a -s 8 -e 0.1"
 mmseqs_convertalis_command = f"mmseqs convertalis {query_db} {target_db} {output_db}
{output_tab_file} --format-mode 0"
 mmseqs_alignment = subprocess.Popen([mmseqs_alignment_command], shell=True)
 mmseqs_alignment.communicate()
 mmseqs_alignment = subprocess.Popen([mmseqs_convertalis_command], shell=True)
 mmseqs_alignment.communicate()

 reciprocal_output_db= os.path.join(output_dir, f"{proteome_2}_to_{proteome_1}_alignment")
 reciprocal_output_tab_file=
f"./ortholog_alignment/{proteome_2}_ortholog_alignment/{proteome_2}_to_{proteome_1}_alignment.tab"

 mmseqs_alignment_command = f"mmseqs search {target_db} {query_db} {reciprocal_output_db} ./tmp --
num-iterations 3 --db-load-mode 0 -a -s 8 -e 0.1"
```

```
 mmseqs_convertalis_command = f"mmseqs convertalis {target_db} {query_db} {reciprocal_output_db}
{reciprocal_output_tab_file} --format-mode 0"
 mmseqs_alignment = subprocess.Popen([mmseqs_alignment_command], shell=True)
 mmseqs_alignment.communicate()
 mmseqs_alignment = subprocess.Popen([mmseqs_convertalis_command], shell=True)
 mmseqs_alignment.communicate()

 with open("./uniprot_geneid.json", "r") as f:
   mitocarta = json.load(f)
 mitocarta = list(mitocarta.keys())

 with open(output_tab_file, "r") as f:
   alignment_1 = f.readlines()

 with open(reciprocal_output_tab_file, "r") as f:
   alignment_2 = f.readlines()

 def parse_mmseqs2_aln(line):
   query = line.split("\t")[0]
   target = line.split("\t")[1] # remove version number for ncbi dataset
   seq_identity = line.split("\t")[2]
   alignment_length = line.split("\t")[3]
   num_mismatches = line.split("\t")[4]
   num_gap_openings = line.split("\t")[5]
   query_domain_start = line.split("\t")[6]
   query_domain_stop = line.split("\t")[7]
   target_domain_start = line.split("\t")[8]
   target_domain_stop = line.split("\t")[9]
   e_value = float(line.split("\t")[10])
   bit_score = line.split("\t")[11]
   return query, target, seq_identity, e_value

 hit_map_1 = {}
 for each_hit in alignment_1:
   query, target, seq_identity, e_value = parse_mmseqs2_aln(each_hit)
   if query not in hit_map_1:
     hit_map_1[query] = {}
   hit_map_1[query][target] = e_value

 best_hit_map = {}

 for each_hit in hit_map_1:
   hits = dict(sorted(hit_map_1[each_hit].items(), key=lambda item:item[1], reverse=False))
   best_hit = list(hits.keys())[0]
   best_hit_map[each_hit] = best_hit


 hit_map_2 = {}
 for each_hit in alignment_2:
   query, target, seq_identity, e_value = parse_mmseqs2_aln(each_hit)
   if query not in hit_map_2:
     hit_map_2[query] = {}
   hit_map_2[query][target] = e_value
```

```
reciprocal_best_hit_map = {}
for each_hit in hit_map_2:
  hits = dict(sorted(hit_map_2[each_hit].items(), key=lambda item:item[1], reverse=False))
  best_hit = list(hits.keys())[0]
  reciprocal_best_hit_map[each_hit] = best_hit


ortholog_map = {}
for each_hit in best_hit_map:
  if best_hit_map[each_hit] not in reciprocal_best_hit_map:
    continue
  if each_hit == reciprocal_best_hit_map[best_hit_map[each_hit]]:
    ortholog_map[each_hit] = best_hit_map[each_hit]

ortholog_map_mito = {key:value for key,value in ortholog_map.items() if key in mitocarta}
with open(f"./human_to_ortholog_map/{proteome_1}_to_{proteome_2}_homolog.json", "w") as f:
  json.dump(ortholog_map_mito, f, indent=4)

with open(f"./ortholog_hits_list/{proteome_2}_mitocarta_homolog.json", "w") as f:
  json.dump(list(ortholog_map_mito.values()), f, indent=4)

ortholog_map_mito = [f"{key}\t{value}\n" for key,value in ortholog_map_mito.items()]
with open(f"./ortholog_hits_list/{proteome_1}_to_{proteome_2}_homolog.tsv", "w") as f:
  f.writelines(ortholog_map_mito)
```

Calculation of total interface residues

We implement the NeighborSearch module of BioPython to determine the interface residues between two chains in a cif structure file as shown below:

```
#Code snippet 4
import os
import json
import pickle as pkl
from Bio.PDB import PDBParser, NeighborSearch, MMCIFParser
from Bio.PDB.Selection import unfold_entities
from Bio.PDB.PDBIO import Select
from Bio.PDB.mmcifio import MMCIFIO
from Bio.PDB.Polypeptide import is_aa
import numpy as np
import warnings
warnings.filterwarnings('ignore')

structure_path = "...../database/pdb_bioassembly_mmcif/all"
cif_parser = MMCIFParser(QUIET=1)
p = PDBParser(QUIET=1)
io = MMCIFIO()

dataset =[.....]] # a list of cif files

class Select_Protein(Select):
```

```
  def accept_residue(self, residue):
    return 1 if is_aa(residue)==True else 0

class NonHetSelect(Select):
  def accept_residue(self, residue):
    return 1 if residue.id[0] == " " else 0

def convert_to_list(data):
  if type(data)== list:
    return data
  else:
    return [data]

hetero_contact_map_all = {}

counter = 1
for each_pair in dataset:
  print(f"{counter} of {len(dataset)}: {each_pair}")
  counter += 1
  pdb_id = each_pair.split(".")[0]
  if pdb_id in hetero_contact_map_all:
    continue
  pdb_path = os.path.join(structure_path, f"{pdb_id}.cif")
  struct = cif_parser.get_structure(f"{pdb_id}", pdb_path)
  io.set_structure(struct)
  io.save("input_tmp.cif", NonHetSelect())
  struct = cif_parser.get_structure(f"{pdb_id}", "input_tmp.cif")

  all_chains = [chain.get_id() for chain in struct.get_chains()]
  all_chain_pairs = []
  n = np.arange(0,len(all_chains))
  for chain_1 in n:
    for chain_2 in n[chain_1+1:]:
      all_chain_pairs.append([all_chains[chain_1],all_chains[chain_2]])

  hetero_contact_map = {frozenset(chain):{chain[0]:[], chain[1]:[]} for chain in all_chain_pairs}
  all_atoms = unfold_entities(struct, 'A')
  ns_all = NeighborSearch(all_atoms)
  all_chain_contacts = ns_all.search_all(4, level='A')
  #get the chain id (parent of parent) the residue id (parent) and atom id
  all_contacts = [[{'chain':x[0].get_parent().get_parent().get_id(), 'residue':x[0].get_parent().get_id(),
'atom': x[0].get_id()}, {'chain':x[1].get_parent().get_parent().get_id(), 'residue':x[1].get_parent().get_id(),
'atom':x[1].get_id()}] for x in all_chain_contacts]
  all_contacts = [x for x in all_contacts if x[0]['chain'] in all_chains and x[1]['chain'] in all_chains]
  hetero_contacts = [x for x in all_contacts if x[0]['chain'] != x[1]['chain']]
  for contact in hetero_contacts:
    if contact[0]['residue'][1] not in hetero_contact_map[frozenset([contact[0]['chain'],
contact[1]['chain']])][contact[0]['chain']]:
      hetero_contact_map[frozenset([contact[0]['chain'],
contact[1]['chain']])][contact[0]['chain']].append(contact[0]['residue'][1])
    if contact[1]['residue'][1] not in hetero_contact_map[frozenset([contact[0]['chain'],
contact[1]['chain']])][contact[1]['chain']]:
```

```
      hetero_contact_map[frozenset([contact[0]['chain'],
contact[1]['chain']])][contact[1]['chain']].append(contact[1]['residue'][1])
    hetero_contact_map_all[pdb_id] = hetero_contact_map

  with open("interacting_residues.pkl", "wb") as f:
    pkl.dump(hetero_contact_map_all, f, protocol=4)
```

Calculation of Baseline score

We used the following python script to calculate the baseline score for each protein:

```
 #Code snippet 5
import pandas as pd
import numpy as np
from scipy.stats import norm
import json

dataset = pd.read_csv("alphafold_results.tsv", sep="\t", index_col=0)

data = {}
for idx, row in dataset.iterrows():
    for uniprot in [row["Uniprot_1"], row["Uniprot_2"]]:
        if uniprot not in data:
            data[uniprot] = []
        data[uniprot].append(row["iptm_mean"])

bulk_means = {}
for protein, scores in data.items():
    scores = np.array(scores)
    bulk = scores[scores < 0.5]
    bulk_means[protein] = np.mean(bulk)

means = np.array(list(bulk_means.values()))
baseline_Z = (means - means.mean()) / means.std()
baseline_scores = dict(sorted(zip(bulk_means.keys(), baseline_Z),
                    key=lambda x: x[1], reverse=True))

with open("baseline_scores.json", "w") as f:
 json.dump(baseline_scores,f,indent=4)
```

Supplementary References

1. Horng, Y. C., Cobine, P. A., Maxfield, A. B., Carr, H. S. & Winge, D. R. Specific copper transfer from the Cox17 metallochaperone to both Sco1 and Cox11 in the assembly of yeast cytochrome *c* oxidase. *J Biol Chem* **279**, 35334-35340 (2004).
2. Banci, L. *et al.* Mitochondrial copper(I) transfer from Cox17 to Sco1 is coupled to electron transfer. *Proc Natl Acad Sci U S A* **105**, 6803-6808 (2008).
3. Soma, S. *et al.* COA6 Is Structurally Tuned to Function as a Thiol-Disulfide Oxidoreductase in Copper Delivery to Mitochondrial Cytochrome *c* Oxidase. *Cell Rep* **29**, 4114-4126 e4115 (2019).
4. Zong, S. *et al.* Structure of the intact 14-subunit human cytochrome *c* oxidase. *Cell Res* **28**, 1026-1034 (2018).
5. Morgada, M. N. *et al.* Loop recognition and copper-mediated disulfide reduction underpin metal site assembly of CuA in human cytochrome oxidase. *Proc Natl Acad Sci U S A* **112**, 11771-11776 (2015).
6. Bode, M. *et al.* Redox-regulated dynamic interplay between Cox19 and the copper-binding protein Cox11 in the intermembrane space of mitochondria facilitates biogenesis of cytochrome c oxidase. *Mol Biol Cell* **26**, 2385-2401 (2015).
7. Hiser, L., Di Valentin, M., Hamer, A.G. & Hosler, J.P. Cox11p is required for stable formation of the Cu(B) and magnesium centers of cytochrome *c* oxidase. *J Biol Chem* **275**, 619-623 (2000).
8. Aich, A. *et al.* COX16 promotes COX2 metallation and assembly during respiratory complex IV biogenesis. *Elife* **7** (2018).
9. Ghosh, A. *et al.* Mitochondrial disease genes COA6, COX6B and SCO2 have overlapping roles in COX2 biogenesis. *Hum Mol Genet* **25**, 660-671 (2016).
10. Stroud, D. A. *et al.* COA6 is a mitochondrial complex IV assembly factor critical for biogenesis of mtDNA-encoded COX2. *Hum Mol Genet* **24**, 5404-5415 (2015).
11. Zhang, C., Zheng, W., Mortuza, S. M., Li, Y. & Zhang, Y. DeepMSA: constructing deep multiple sequence alignment to improve contact prediction and fold-recognition for distant-homology proteins. *Bioinformatics* **36**, 2105-2112 (2020).
12. Steinegger, M. & Soding, J. MMseqs2 enables sensitive protein sequence searching for the analysis of massive data sets. *Nat Biotechnol* **35**, 1026-1028 (2017).
