# Supplementary material for: The predicted interactome of the human mitochondrial proteome
Source: Nat Commun. 2026 Aug 25;17:10162. doi: 10.1038/s41467-026-77112-z (PMC13601606; doi:10.1038/s41467-026-77112-z)
Supplement: Supplementary file 2 [file 41467_2026_77112_MOESM2_ESM.pdf]

Description of Additional Supplementary Files

File name: Supplementary Data 1

Description: The file contains the ipTM scores of the 2895 MitoMatch hits of the human mitochondrial protein pairs generated using AlphaFold Multimer.

File name: Supplementary Data 2

Description: The file contains validation and additional confidence scores for 2895 MitoMatch hits.

File name: Supplementary Data 3

Description: ipTM scores of the MitoMatch hits for 11 eukaryotic organisms.
