# Supplementary material for: The predicted interactome of the human mitochondrial proteome
Source: Nat Commun. 2026 Aug 25;17:10162. doi: 10.1038/s41467-026-77112-z (PMC13601606; doi:10.1038/s41467-026-77112-z)
Supplement: Supplementary file 3 [file 41467_2026_77112_MOESM6_ESM.pdf]

nature portfolio

Corresponding author(s): Abhinav B. Swaminathan; Vishal M. Gohil

Last updated by author(s): Jul 26, 2026

Reporting Summary

Nature Portfolio wishes to improve the reproducibility of the work that we publish. This form provides structure for consistency and transparency in reporting. For further information on Nature Portfolio policies, see our Editorial Policies and the Editorial Policy Checklist.

Statistics

For all statistical analyses, confirm that the following items are present in the figure legend, table legend, main text, or Methods section.

n/a	Confirmed	
[ ]	[x]	The exact sample size (*n*) for each experimental group/condition, given as a discrete number and unit of measurement
[ ]	[x]	A statement on whether measurements were taken from distinct samples or whether the same sample was measured repeatedly
[ ]	[x]	The statistical test(s) used AND whether they are one- or two-sided<br>*Only common tests should be described solely by name; describe more complex techniques in the Methods section.*
[ ]	[x]	A description of all covariates tested
[ ]	[x]	A description of any assumptions or corrections, such as tests of normality and adjustment for multiple comparisons
[ ]	[x]	A full description of the statistical parameters including central tendency (e.g. means) or other basic estimates (e.g. regression coefficient) AND variation (e.g. standard deviation) or associated estimates of uncertainty (e.g. confidence intervals)
[ ]	[x]	For null hypothesis testing, the test statistic (e.g. *F*, *t*, *r*) with confidence intervals, effect sizes, degrees of freedom and *P* value noted<br>*Give P values as exact values whenever suitable.*
[x]	[ ]	For Bayesian analysis, information on the choice of priors and Markov chain Monte Carlo settings
[x]	[ ]	For hierarchical and complex designs, identification of the appropriate level for tests and full reporting of outcomes
[ ]	[x]	Estimates of effect sizes (e.g. Cohen's *d*, Pearson's *r*), indicating how they were calculated

Our web collection on statistics for biologists contains articles on many of the points above.

Software and code

Policy information about availability of computer code

Data collection	Computational data was collected using a modified AlphaFold Multimer pipeline. The modified pipeline is provided as singularity files available in zenodo (https://doi.org/10.5281/zenodo.21232148)
Data analysis	Figures from computational data were plotted through Python using Matplotlib v3.10.5 and Seaborn v0.12.2 modules.<br>Statistical analysis and bar graphs from experimental data were performed using GraphPad Prism v11.0.<br>Protein interaction network diagrams were generated using Cytoscape v10.3.<br>Protein structure images were generated using ChimeraX v1.8.

For manuscripts utilizing custom algorithms or software that are central to the research but not yet described in published literature, software must be made available to editors and reviewers. We strongly encourage code deposition in a community repository (e.g. GitHub). See the Nature Portfolio guidelines for submitting code & software for further information.

Data

Policy information about availability of data

All manuscripts must include a data availability statement. This statement should provide the following information, where applicable:

- Accession codes, unique identifiers, or web links for publicly available datasets
- A description of any restrictions on data availability
- For clinical datasets or third party data, please ensure that the statement adheres to our policy

We have included this information in the Data availability section of the manuscript.

Research involving human participants, their data, or biological material

Policy information about studies with human participants or human data. See also policy information about sex, gender (identity/presentation), and sexual orientation and race, ethnicity and racism.

Reporting on sex and gender	*Use the terms sex (biological attribute) and gender (shaped by social and cultural circumstances) carefully in order to avoid confusing both terms. Indicate if findings apply to only one sex or gender; describe whether sex and gender were considered in study design; whether sex and/or gender was determined based on self-reporting or assigned and methods used. Provide in the source data disaggregated sex and gender data, where this information has been collected, and if consent has been obtained for sharing of individual-level data; provide overall numbers in this Reporting Summary. Please state if this information has not been collected. Report sex- and gender-based analyses where performed, justify reasons for lack of sex- and gender-based analysis.*
Reporting on race, ethnicity, or other socially relevant groupings	*Please specify the socially constructed or socially relevant categorization variable(s) used in your manuscript and explain why they were used. Please note that such variables should not be used as proxies for other socially constructed/relevant variables (for example, race or ethnicity should not be used as a proxy for socioeconomic status). Provide clear definitions of the relevant terms used, how they were provided (by the participants/respondents, the researchers, or third parties), and the method(s) used to classify people into the different categories (e.g. self-report, census or administrative data, social media data, etc.) Please provide details about how you controlled for confounding variables in your analyses.*
Population characteristics	*Describe the covariate-relevant population characteristics of the human research participants (e.g. age, genotypic information, past and current diagnosis and treatment categories). If you filled out the behavioural & social sciences study design questions and have nothing to add here, write "See above."*
Recruitment	*Describe how participants were recruited. Outline any potential self-selection bias or other biases that may be present and how these are likely to impact results.*
Ethics oversight	*Identify the organization(s) that approved the study protocol.*

Note that full information on the approval of the study protocol must also be provided in the manuscript.

Field-specific reporting

Please select the one below that is the best fit for your research. If you are not sure, read the appropriate sections before making your selection.

[x] Life sciences [ ] Behavioural & social sciences [ ] Ecological, evolutionary & environmental sciences

For a reference copy of the document with all sections, see nature.com/documents/nr-reporting-summary-flat.pdf

Life sciences study design

All studies must disclose on these points even when the disclosure is negative.

Sample size	No statistical methods were used to predetermine sample size. Sample sizes were chosen based on standard practice in the field, where three independent biological replicates are widely accepted as sufficient to establish reproducibility and to support statistical comparison for the types of quantitative assays used here. For quantified experiments, n = 3 biological replicates from independent experiments were used, which provided sufficient reproducibility to detect the effects reported and is consistent with sample sizes commonly used for comparable assays in the field. Certain qualitative or representative biochemical analyses (e.g. selected western blots and BN-PAGE analyses) were performed with n = 2 independent biological replicates; these were used to confirm reproducibility of a representative result rather than for statistical testing, and consistent results were obtained across replicates.
Data exclusions	No data were excluded.
Replication	The data from replication of biological experiments are provided as multiple data points in the figures.
Randomization	The yeast experiments originate from a random yeast colony. For experiments involving mammalian cells, the cells were seeded on multiple plates and were randomly selected. For experiments involving control and knockout lines, randomization was performed in the experimental procedure - assigning random wells in a seahorse flux analyzer for respiration, shuffling order of homogenization during mitochondrial

	isolation etc.
Blinding	No blinding was performed in our experiments. The experimental outcomes were objective, quantitative measurements from instruments (mass spectrometry, ICP-MS, oxygen consumption) or standardized biochemical assays (western blotting, BN-PAGE) that are not subject to subjective interpretation, so blinding was not relevant.

Reporting for specific materials, systems and methods

We require information from authors about some types of materials, experimental systems and methods used in many studies. Here, indicate whether each material, system or method listed is relevant to your study. If you are not sure if a list item applies to your research, read the appropriate section before selecting a response.

Materials & experimental systems

n/a	Involved in the study
☐	☒ Antibodies
☐	☒ Eukaryotic cell lines
☒	☐ Palaeontology and archaeology
☒	☐ Animals and other organisms
☒	☐ Clinical data
☒	☐ Dual use research of concern
☒	☐ Plants

Methods

n/a	Involved in the study
☒	☐ ChIP-seq
☒	☐ Flow cytometry
☒	☐ MRI-based neuroimaging

Antibodies

Antibodies used	COA4, 1:500 (ab105678; Abcam);<br>NDUFB8, 1:1000 (ab110242; Abcam);<br>SDHB, 1:1000 (ab14714; Abcam);<br>UQCRFS1, 1:1000 (ab14746; Abcam);<br>COX1, 1:1000 (ab14705; Abcam);<br>COX2, 1:1000 (ab198286; Abcam)<br>ATP5A, 1:20,000 (ab14748; Abcam);<br>ACO2, 1:500 (MA535527; Thermo Fisher Scientific);<br>COX11, 1:500 (11498-1-AP; ProteinTech);
Validation	Validation of antibody is provided by the supplier:<br>Anti-NDUFB8 antibody [20E9DH10C12] (ab110242) is a mouse monoclonal antibody and is validated for use in Western Blot (WB), Immunohistochemistry (IHC-Fr) in Cow, Human, Mouse, Rat samples. (https://www.abcam.com/en-us/products/primary-antibodies/ndufb8-antibody-20e9dh10c12-ab110242)<br>Anti-SDHB antibody [21A11AE7] (ab14714) is a mouse monoclonal antibody and is validated for use in Flow Cyt, ICC/IF, IHC-Fr, WB in cow, human, mouse, rat samples. (https://www.abcam.com/en-us/products/primary-antibodies/sdhb-antibody-21a11ae7-ab14714)<br>Anti-UQCRFS1 was tested to work in human and expected to work in mouse, cow, and rat. (https://doc.abcam.com/datasheets/active/ab14746/en-us/uqcrfs1-risp-antibody-5a5-ab14746.pdf)<br>Anti-MTCO1 (COX1) antibody [1D6E1A8] (ab14705) is a House Mouse recombinant monoclonal antibody and is validated for use in Flow Cyt, ICC, IHC-P, WB in human, recombinant fragment samples. Anti-MTCO1 antibody [1D6E1A8] (ab14705) has been cited over 521 times in peer reviewed journals and is trusted by the scientific community. (https://www.abcam.com/en-us/products/primary-antibodies/mtco1-antibody-1d6e1a8-ab14705)<br>Anti-MTCO2 (COX2) antibody (ab198286; Abcam) Rabbit Polyclonal MTCO2 antibody. Suitable for WB, IHC-P and reacts with Mouse, Human samples. Cited in 18 publications. Immunogen corresponding to Synthetic Peptide within Human MT-CO2. (https://www.abcam.com/en-us/products/primary-antibodies/mtco2-antibody-ab198286)<br>Anti-ATP5A antibody [15H4C4] - Mitochondrial Marker (ab14748) is a mouse monoclonal antibody and is validated for use in Flow Cyt, ICC/IF, IHC-P, WB in human, mouse, rat samples. Anti-ATP5A antibody [15H4C4] - Mitochondrial Marker (ab14748) has been cited over 553 times in peer reviewed journals and is trusted by the scientific community. (https://www.abcam.com/en-us/products/primary-antibodies/atp5a-antibody-15h4c4-mitochondrial-marker-ab14748)<br>ACO2 antibody specificity was demonstrated by siRNA mediated knockdown of target protein. MCF7 cells were transfected with Aco2; Aconitase siRNA and decrease in signal intensity was observed in Western Blot application using Aconitase 2 Recombinant Rabbit Monoclonal Antibody (ARC1072) (Product # MA5-35527). (https://www.thermofisher.com/antibody/product/Aconitase-2-Antibody-clone-0P3H3-Recombinant-Monoclonal/MA5-35527)<br>COX11 antibody was validated in-house through over-expression of COX11 protein in 293T cells which corresponded to an increase in protein abundance at the appropriate size seen through western blotting (see Fig. 4f-g).<br>COA4 antibody was validated in-house through knockout in MCH58 cells (see fig. 4h)

Eukaryotic cell lines

Policy information about cell lines and Sex and Gender in Research

Cell line source(s)	293T cells (ATCC CRL-3216) were obtained from the ATCC cell repository. The MCH58 cell line is a gift from Dr. Eric

	Shoubridge.
Authentication	The cell line was maintained at low passage number and observed for their expected morphology.
Mycoplasma contamination	The cells used tested negative for mycoplasma using lonza mycoplasma detection kit (LT07-118).
Commonly misidentified lines (See ICLAC register)	None

Plants

Seed stocks	None
Novel plant genotypes	None
Authentication	None
