# Supplementary material for: The predicted interactome of the human mitochondrial proteome
Source: Nat Commun. 2026 Aug 25;17:10162. doi: 10.1038/s41467-026-77112-z (PMC13601606; doi:10.1038/s41467-026-77112-z)
Supplement: Supplementary file 4 [file 41467_2026_77112_MOESM7_ESM.pdf]

nature portfolio

Peer Review File

The predicted interactome of the human mitochondrial proteome

Corresponding Author: Professor Vishal Gohil

This file contains all reviewer reports in order by version, followed by all author rebuttals in order by version.

Version 0:

Reviewer comments:

Reviewer #1

(Remarks to the Author)
The authors apply the AFM algorithm to identify interacting protein pairs within the mitochondrial proteome, score the predicted interactions, and rank them to infer likely true positives. While the overall concept is interesting, the study is limited by the fact that the authors rely solely on AFM predictions without performing unbiased or extensive experimental validation of the proposed protein–protein interactions. In the absence of rigorous validation, the work does not substantially advance the field beyond what individual researchers can already obtain by querying existing online structure-prediction platforms for specific protein pairs.

Specific comments

Reliability of AFM for membrane proteins:
Recent studies show that ipTM values are less reliable for transmembrane proteins due to differences in interface geometry and domain organization. Because AFM models are predominantly trained on soluble proteins deposited in the PDB, the authors should evaluate whether their analysis produces similar false-positive rates for soluble versus membrane-associated or membrane-embedded proteins. For example, they could compare their predictions with available complexome profiling or mitochondrial XL-MS datasets.

Assumption that interacting proteins share function:
The authors assume that if two proteins interact, they must be part of the same functional complex or pathway, allowing the function of a known subunit to be used to infer the role of an “orphan” protein. However, proteins frequently interact without sharing a common function, for example, scaffolding proteins, chaperones, and proteases may interact with their substrates without being part of the same pathway.

Claim of defining the CoQ metabolon:
The authors state that they have “defined the CoQ metabolon,” but the presented data are insufficient to demonstrate the existence of a large complex containing all CoQ pathway components. At minimum, the authors should perform BN-PAGE followed by a second dimension to determine whether the predicted interacting pairs co-migrate within the same native complex.

Interpretation of Coq3, Coq5, and Coq6 interactions:
The fact that Coq3 interacts with Coq5 and Coq6 does not imply that Coq5 and Coq6 interact with each other; indeed, AFM does not predict such an interaction. Can the authors confirm whether Coq3, Coq5, and Coq6 all interact directly, and whether these proteins reside within the same complex?

COA4–COX11 co-immunoprecipitation:
The co-IP of COA4 and COX11 in yeast relies on chemical crosslinking and a single bait configuration without reciprocal or tag-free validation, raising the possibility of crosslinking- or tag-dependent artefacts. In addition, the interaction is not confirmed in human cells.

Effect of COA4 knockout on COX11:
The manuscript does not clearly explain how COA4 KO affects COX11 stability, localization, or function.

AFM does not predict COX1–COA4 interaction, so the rationale for attempting to experimentally validate this specific interaction is unclear.

Copper delivery function not sufficiently tested. If COA4 is specifically involved in copper delivery, it is unclear why the authors only examine OXPHOS phenotypes and do not directly measure copper levels or copper-dependent enzymatic functions. This omission weakens the causal interpretation of the observed decrease in oxygen consumption. The link between complex IV assembly defects and reduced respiration is not demonstrated using genetic or chemical rescue approaches.

Minor comments

The text does not follow the order of the figure panels. For example, Figure 2 appears immediately after Figure 1C, and the remaining panels are only referenced later in the text, making the manuscript difficult to follow.

(Remarks on code availability)

Reviewer #2

(Remarks to the Author)
This authors performed a global analysis of predictions of mitochondrial protein-protein interactions using structure based tool AlphaFold-Multimer AFM. AFM is a tool based on AlphaFold2 to predict protein machineries. They screened > 600000 interaction pairs that led to a database of 2895 interactions. Notably, 85 previously unknown mitochondrial proteins poped up that may represent interesting novel players in mitochondrial biochemical pathways. Including different eukarotes (important to support/validate their findings) in their analysis can reveal novel insights into conserved (core) pathways. The authors validated and demonstrated this elegantly for CoenzymeQ biosynthesis pathway and a novel pathway for copper insertion into the COX machinery.
The presented database is the first systematic AFM screening of an organellar proteome and is a rich source for the mitochondrial research community and I expect the field to use this data as an idea generator for novel explorations. Alltogether this is an important paper that merits publication in Nat. Commun.
Nevertheless, I have a few point that I find important to include/change or explain in a reivised version of the manuscript:
This study adresses the mitochondrial community (and less AI driven research community). It would therefore be good to explain some of the terms and limitations also for non AFM experts. E.g. what is ipTM-score, how one should interpret AFM-based interaction predictions, limitations on claims related to affinities, stoichiometry etc?
It would be good if the accompanied web site can be more easily found (without the bulky link to the Gohil lab); it would also be good if the search terms could be handled more flexible or offer alternative names. If one does not hit the correct nomenclature, it is laborious to find the targets of interest. For some proteins the GENE nomenclature is used.
Morevover, it would be helpful if the interaction lists (ideally on the mitomach site) can indicate already known (so validated by this approach!) and novel/unkown interactions.
The Coa4 interactome reveals Pth5 phosphatase (located in the IMS) as strong interactor (Fig 4 e); Does this mean that the copper insertion pathway could be modulated by reversible phosphorylation? Anything known on phosphorylation sites of the involved players? A comment on this would be good. What about the human Pth5 homolog?
Do the authors consider Hfd1 (Coq4 interactome; Fig 3c) as part of the CoQ machinery? As this is an outer membrane protein this finding might be explained a bit and set into context.

(Remarks on code availability)

Reviewer #3

(Remarks to the Author)
I read the manuscript with great interest and enjoyment. We have worked for over a decade on detecting protein–protein interactions by analyzing patterns in multiple sequence alignments. While we are well aware that such analyses can generate intriguing hypotheses about protein interactions and functions, successfully translating these hypotheses into experimentally validated discoveries is something we particularly appreciate. I do not have sufficient expertise to critically assess the experimental aspects of this work, so my comments below focus exclusively on the computational analysis.

1. I appreciate that the authors evaluate performance using a signal-to-noise ratio of 1:100. This choice appears appropriate for a mitochondrial interaction screen, where each protein is expected to interact with perhaps 5–10 partners among roughly 1,000 mitochondrial proteins.

2. In some of our own benchmarks (not specifically on mitochondrial proteins), we found that iPTM can be a weaker indicator of interactions than other metrics, such as contact probability derived from distogram outputs or iPAE. I was wondering whether the authors explored alternative indicators and, if so, how their performance compared.

3. I am curious whether integrating predictions across multiple organisms might further improve performance. I understand that a comprehensive benchmark could be computationally demanding, but cross-species integration may provide a promising avenue to enhance PPI prediction accuracy.

4. In our own large-scale screens, we frequently observe systematic errors that limit performance. In particular, some proteins appear to have an intrinsic tendency to produce high interaction scores with many partners. I wonder whether the authors have observed a similar effect in their analysis and, if so, how they addressed it.

5. Another recurring issue we encounter is that homologous protein pairs tend to contribute disproportionately to false positives. Relatedly, if protein A interacts with protein B, we often observe elevated scores between A and B′, a paralog of B. These issues may be less pronounced in the mitochondrial context, where close homologs are relatively uncommon, but I would nonetheless be interested in the authors' experience regarding this potential source of error.

(Remarks on code availability)

Version 1:

Reviewer comments:

Reviewer #1

(Remarks to the Author)
I thank the authors for addressing my concerns. The revised ms has substantially improved with additional experimental validations, and as it is now, suitable for publication in Nat. Commun.

(Remarks on code availability)

Reviewer #2

(Remarks to the Author)
The authors have addressed my comments in a satisfactory manner. The revised manuscript is more accessible to non-AFM specialists, with a clearer explanation of the ipTM score and an explicit discussion of limitations regarding affinity, stoichiometry, and complex composition. The website URL has been simplified and the search functionality and known/novel interaction annotations have been improved. Congratulations to the authors for this important work.

(Remarks on code availability)

Reviewer #3

(Remarks to the Author)
I do not have further concerns. Thanks to authors for a great piece of work.

(Remarks on code availability)

Open Access This Peer Review File is licensed under a Creative Commons Attribution 4.0 International License, which permits use, sharing, adaptation, distribution and reproduction in any medium or format, as long as you give appropriate credit to the original author(s) and the source, provide a link to the Creative Commons license, and indicate if changes were made.
In cases where reviewers are anonymous, credit should be given to 'Anonymous Referee' and the source.
The images or other third party material in this Peer Review File are included in the article's Creative Commons license, unless indicated otherwise in a credit line to the material. If material is not included in the article's Creative Commons license and your intended use is not permitted by statutory regulation or exceeds the permitted use, you will need to obtain permission directly from the copyright holder.
To view a copy of this license, visit https://creativecommons.org/licenses/by/4.0/

REVIEWER COMMENTS

Reviewer #1 (Remarks to the Author):

The authors apply the AFM algorithm to identify interacting protein pairs within the mitochondrial proteome, score the predicted interactions, and rank them to infer likely true positives. While the overall concept is interesting, the study is limited by the fact that the authors rely solely on AFM predictions without performing unbiased or extensive experimental validation of the proposed protein–protein interactions. In the absence of rigorous validation, the work does not substantially advance the field beyond what individual researchers can already obtain by querying existing online structure-prediction platforms for specific protein pairs.

We are glad that the reviewer finds the overall concept of our work "interesting." Below we address the concerns raised about the limitations of our study and whether it substantially advances the field.

Experimental validation of the proposed protein-protein interactions:
The reviewer raises an important point. To address this issue, we have added a new supplementary table (Table S3) that cross-validates our predictions against published mitochondrial cross-linking mass spectrometry data, proteome-wide co-immunoprecipitation data, the binary human reference interactome, and STRING scores for all 2,895 hits. Collectively, these orthogonal datasets provide independent support for ~43% of our predictions, giving researchers a basis for prioritizing which interactions to pursue experimentally.

We also considered independently validating our predictions using yeast two hybrid and immunoprecipitation – mass spectrometry (IP-MS)-based approaches. However, these high-throughput experimental methods are themselves plagued by false positives, and when used in isolation without additional experimentation, they make a 'one-shot' validation of a proteome-wide dataset neither straightforward nor particularly meaningful. Indeed, a recent study found only around 5% overlap among high-confidence interactions reported across UniProt, STRING, and BioGRID[1]. Therefore, reliable validation of every predicted interaction in a proteome-wide study like this would require a coordinated effort using multiple techniques across many laboratories with very different areas of expertise — which is beyond the scope of the current study.

How is this study different from existing online structure prediction platforms for specific protein pairs?
Running online platforms such as AlphaFold3 or AlphaFold-Multimer on Colab Notebook to evaluate the interaction between individual protein pairs is a fundamentally different exercise from doing so systematically across the whole organellar/cellular proteome, with

a scoring framework designed to separate signal from noise like our study does. For example, if a researcher wants to identify a putative interacting partner of their favorite protein using the above-mentioned online platforms, then they will have to manually feed in protein sequences of target proteins (which can range in 1000's, depending on the organism, organelle etc.). AlphaFold3 only allows 30 predictions per day, whereas with the other platform there is limited GPU availability to screen for putative interactors (usually less than 30 interactions per day). Thus, a systematic screening for identifying partners for just a single query protein can take months and is not practically feasible. Moreover, the output from such an exercise produces a score and a structural model that is difficult to interpret in the absence of the reference scores for all the interactions and rigorous statistics (False Discovery Rates, Baseline scores etc.) that our study provides.

Does this study substantially advance the field?

Our study advances the field in the following manner:

(1) As described above, while online platforms offer targeted queries, none provides a ranked, proteome-wide mitochondrial interaction landscape — nor is such an undertaking easily within reach for most research groups. Our creation of the MitoMatch inventory required over 100,000 GPU hours on a high-performance computing facility, a compute capability that is simply not accessible to the wider community. Therefore, making these predictions freely available is a major contribution to the broad research community.

(2) Due to transient and context-specific interactions, some proteins are particularly recalcitrant to functional analyses by classical biochemical and genetic approaches. Our compendium offers important clues regarding their involvement in a specific mitochondrial pathway by structurally linking them to known proteins within those pathways/processes. Therefore, we believe our MitoMatch compendium will serve as a valuable hypothesis-generating resource.

(3) Finally, guided by the MitoMatch prediction, our in-depth experimental investigation (including new experiments suggested by this reviewer) of an uncharacterized human mitochondrial protein, COA4, helped us link it to a specific step in the mitochondrial copper delivery pathway. This serves as an example of how our repository can help expedite the functional characterization of the mitochondrial proteome.

Specific comments

Reliability of AFM for membrane proteins:

Recent studies show that ipTM values are less reliable for transmembrane proteins due to differences in interface geometry and domain organization. Because AFM models are predominantly trained on soluble proteins deposited in the PDB, the authors should evaluate whether their analysis produces similar false-positive rates for soluble versus membrane-associated or membrane-embedded proteins. For

example, they could compare their predictions with available complexome profiling or mitochondrial XL-MS datasets.

We thank the reviewer for raising this point, which we recognize is an important concern in the field. To test the reliability of AlphaFold Multimer in predicting protein-protein interactions involving proteins with transmembrane domains, we first categorized our mitochondrial test dataset as soluble or transmembrane domain-containing proteins using their UniProt annotations. We then plotted the ipTM score distributions of the non-interacting pairs after categorizing them as pairs where both are soluble, or one protein in a pair has a transmembrane domain, or both proteins are transmembrane domain containing proteins. As shown below, we did not observe any drastic change in the ipTM score distribution for protein pairs involving transmembrane proteins (Response Fig. 1a). This is further confirmed by the precision-recall curve plotted using the same three categories of non-interacting pairs, appropriately weighted to represent a 1:100 true:false pairs. We did not see any change in the recall at 90% precision cutoff (Response Fig. 1b).

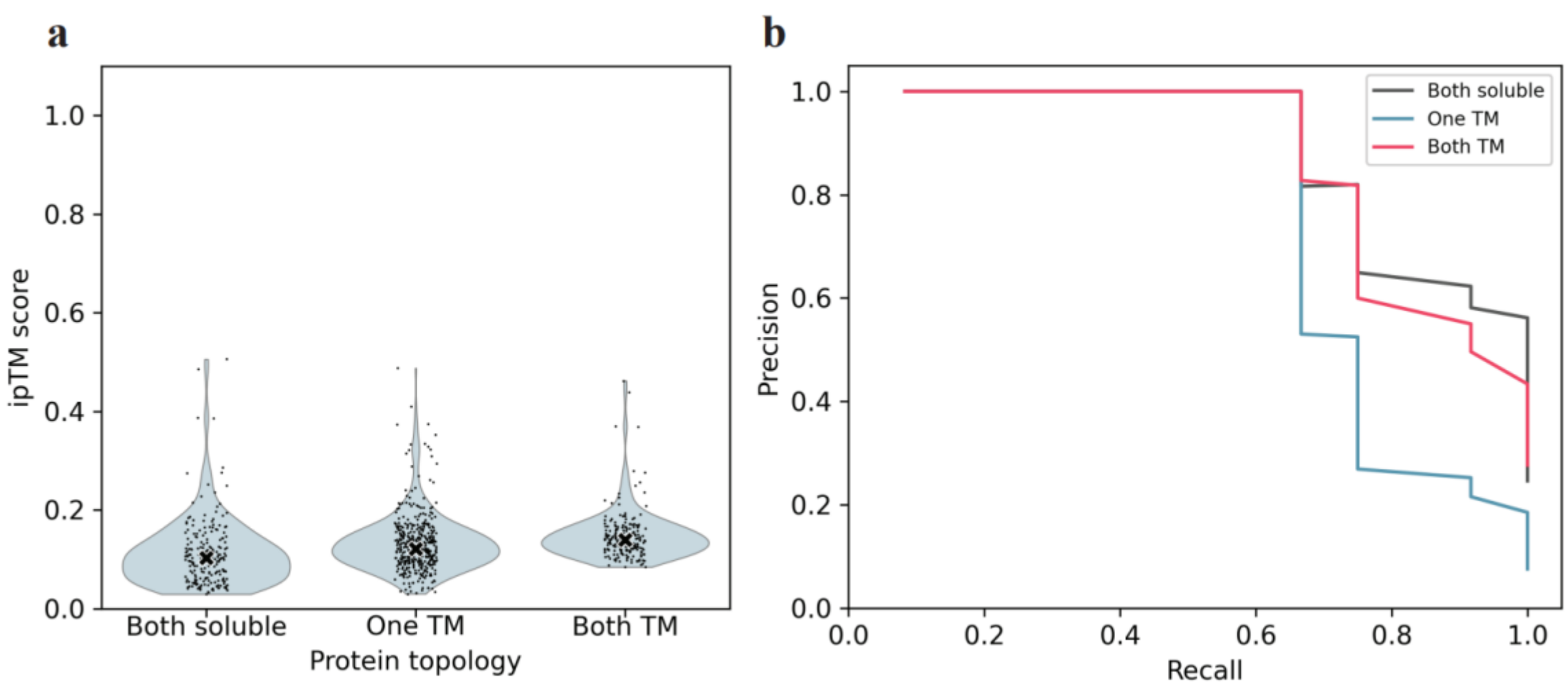

Response Figure 1: Effect of transmembrane domains on the success rate of AlphaFold Multimer based interaction prediction. **a,** A plot of the mean ipTM scores of non-interacting protein pairs from the recent PDB dataset. Transmembrane domain annotations were retrieved from UniProt and the protein pairs were categorized into three classes – both proteins are soluble, one protein has a transmembrane domain, and both proteins have a transmembrane domain. b, Precision recall curve plotted using non-interacting pairs from the above-mentioned classes at a 1:100 true:false ratio.

Since interactions between transmembrane proteins are expected to be predominantly through hydrophobic residues of the interacting pair and for soluble proteins the interacting residues are likely hydrophilic, we looked at the distribution of interface residue pairs for our 2895 MitoMatch hits, particularly heterochain contacts involving hydrophobic-to-hydrophobic residues or charged/polar-to-charged/polar residues. We did not observe

any correlation between the ipTM scores and the fraction of interface residue pairs that are both hydrophobic (Response Fig. 2a) or both charged/polar (Response Fig. 2b).

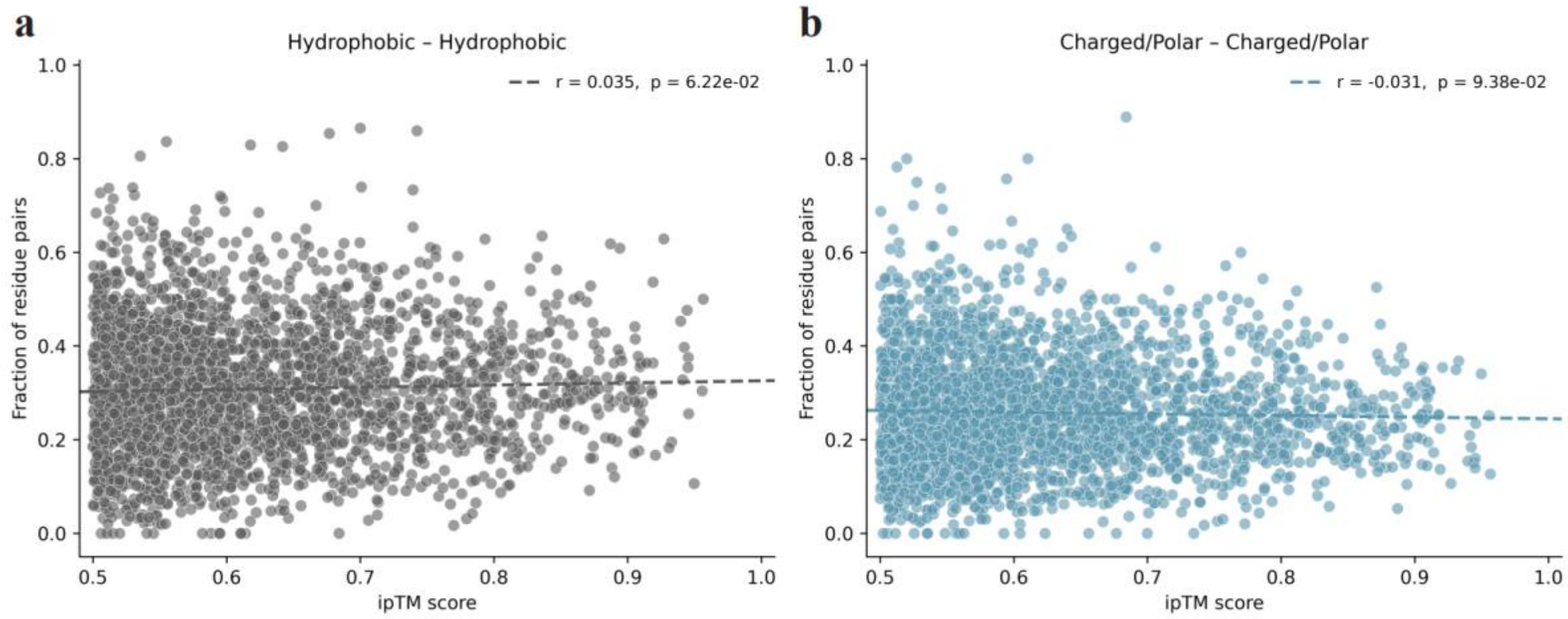

Response Figure 2: Correlation of ipTM scores with physicochemical properties of the predicted interface. **a**, Fraction of hydrophobic residue pairs at the predicted interface (best AFM model) versus mean ipTM score across all 2,895 MitoMatch hits. **b**, Fraction of charged or polar residue pairs at the predicted interface (best AFM model) versus mean ipTM score across all MitoMatch hits.

Together, we conclude that our dataset is not conflated with false positives involving transmembrane proteins. This is because AlphaFold does not regurgitate learnt interactions from the PDB but rather works as a multiple sequence alignment (MSA) decoder that can predict novel interfaces from residue coevolution, provided the MSAs contain sufficient depth (>25 diverse sequences). The median MSA depth for mitochondrial protein pairs in our dataset is ~170 diverse sequences, far beyond the expected baseline depth, and therefore we expect a strong performance for mitochondrial protein pairs.

Assumption that interacting proteins share function:
The authors assume that if two proteins interact, they must be part of the same functional complex or pathway, allowing the function of a known subunit to be used to infer the role of an "orphan" protein. However, proteins frequently interact without sharing a common function, for example, scaffolding proteins, chaperones, and proteases may interact with their substrates without being part of the same pathway.

We apologize for the confusion – that is not what we meant. We have now added the following to the text in the Discussion section to clarify what we meant.

"MitoMatch provides a resource for annotating uncharacterized mitochondrial proteins by identifying the proteins they interact with. A predicted interaction with a characterized protein serves as a starting point for hypothesis generation; the orphan protein may turn

out to be a functional subunit of the same pathway, a regulatory factor, a chaperone, or an assembly factor."

Claim of defining the CoQ metabolon:

The authors state that they have "defined the CoQ metabolon," but the presented data are insufficient to demonstrate the existence of a large complex containing all CoQ pathway components. At minimum, the authors should perform BN-PAGE followed by a second dimension to determine whether the predicted interacting pairs co-migrate within the same native complex.

We certainly agree that our data in isolation would be insufficient to demonstrate what the reviewer notes above. However, our work here builds upon a series of prior studies, including our own, that support the existence of this complex. For example, numerous previous studies have demonstrated the co-migration of yeast Coq proteins in high molecular weight ranges via 2D Blue Native PAGE analyses[2–4], which have formed the basis of describing the CoQ synthome/complex Q. However, this complex is dynamic in response to changing pathway flux, recalcitrant to traditional purification and structural characterization approaches, and perhaps not even a singular, uniform complex[5]. These features have made it difficult to define clear protein-protein interactions — the very motivation for our alternative computational approach here.

Despite these challenges, following this reviewer's comment, we performed 2D BN-PAGE experiments on mitochondria isolated from yeast with Coq proteins tagged endogenously with GFP and found that Coq3, Coq4, Coq6, and Coq9 showed migration patterns that overlap in a high molecular weight range (Response Fig. 3a). However, not all migration patterns were uniform or consistent, once again suggesting that these proteins could have different interaction profiles, perhaps forming a series of subcomplexes. As an alternative approach, we subjected solubilized mitochondria to analytical size exclusion chromatography and monitored the elution pattern of endogenously tagged Coq proteins. Similarly, we found that these complex Q proteins (Coq3, Coq4, Coq5, Coq6, Coq9, and Coq11) demonstrated overlapping elution patterns indicative of high molecular weight range complexes (Response Fig. 3b), though this once again suggests a lack of uniformity or multiple potential complexes. Finally, we examined a published high-resolution complexome profiling dataset from yeast[6] and found that the same Coq proteins co-migrated with profiles indicative of a potential complex at ~1200 kDa, though there was also co-migration of these proteins in a potential lower molecular weight complex of ~250 kDa (Response Fig. 3c).

Taken together, these experiments reconfirmed prior observations and further demonstrated the utility of our computational approaches. We believe that our high-confidence predictions offer testable insights that we and others could not produce from

experimental approaches. Nonetheless, we do not assert that our findings provide an unequivocal demonstration of complex Q stoichiometry or architecture and have now revised our text to make this clearer to our audience and have removed the phrase “defined the CoQ metabolon”.

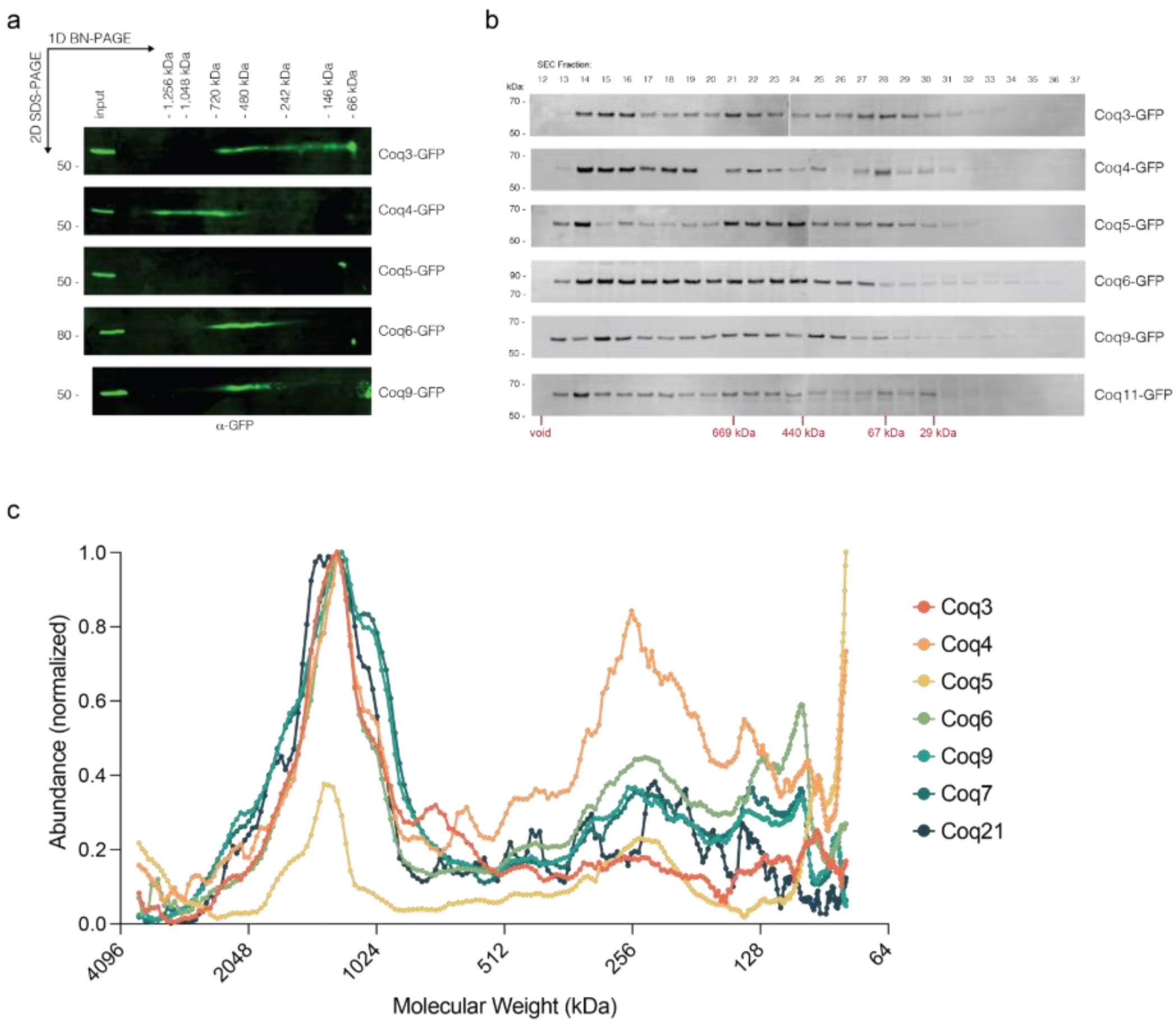

Response Figure 3. **a,** Two-dimensional Blue Native PAGE of digitonin-solubilized mitochondria expressing the indicated Coq protein with an endogenous GFP tag. Western blotting was performed against GFP. **b,** SDS-PAGE and anti-GFP western blot analysis of fractions recovered from analytical size exclusion chromatography performed on digitonin-solubilized mitochondria isolated from strains expressing the indicated Coq protein with an endogenous GFP tag. **c,** Normalized abundance profiles of complex Q proteins as analyzed by high-resolution complexome profiling[5].

Interpretation of Coq3, Coq5, and Coq6 interactions:

The fact that Coq3 interacts with Coq5 and Coq6 does not imply that Coq5 and Coq6 interact with each other; indeed, AFM does not predict such an interaction. Can the authors confirm whether Coq3, Coq5, and Coq6 all interact directly, and whether these proteins reside within the same complex?

We agree with the reviewer's assessment that AFM does not predict a direct binary interaction between Coq5 and Coq6, though it does predict interactions between Coq3/Coq5, and Coq3/Coq6. However, the crosslinking data does not provide information regarding binary interactions within the complex; rather, it stabilizes the entire complex and identifies all resident components. From that, we can propose that Coq3, Coq5, and Coq6 all reside within the same complex, though the interactions of Coq3/Coq5 and Coq3/Coq6 could represent parts of unique sub-complexes or could be facilitated by different interaction surfaces on Coq3 as we suggest with the predicted structural modeling in Fig. 3l-m.

COA4–COX11 co-immunoprecipitation:

The co-IP of COA4 and COX11 in yeast relies on chemical crosslinking and a single bait configuration without reciprocal or tag-free validation, raising the possibility of crosslinking- or tag-dependent artefacts. In addition, the interaction is not confirmed in human cells.

Given that our MitoMatch predictions and the follow-up experimentation on COA4 were using the human cell lines, we now provide reciprocal co-IP of human 293T cells transfected with COA4-V5 and COX11-FLAG (Response Fig. 4). We also added this data as Figures 4f and 4g in the revised manuscript.

Immunoprecipitation using Anti-FLAG beads identified COA4-V5 as an interacting partner of COX11 (Figure 4f and below), validating our MitoMatch prediction. We also recover COX1 as an interacting partner of COX11 (Figure 4f and below), which serves as our positive control because COX11 is a known metallochaperone of COX1. A recent study has shown that COX1 and COX2 assembly processes are highly coordinated, with COX11 also interacting with newly synthesized COX2[7]. This experimental result is consistent with our MitoMatch dataset, which predicts an interaction of COX11 with COX2. Importantly, we also recover COX2 as an interactor of COX11 in our co-IP (Figure 4f and below), validating both our prediction and reproducing prior results.

A reciprocal IP experiment using Anti-V5 beads identified COX11-FLAG as an interacting partner of COA4 (Figure 4g and below), validating MitoMatch prediction. Notably, unlike with COX11-IP, we did not recover COX1 and COX2 proteins in our COA4-V5-IP, which is in line with our MitoMatch prediction that does not predict direct interaction between COA4 with either COX1 or COX2. Together, these results show that COA4 interact with COX11 in the copper delivery pathway to COX1 and is consistent with our prior yeast genetics study[8].

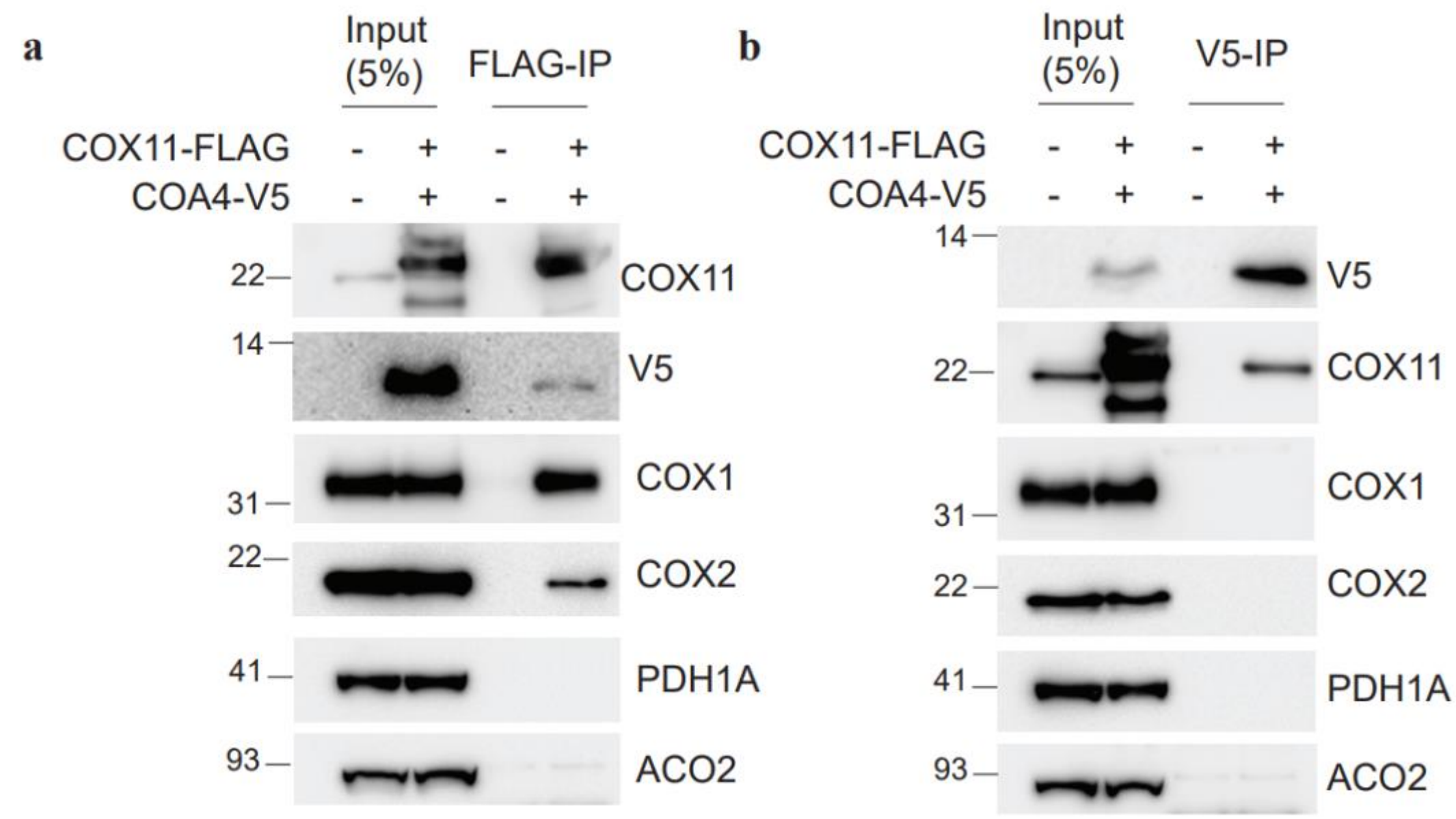

Response Figure 4: a, Co-immunoprecipitation of COX11-FLAG using Anti-FLAG beads followed by western blotting to probe for interacting partners. **b,** Co-immunoprecipitation of COA4-V5 using Anti-V5 beads followed by western blotting to probe for interacting partners. **The above panels are added as Fig. 4f and g**

Effect of COA4 knockout on COX11:
The manuscript does not clearly explain how COA4 KO affects COX11 stability, localization, or function.

To address this important point, we performed western blotting analysis of COX11 abundance in WT and COA4-KO MCH58 cells. Deletion of COA4 also abolished COX11 abundance (Response Fig. 5 and Figure 4i in the revised manuscript). This is consistent with the idea that protein-protein interactions serve to stabilize the interacting partner. In line with this, we have also provided additional data validating the COA4-COX11 interaction (See above Response Fig. 4).

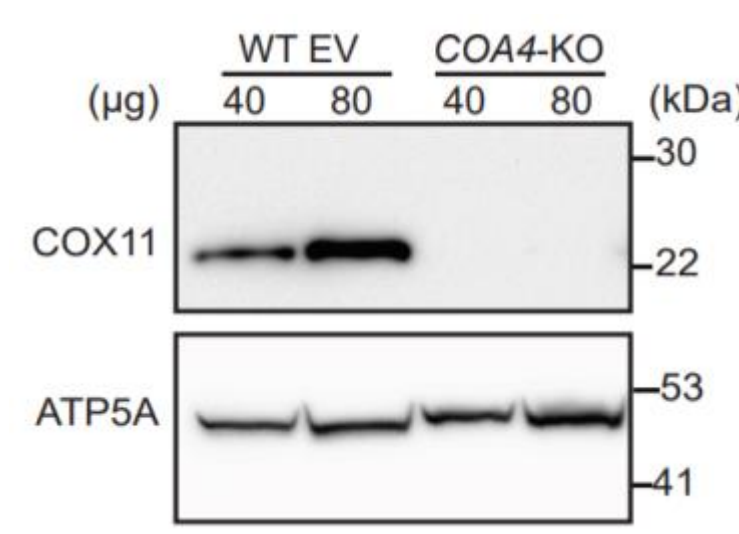

Response Figure 5: Western blotting analysis of COX11 levels in WT and COA4-KO cells. ATP5A is used as a loading control. **This panel is added as Fig. 4i**

AFM does not predict COX1-COA4 interaction, so the rationale for attempting to experimentally validate this specific interaction is unclear.

The goal was not to demonstrate a direct COX1-COA4 interaction but rather the

downstream impact of loss of COA4 on COX1 metalation. We probed for COX1 levels in COA4 knockout cells because failure of copper delivery to COX1 (which is expected when a copper delivery pathway is disrupted) is known to destabilize COX1 protein, resulting in its reduced abundance. Therefore, we use COX1 abundance as a marker to investigate the role of COA4 in copper delivery to COX1. We now provide a brief explanation of this in the text:

"Failure of copper delivery to COX1 is known to reduce its abundance. To assess if COA4-KO cells exhibit reduced abundance of COX1-containing complexes, we performed BN-PAGE followed by western blotting."

```
Copper delivery function not sufficiently tested. If COA4 is
specifically involved in copper delivery, it is unclear why the
authors only examine OXPHOS phenotypes and do not directly measure
copper levels or copper-dependent enzymatic functions. This omission
weakens the causal interpretation of the observed decrease in oxygen
consumption. The link between complex IV assembly defects and reduced
respiration is not demonstrated using genetic or chemical rescue
approaches.
```

We have now measured the mitochondrial copper levels in WT and COA4-KO cells and observed a 50% decrease in mitochondrial copper levels in COA4-KO cells, while the levels of other OXPHOS relevant metals such as Fe, Zn, and Mn, were not affected as shown below and Figure 4j + Supplementary Figure 11 in the revised manuscript). This specific decrease in copper levels further connect COA4 to the mitochondrial copper delivery pathway, in addition to its interaction with COX11, a known copper metallochaperone. Since the loss of COA4 triggers loss of its interacting partner (COX11) (Response Figure 5), we do not expect to rescue respiratory phenotype by chemical means. We had previously shown that COX11 overexpression in yeast *coa4* knockout does indeed rescue COX1 and mitochondrial respiration[8].

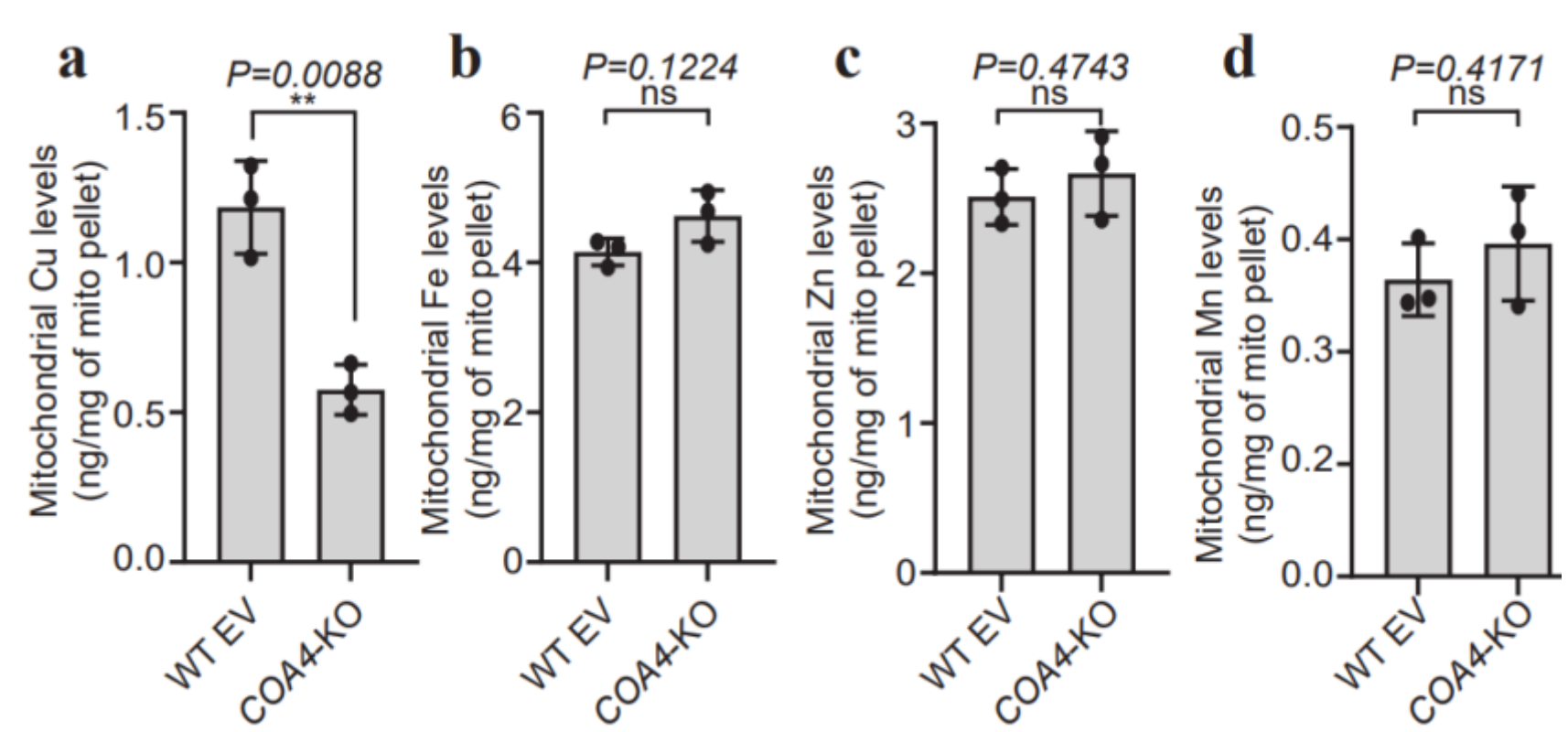

Response Figure 6: Measurement of mitochondrial (a) Cu, (b) Fe, (c) Zn, and (d) Mn levels using ICP-MS.
The above panels are added as Fig. 4j and Supplementary Fig. 11

Taken together, these new experiments inspired by the reviewer's comments, including reciprocal co-IP, decreased mitochondrial copper levels, and decreased COX11 abundance in COA4-KO, strongly link and place human COA4 in the copper delivery pathway to COX1 subunit of CcO.

Minor comments

The text does not follow the order of the figure panels. For example, Figure 2 appears immediately after Figure 1C, and the remaining panels are only referenced later in the text, making the manuscript difficult to follow.

We have ensured that text and figures follow the order in the revised manuscript.

Reviewer #2 (Remarks to the Author):

This authors performed a global analysis of predictions of mitochondrial protein-protein interactions using structure based tool AlphaFold-Multimer AFM. AFM is a tool based on AlphaFold2 to predict protein machineries. They screened > 600000 interaction pairs that led to a database of 2895 interactions. Notably, 85 previously unknown mitochondrial proteins poped up that may represent interesting novel players in mitochondrial biochemical pathways. Including different eukarotes (important to support/validate their findings) in their analysis can reveal novel insights into conserved (core) pathways. The authors validated and demonstrated this elegantly for CoenzymeQ biosynthesis pathway and a novel pathway for copper insertion into the COX machinery.

We thank the reviewer for succinctly summarizing the paper's main point and for highlighting the value of our computational predictions and the experimental validation of our predictions in the COQ biosynthesis and the copper delivery pathway to COX.

The presented database is the first systematic AFM screening of an organellar proteome and is a rich source for the mitochondrial research community and I expect the field to use this data as an idea generator for novel explorations.

Alltogether this is an important paper that merits publication in Nat. Commun.

Again, we thank the reviewer for recognizing the value of our dataset and for finding our work to be suitable for publication in *Nature Communications*. We fully agree that our MitoMatch resource will serve as a hypothesis generator to aid targeted experimentation to systematically de-orphan the mitochondrial proteome and will be a valuable resource to the larger mitochondrial community.

Nevertheless, I have a few point that I find important to include/change or explain in a reivised version of the manuscript:
This study adresses the mitochondrial community (and less AI driven research community). It would therefore be good to explain some of the terms and limitations also for non AFM experts. E.g. what is ipTM-score, how one should interpret AFM-based interaction predictions, limitations on claims related to affinities, stoichiometry etc?

We understand the reviewer's point and accordingly, we now provide a full description of the terms and limitations of this work for non-AFM experts as described below and in the revised manuscript. We have also streamlined the results section to make our study more accessible to the larger audience.

“The ipTM score is a confidence metric that was originally designed to assess the accuracy of predicted structures. We asked if we could repurpose this metric as a classifier cutoff score to distinguish interacting from non-interacting protein pairs. In this case, a score of 0 would indicate no interaction while a score of 1 would indicate a high-confidence interaction.”

“One of the strengths of AFM-based PPI predictions are that they are agnostic to biological or thermodynamic constraints and solely rely on evolutionary signals found within and between proteins to predict novel interaction interfaces in protein pairs from residue coevolution. However, the limitation of this approach is that it cannot provide binding affinities of the interaction pair. Finally, we note that many of our predicted interactions could happen in the cell in the context of a larger complex and may not exist as true heterodimers. Thus, our predictions do not cover either the stoichiometry or the composition of such complexes.”

It would be good if the accompanied web site can be more easily found (without the bulky link to the Gohil lab); it would also be good if the search terms could be handled more flexible or offer alternative names. If one does not hit the correct nomenclature, it is laborious to find the targets of interest. For some proteins the GENE nomenclature is used.

We have now moved our website to the domain “mitomatch.web.app”. We have used both the UniProt ID and the MitoCarta3.0 gene symbol for a given protein and now have also included alternate gene names from UniProt to ease searchability. Overall, the new website is more easily accessible and more informative.

Morevover, it would be helpful if the interaction lists (ideally on the mitomach site) can indicate already known (so validated by this approach!) and novel/unkown interactions.

We have now added this information in our website and the supplementary Table S3 to indicate which one of our interactions are already reported.

The Coa4 interactome reveals Pth5 phosphatase (located in the IMS) as strong interactor (Fig 4 e); Does this mean that the copper insertion pathway could be modulated by reversible phosphorylation? Anything known on phosphorylation sites of the involved players? A comment on this would be good. What about the human Pth5 homolog?

Following this insightful comment by the reviewer, we scoured through the literature to determine the potential significance of Ptc5 phosphatase interaction with Coa4. One high throughput study on post-translational modifications of yeast proteins reported phosphorylation of serine residue (S80) of yeast Coa4 protein[9]. However, no phosphorylation sites have been reported for the human COA4 protein and, critically, the

yeast S80 residue is not conserved. So currently the significance of potential reversible phosphorylation on the copper delivery pathway proteins remains unclear. We have now added the following text in the revised draft to expand on this finding:

"In addition, we also find novel interactors of Coa4, including the IMS-localized Ptc5 phosphatase, which suggests a role for this protein in regulating copper delivery to CcO through reversible phosphorylation of Coa4 in yeast."

Do the authors consider Hfd1 (Coq4 interactome; Fig 3c) as part of the CoQ machinery? As this is an outer membrane protein this finding might be explained a bit and set into context.

While Hfd1 is not typically considered to be a member of complex Q, we felt that it was notable given its role in CoQ head group biosynthesis, and thus we highlighted it in our data. To further explore this potential yet unexpected interaction, we turned to the published high-resolution mitochondrial complexome profiling dataset[6]. While Hfd1 does not co-migrate in the ~1200 kDa range with the full set of complex Q proteins, it does demonstrate some overlapping migration with Coq4 at a lower molecular weight (Response Fig. 7). This could suggest the presence of additional interactions or subcomplexes containing Coq4 that are distinct from the canonical complex Q, and even that Coq4 might have a second localization that puts it in close juxtaposition with Hfd1. We anticipate testing this further in future work; however, especially given that this direct interaction is not supported by our computational predictions, we did not pursue it further here.

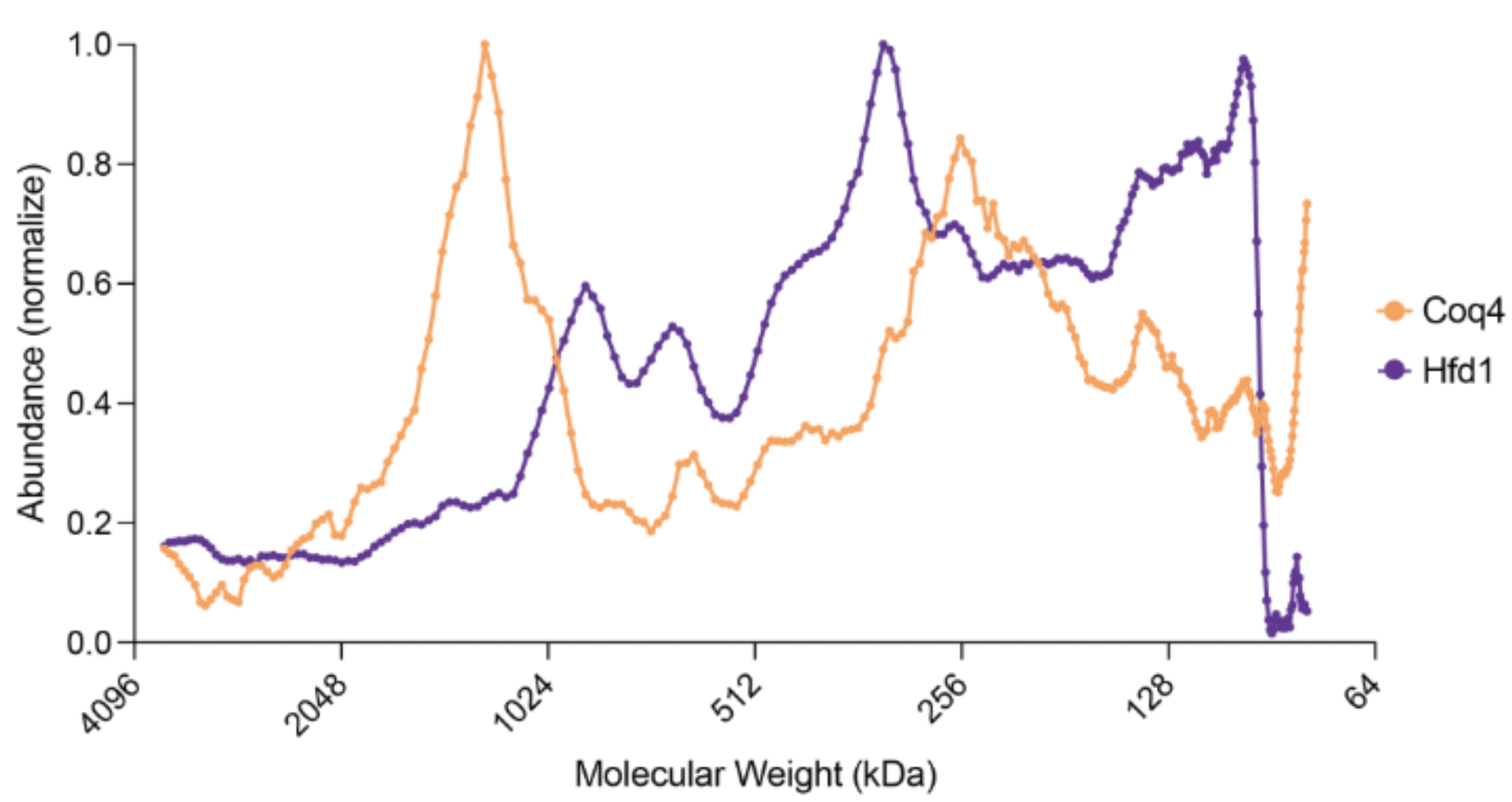

Response Figure 7. Normalized abundance profiles of Coq4 and Hfd1 as analyzed by high-resolution complexome profiling [5] .

Reviewer #3 (Remarks to the Author):
I read the manuscript with great interest and enjoyment. We have worked for over a decade on detecting protein–protein interactions by analyzing patterns in multiple sequence alignments. While we are well aware that such analyses can generate intriguing hypotheses about protein interactions and functions, successfully translating these hypotheses into experimentally validated discoveries is something we particularly appreciate. I do not have sufficient expertise to critically assess the experimental aspects of this work, so my comments below focus exclusively on the computational analysis.

We are glad that this reviewer found our manuscript interesting and enjoyable. We are particularly encouraged to receive this feedback from a Reviewer who has over a decade of expertise in coevolutionary analysis of protein–protein interactions. We appreciate their recognition that translating computational predictions to experimental validation, as we have done through two experimental vignettes, is a meaningful and a non-trivial contribution.

1. I appreciate that the authors evaluate performance using a signal-to-noise ratio of 1:100. This choice appears appropriate for a mitochondrial interaction screen, where each protein is expected to interact with perhaps 5–10 partners among roughly 1,000 mitochondrial proteins.

We thank the reviewer for this comment. We determined that this signal-to-noise ratio would be appropriate based on a prior study done for entire human proteome[1].

2. In some of our own benchmarks (not specifically on mitochondrial proteins), we found that iPTM can be a weaker indicator of interactions than other metrics, such as contact probability derived from distogram outputs or iPAE. I was wondering whether the authors explored alternative indicators and, if so, how their performance compared.

We compared the performance of ipTM, pDockQ, interface RMSD (iRMSD), ligand RMSD (lRMSD), and the iPAE scores after averaging across all 5 AFM models. On the Recent PDB dataset, both ipTM mean and pDockQ mean scores outperformed the other metrics which were derived directly from the predicted aligned error output of AFM (Response Fig. 8a).

We did not pursue pDockQ due to its known limitations with spurious interfaces. A recent study demonstrated that pDockQ performs worse than most alternative scores[10], including ipTM, when applied to full-length proteins under realistic conditions. Consistent

with this, we found that mean ipTM outperformed other metrics on our mitochondrial test dataset (Response Fig. 8b) and therefore adopted it as our scoring criterion for interaction screening.

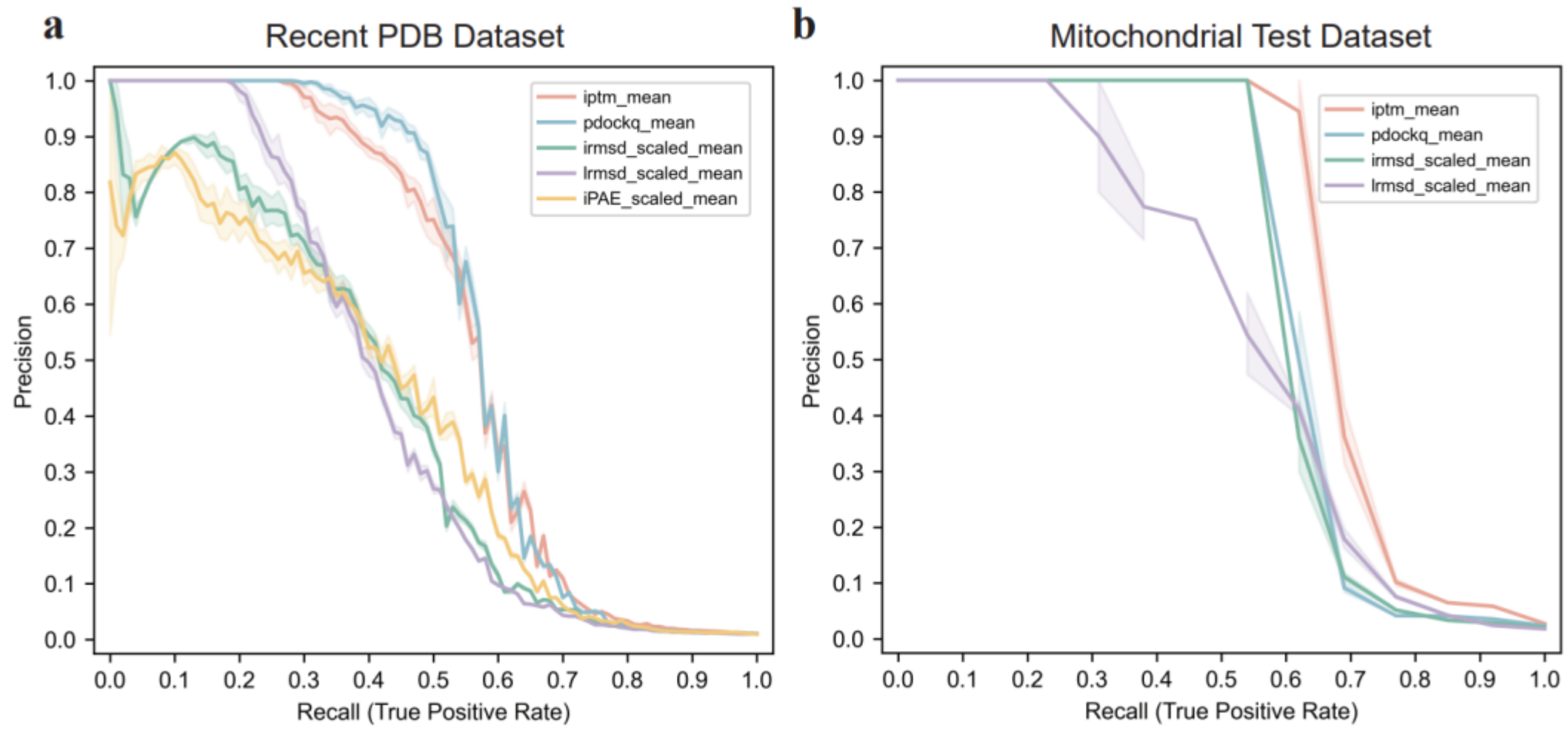

Response Figure 8: Precision-recall curves for the (**a**) recent PDB dataset and (**b**) mitochondrial test dataset, plotted using the indicated scores averaged across all five AlphaFold-Multimer models.

We did not evaluate the contact probability metric during our initial screening. However, a recent study using AlphaFold and RosettaFold reported that distogram contact probability at a 12Å cutoff can help distinguish true from false interaction pairs[1]. Due to storage constraints, we retained distogram logit outputs only for predictions with ipTM scores ≥ 0.5, which prevents a comprehensive benchmark of this metric across the full dataset. Nevertheless, we retrieved the contact confidence probabilities for the 2,895 MitoMatch hits and found that ~93% have scores ≥ 0.9 (Response Fig. 9). This further supports the robustness of the MitoMatch predictions, which score highly across multiple confidence metrics.

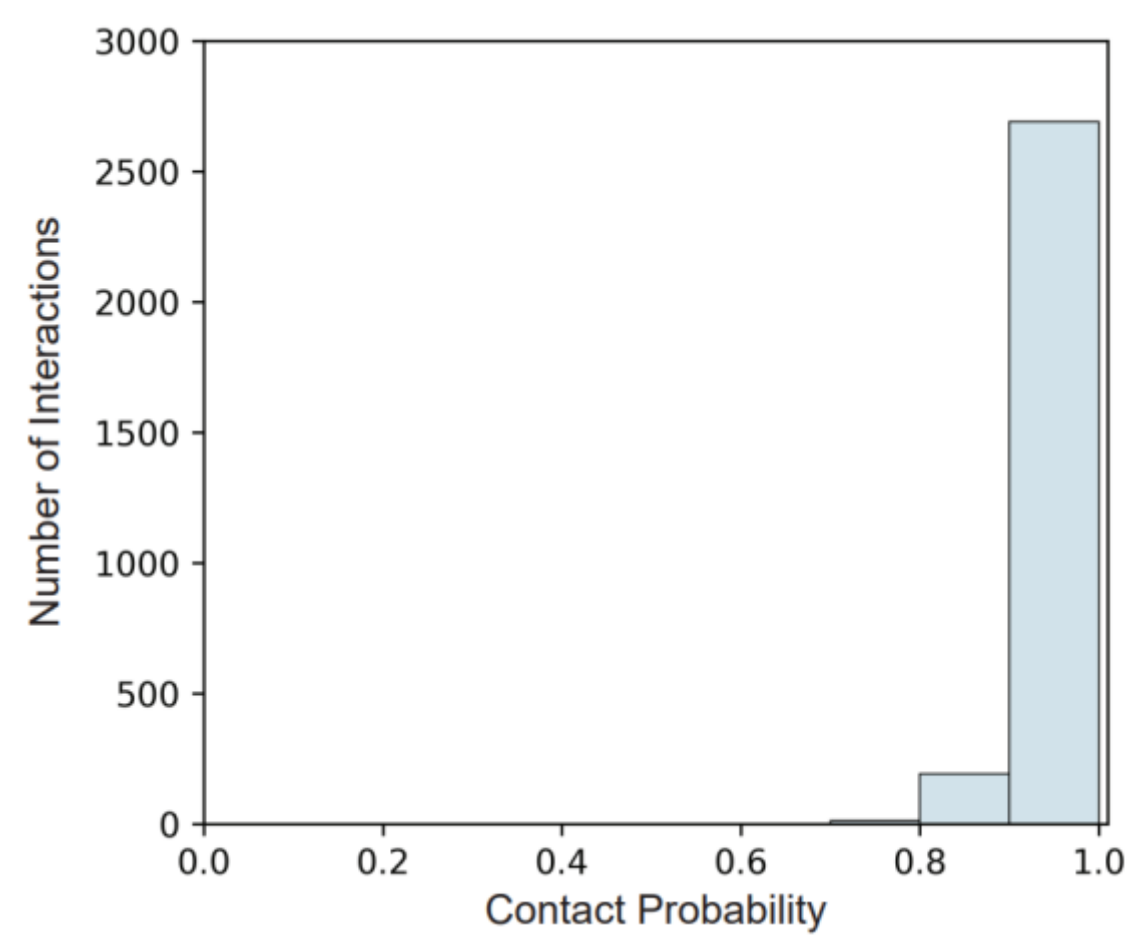

Response Figure 9: A histogram of the number of MitoMatch interactions as a function of the AlphaFold-Multimer contact probability score.

3. I am curious whether integrating predictions across multiple organisms might further improve performance. I understand that a comprehensive benchmark could be computationally demanding, but cross-

species integration may provide a promising avenue to enhance PPI prediction accuracy.

We thank the reviewer for suggesting additional metrics to increase prediction accuracy. We have now added both a species score and a conservation score to help prioritize hits based on evolutionary conservation (Revised Table S3). The species score is calculated as the sum of ipTM scores across all species containing homologs of both proteins in the query pair. The conservation score represents the fraction of homologous pairs identified as hits in the screen (ipTM ≥ 0.5). Higher scores in both these metrics indicate greater confidence in the predicted interaction across species.

We also note that, in some cases, a human interacting protein pair may have homologous pairs in other species, especially the evolutionarily distant species, that are not true interactors in those species. Therefore, while these scores provide additional context for interpreting the predictions, they should not be considered definitive filters for false positives.

4. In our own large-scale screens, we frequently observe systematic errors that limit performance. In particular, some proteins appear to have an intrinsic tendency to produce high interaction scores with many partners. I wonder whether the authors have observed a similar effect in their analysis and, if so, how they addressed it.

We had earlier looked at the number of interactors predicted for each protein in our screen and this measure should directly correlate with the "stickiness" of the protein that this reviewer has alluded to. However, we understand that this does not directly measure if there is an intrinsic tendency for that protein to produce higher scores for many proteins – and therefore higher interacting partners. To address this, we devised a new score called the baseline score, which are simply the z-score of the average iptm scores for all target proteins that are not hits (ipTM <0.5) for a given query (Response Fig. 10). For a protein A screened against 'n' targets:
Baseline score (A) = (Bulk ipTM- μ)/σ

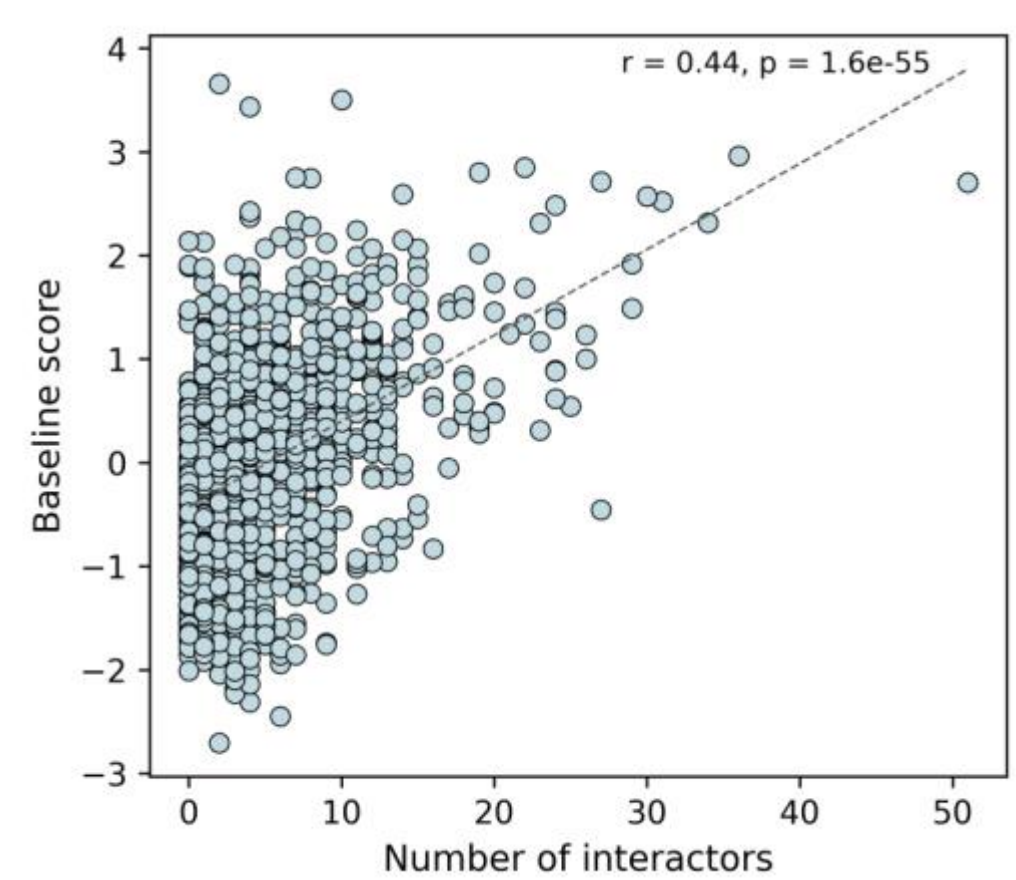

Response Figure 10: A plot of baseline score for each protein as a function of the number of interactors for that protein predicted through AlphaFold-Multimer

Where,

Bulk ipTM of A = average of ipTM scores of A that are less than 0.5

μ = average of the Bulk ipTM scores of all proteins
σ = standard deviation of the Bulk ipTM scores of all proteins

Since this is a z-score-based metric, a baseline score of 3.0 indicates that the average ipTM score for a protein is three standard deviations above the population mean. This metric is now included in the revised Table S3.

5. Another recurring issue we encounter is that homologous protein pairs tend to contribute disproportionately to false positives. Relatedly, if protein A interacts with protein B, we often observe elevated scores between A and B′, a paralog of B. These issues may be less pronounced in the mitochondrial context, where close homologs are relatively uncommon, but I would nonetheless be interested in the authors' experience regarding this potential source of error.

We work with the paralogous proteins SCO1 and SCO2, which participate in the mitochondrial copper delivery pathway. Although these proteins share similar properties, they perform distinct biological functions. For example, both SCO1 and SCO2 are cuproproteins that are metalated by COX17, and MitoMatch correctly predicts both SCO1–COX17 and SCO2–COX17 interactions. In vitro studies from our laboratory also show that both proteins can bind COA6, which is likewise captured by MitoMatch through prediction of SCO1–COA6 and SCO2–COA6 interactions. However, it is well established that only SCO1 functions as the bona fide copper metallochaperone for the cytochrome *c* oxidase subunit COX2. Consistent with this biology, MitoMatch predicts the SCO1–COX2 interaction but not SCO2–COX2.

That said, we agree with the reviewer that some paralog-based predictions may represent false positives rather than genuine interactions. At the same time, the examples above demonstrate that paralog-associated predictions cannot be dismissed outright, as some likely reflect true biological interactions. To address this potential limitation while preserving potentially meaningful hits, we have now annotated cases in which one protein in a predicted interaction is paralogous to a protein in another predicted hit. Using this approach, we identified 520 such interactions out of 2895 hits and added a corresponding flag in both the Excel dataset (Supplementary Table 3) and the online resource to alert users.

References:

1. Zhang, J. et al. Predicting protein-protein interactions in the human proteome. *Science* **390**, eadt1630 (2025).

2. Hsieh, E. J. *et al.* Saccharomyces cerevisiae Coq9 polypeptide is a subunit of the mitochondrial coenzyme Q biosynthetic complex. *Arch Biochem Biophys* **463**, 19–26 (2007).
3. Marbois, B., Gin, P., Gulmezian, M. & Clarke, C. F. The yeast Coq4 polypeptide organizes a mitochondrial protein complex essential for coenzyme Q biosynthesis. *Biochim Biophys Acta* **1791**, 69–75 (2009).
4. He, C. H., Xie, L. X., Allan, C. M., Tran, U. C. & Clarke, C. F. Coenzyme Q supplementation or over-expression of the yeast Coq8 putative kinase stabilizes multi-subunit Coq polypeptide complexes in yeast coq null mutants. *Biochim Biophys Acta* **1841**, 630–44 (2014).
5. Guerra, R. M. & Pagliarini, D. J. Coenzyme Q biochemistry and biosynthesis. *Trends Biochem Sci* **5**, 463–476 (2023).
6. Schulte, U. *et al.* Mitochondrial complexome reveals quality-control pathways of protein import. *Nature* **614**, 153–159 (2023).
7. Nývltová, E., Dietz, J. V., Seravalli, J., Khalimonchuk, O. & Barrientos, A. Coordination of metal center biogenesis in human cytochrome c oxidase. *Nat Commun* **13**, 3615 (2022).
8. Swaminathan, A. B. *et al.* A yeast suppressor screen links Coa4 to the mitochondrial copper delivery pathway for cytochrome c oxidase. *Genetics* **221**, iyac090 (2022).
9. Dokládal, L., Stumpe, M., Pillet, B., Hu, Z., Garcia Osuna, G.M., Kressler, D., Dengjel, J., De Virgilio, C. Global phosphoproteomics pinpoints uncharted Gcn2-mediated mechanisms of translational control. *Mol Cell.* **81**,1879-1889.e6 (2021)
10. Schmid, E. W. & Walter, J. C. Predictomes, a classifier-curated database of AlphaFold-modeled protein-protein interactions. *Mol Cell* **85**, 1216-1232.e5 (2025).
